# Acetaminophen-induced *S*-nitrosylation and *S*-sulfenylation signalling in 3D cultured hepatocarcinoma cell spheroids†
†Electronic supplementary information (ESI) available: Supplementary data in the form of a pdf file and an Excel table are available free of charge at the publisher's site. ‘Supplementary Figures.pdf’ contains additional figures and detailed figure descriptions. Supplementary Table S1.xlsx contains 5 tables presenting detailed protein expression data. Table A – ‘Protein expression’ contains a list of all proteins identified/quantified in label-free proteomics experiments; Table B – ‘IPA analysis’ contains the results of the pathway analysis of differentially regulated proteins under APAP treatment; Table C – ‘SNO/SOH proteome’ contains the list of all unique, iodoTMT-containing peptides quantified in min. 2 biological replicates. Table D – ‘RedoxDB + dbSNO’ is a summary of the comparative analysis of all identified SNO/SOH proteins and sites with information deposited in publicly available repositories of cysteine modifications – RedoxDB and dbSNO. Table E – ‘Table 1_extended’ is an extended version of Table 1 from the main manuscript. See DOI: 10.1039/c5tx00469a


**DOI:** 10.1039/c5tx00469a

**Published:** 2016-03-02

**Authors:** Katarzyna Wojdyla, Krzysztof Wrzesinski, James Williamson, Stephen J. Fey, Adelina Rogowska-Wrzesinska

**Affiliations:** a Protein Research Group , Department of Biochemistry and Molecular Biology , University of Southern Denmark , Campusvej 55 , 5230 Odense M , Denmark . Email: adelinar@bmb.sdu.dk; b Tissue Culture Engineering Laboratory , Department of Biochemistry and Molecular Biology , University of Southern Denmark , Campusvej 55 , 5230 Odense M , Denmark

## Abstract

Acetaminophen (APAP) is possibly the most widely used medication globally and yet little is known of its molecular effects at therapeutic doses. Using a novel approach, we have analysed the redox proteome of the hepatocellular cell line HepG2/C3A treated with therapeutic doses of APAP and quantitated both individual protein abundance and their reversible *S*-nitrosylation (SNO) and *S*-sulfenylation (SOH) modifications by mass spectrometry. APAP treatment results in a late, transient increase in ATP production and a multiplicity of alterations in protein abundance and modifications. The majority of the differentially SNO or SOH modified proteins are found in the endoplasmic reticulum and cytosol, suggesting that the source of reactive species is there. The cellular response indicates: constraint of fatty acid metabolism; reduction in ribosome construction and protein synthesis (to conserve ATP); maintenance of glutathione levels (by increased synthetic capacity); and an increased NADPH production (*via* the pentose phosphate pathway). This response appears to be coordinated, directly or indirectly, by the canonical Wnt and Nrf2 signalling pathways. Combined with the known role of NAPQI, these studies suggest that the physiological and toxicological responses form a continuum: therapeutic doses of APAP produce reactive species and NAPQI in the cytoplasm but result in little permanent damage. The cell mounts a multifaceted response which minimises disruption and repairs are effected within a day or two. Higher doses of APAP lead to intensified reactive species production, which increasingly disturbs mitochondrial function and eventually leads to cell death.

## Abstract 2

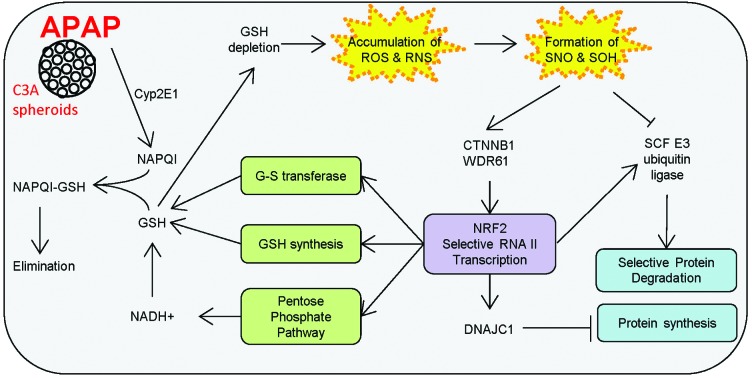
Acetaminophen (APAP) is possibly the most widely used medication globally and yet little is known of its molecular effects at therapeutic doses.

## Introduction

Acetaminophen (*N*-acetyl-*p*-aminophenol, APAP, paracetamol®) is a popular analgesic used worldwide by people of all ages. Administered within the recommended doses it is safe and causes only minimal side effects. However, APAP poisoning accounts for about half of all cases of acute liver failure in the USA and in Great Britain.^1^ Despite decades of extensive research into the toxicity of APAP, very little is actually known about the hepatocyte's metabolic response to APAP treatment at non-toxic (therapeutic) doses.

APAP is mainly metabolized in the liver by phase II conjugating enzymes to nontoxic products (acetaminophen-sulphate or -glucuronide) or by the phase I cytochrome P450 family of enzymes (specifically CYP2E1, CYP1A2, and CYP3A4). CYP2E1 in particular has been shown to generate the toxic *N*-acetyl-*p*-benzoquinone imine (NAPQI), a reactive metabolite that binds to cysteine residues in cellular proteins.^2^,^3^ Toxic doses of APAP lead to saturation of the sulphate and glucuronide pathways and to an increased production of NAPQI. This leads to glutathione (GSH) depletion, and the NAPQI is thought to cause hepatotoxicity by forming acetaminophen–protein adducts.^4^*N*-Acetylcysteine treatment can prevent or limit liver injury by restoring hepatic GSH concentrations.^4^

Thus, while earlier research suggested that NAPQI and its protein adducts gave rise to hepatotoxicity and that glutathione (GSH) had a protective role, recent research has shown that low levels of these products are formed even after administering therapeutic doses (4 g daily) to trial participants.^5^ At these therapeutic APAP doses, cytosolic NADPH:quinone oxidoreductase 1 (NQO1) is strongly upregulated^6^ and detoxifies NAPQI by reducing the quinone to hydroquinone (using NADPH in the process).^7^ At the same time NAPQI is rapidly and irreversibly conjugated with GSH.^8^ Currently, it is considered that excess NAPQI–protein adducts cause mitochondrial oxidative stress. Increasing levels of NAPQI stimulate reactive metabolite formation (*e.g.* Reactive Oxygen Species, ROS), which induces structural alterations in mitochondrial proteins and DNA. At high stress levels this causes a loss of mitochondrial membrane potential, cessation of ATP synthesis and an initiation of cell death. The release of cytochrome C and other proapoptotic factors promotes caspase activation and apoptotic cell death.^9^

In view of the role played by reactive metabolites, we analysed protein oxidation in order to probe deeper into the effects of APAP treatment. Superoxide (O_2_˙^–^) is a known by-product of NAPQI metabolism. Its neutralisation by intracellular antioxidant systems leads to the formation of hydrogen peroxide (H_2_O_2_) and peroxynitrite (ONOO^–^),^9^ both of which are capable of modifying proteins. Cysteine thiols are highly susceptible to oxidation by these species and can form *e.g. S*-sulfenic acid (SOH) and/or *S*-nitrosothiol (SNO).

SOH is predominantly generated by the spontaneous reaction of cysteines with H_2_O_2_. SOH is highly reactive and short-lived and may be irreversibly oxidised to sulfinic (SO_2_H) and sulfonic acid (SO_3_H). Therefore SOH is a marker of oxidative stress-related damage.^10^

SNO is formed by either the direct interaction of cysteines with nitrogen species *e.g.* peroxynitrite or results from transnitrosylation (the transfer of the nitroso group from *S*-nitrosylated small molecules or proteins). SNO modification can alter enzyme activity and protein–protein interactions, mediate protein localisation and alter protein stability.^11^

SNO and SOH are well recognised for their role in the regulation of protein activity and protein–protein interaction and thereby provide an additional avenue for intracellular signalling.^12^ Therefore we have re-investigated the APAP mode of action using a recently developed mass spectrometry-based SNO/SOH TMT method.^13^ This approach enables site-specific quantitation of SNO and SOH simultaneously, with concurrent correction of modification levels by protein abundance changes. The sensitivity of SNO/SOH TMT allows the detection of endogenous oxidation levels of the individual proteins.^13^

Understanding the molecular response to APAP treatment needs to be investigated in models relevant to human pathophysiology. Cell lines derived from human hepatocellular carcinomas are an inexpensive and reproducible source of essentially unlimited amounts of material for study. However, HepG2 and its daughter strain HepG2/C3A (hereafter denoted C3A) are generally regarded as poor models for hepatotoxicology.^14^–^16^ This perception is based on the use of cell culture techniques that disfavour the cellular expression of functions seen *in vivo*. When grown as 3D spheroids, and without using special media, growth factors or other chemicals (*e.g.* DMSO), C3A restore, over an 18 day period, cholesterol, urea, ATP, glycogen production and growth rates seen *in vivo*.^17^ Ramaiahgari *et al.* obtained very similar results using the closely related HepG2 cell line cultivated in Matrigel under 3D conditions, where they showed increased expression of albumin, xenobiotic transcription factors, phase I and II drug metabolism enzymes and transporters.^18^ The hepatic features recovered by growing cells in various types of 3D culture systems have been reviewed recently.^19^,^20^ These metabolic features are stable for at least 24 days.^17^,^18^ C3A 3D spheroids retain epigenetic markers that are seen in the liver but lost when the C3A cells are cultivated under 2D conditions.^21^ This recovery of physiological performance when grown in 3D is not limited to HepG2 but is also seen with other cell lines, for example, HepRG^22^ and many other cell types (reviewed in ref. 17 and 23). Most importantly, indirect comparison of LD_50_ values for *in vitro* and *in vivo* toxicity showed that data from C3A spheroids correlated better with *in vivo* observations than did studies using traditional two dimensional cultures of primary human hepatocytes (for 5 commonly used drugs including APAP).^24^

We hypothesise that the redox imbalance triggered by APAP metabolism would alter levels of SNO and SOH protein modifications. Many of these types of protein modifications are transient and could be involved in rapidly regulating the cellular non-hormonal intracrine signalling and response to APAP.

## Materials and methods

### Materials

HepG2/C3A were obtained from American Type Culture Collection (cat. no. CRL10741, the third passage after receipt from ATCC, Manassas, Virginia). D-MEM (1 g glucose per L), non-essential amino acids, penicillin/streptomycin, GlutaMAX, Trypsin/EDTA, and 0.4% trypan blue were all purchased from Gibco (Carlsbad, California); AggreWell™ 400 plates (Stemcell Technologies (Grenoble, France)) cell culture bioreactors (MC2 Biotek (Hørsholm, Denmark)), opaque microtitre plates (Nunc, Roskilde, Denmark; cat. no. 165306), and CellTiter-Glo luminescent cell viability assay (Promega, Fitchburg, WI). iodoTMT™ tags, immobilised and unbound anti-TMT™ antibody, Tris-buffered saline (TBS), tris(2-carboxyethyl)phosphine (TCEP), TMT-Elution Buffer, Pierce Screw Cup Spin Columns, Zeba Spin desalting columns and BCA assay were from Thermo Scientific. FCS (foetal calf serum), Acetaminophen (APAP) and the remaining chemicals were from Sigma Aldrich. All solvents were of analytical or higher grade.

## Methods

For all reagents, the final concentrations are reported, unless stated otherwise. All steps from oxidation induction to quenching after iodoTMT™ labelling were performed under minimal light exposure.

### 3D cell culture

The C3A spheroids were prepared as previously described.^17^ Briefly, C3A cell spheroids were prepared using AggreWell™ 400 plates (Stemcell Technologies cat. no. 27845). Spheroids were cultured in microgravity bioreactors (MC2 Biotek, Hørsholm, cat. no. 010), the humidity chamber was filled with distilled sterile water, and the growth chamber was prewetted with growth medium for 24 h before use by rotation in the incubator. Bioreactors were turned at appropriate speeds using the 16 axel BioArray Matrix drive BAM v4 (CelVivo, Blommenslyst). Spheroids were detached from the AggreWell™ plates, visually selected, placed in the microgravity bioreactors and cultivated in DMEM (1 g glucose per L) at 37 °C, 5% CO_2_, 95% air for a minimum of 21 days, exchanging the medium every two to three days.

### APAP treatment

*In vivo* studies are usually defined by grams of the compound per kilogram (kg) body weight of the organism. In the laboratory, since it would be difficult to weigh the cells, a convenient equivalent is milligrams of compound per milligram of total soluble cellular protein present. Assuming that in a 70 kg adult, the liver weighs 1.5 kg (ref. 25) and contains 0.5 kg blood,^26^ then by assuming that 60% of the liver is hepatocytes and that each cell contains 18% by weight protein,^27^ it is possible to calculate that the liver contains 108 g hepatocyte proteins. Since the recommended dose for APAP is 4 g per day and that 100% of the dose reaches the liver after 50 minutes,^28^,^29^ this gives a physiologically relevant dose of 0.03 mg APAP per mg hepatocyte protein. A 21 day old spheroid has an average volume of 0.5 μL and contains 21.31 μg protein. Since 150 spheroids were used in each 10 ml bioreactor, 5 mg APAP dissolved directly into 10 mL D-MEM growth medium at 37 °C (bioreactor volume) provides an equivalent dose to the recommended dose *in vivo*.

The following APAP final concentrations were used: 0, 2.5, 5, 10 and 20 mg APAP per mg cellular protein (referred to as mg mg^–1^). For the time-course experiment, mature C3A spheroids were treated with repetitive doses of APAP at 0, 48, 96, 144, 192 and 240 h, *i.e.* providing fresh APAP at the same time for each medium change. All treatments and controls were performed in duplicate (two 10 ml bioreactors were used for each treatment point).

Because the plasma half-life of paracetamol is 1.5–2.5 h (ref. 28) it was decided to collect samples immediately before and two, four and 24 hours after APAP treatment. Combining this sampling regimen with the 48 h media-exchange protocol resulted in samples being collected at 0, 2, 4, 24, 48, 50, 52, 72, 96, 98, 100, 120, 144, 146, 148, 168, 192, 194, 196, 216, 240, 242 and 244 h.

### ATP assay

The ATP production rate was used to measure viability of both control and APAP-treated spheroids as previously described.^24^ Briefly, spheroids (usually 2 spheroids per sample) were collected at 2, 4, 24, 48, 72, 96, 120, 144, 168, 196, 216 and 240 h. Cell viability was measured using the Cell-Titer-Glo luminescent cell viability assay (Promega, cat. no. G7571). The experiment was carried out in triplicate. The ATP content of the spheroids was normalised with reference to a standard curve for ATP, the untreated control and the amount of cellular protein present.

### Protein determination in spheroids

The amount of protein present in each sample was determined using the fluorescence-based ProStain Protein Quantification Kit (Active Motif Inc., cat. no. 15001) as previously described.^17^

### Time-course analysis of global oxidation changes in C3A spheroids upon APAP treatment

Both reversible and irreversible oxidations were measured using western blot. Reversible cysteine oxidations, SNO and SOH were detected after iodoTMT™ zero labelling. Protein carbonylation was detected after derivatization with 2,4-dinitrophenylhydrazine (DNPH).

Each bioreactor vessel contained exactly 150 spheroids. The treatment dose was 0, 2.5 and 5 mg APAP per mg cellular protein. 30 spheroids from each treatment group were sampled at 24, 48, 96 and 144 h, and washed twice with warm HANKS and once with PBS, allowing the spheroids to sediment naturally to the bottom of the tube each time. Washed spheroids were lysed in a lysis/free-thiol blocking buffer containing 150 mM HEPES, pH 7.3, 1 mM EDTA, 0.1 mM neocuproine, 2% SDS, and 50 mM MMTS.

For SNO/SOH analysis all the steps from lysis/blocking until western blot detection and visualization were performed as previously described.^13^ For carbonylation analysis, DNPH derivatisation and detection were performed using the OxyBlot™ kit (Merck Millipore) as previously described.^30^ Density analysis was performed using Image Studio Lite v. 3.1 (LI-COR Biosciences) as previously described.^30^ Briefly, the optical density of each lane was normalised to the averaged total density of all the lanes on the gel and expressed as percentage of the optical density. At each treatment time point the data were normalised to the control. Statistical analysis of the density data was performed using one-way ANOVA (*p* < 0.05) and the *t*-test (*p* < 0.05) built in the Microsoft Excel 2010 package.

### IodoTMT™-6plex labelling

The SNO/SOH TMT strategy utilising iodoTMT™-6plex reagents was used for the analysis of APAP-treated C3A spheroids as previously described.^13^

6 bioreactors containing 150 spheroids each (approx. 4 mg protein) were used for experiments. Cells in 3 bioreactors were treated with 5 mg APAP per mg cellular protein for 48 h and the other 3 bioreactors were used as control samples (triplicate biological replicates). After treatment, spheroids were washed twice with HANKS and once with PBS. Both the control and treated spheroids were resuspended in lysis/blocking buffer and were lysed by sonication. All the steps from lysis/blocking until anti-TMT™ enrichment and sample clean up were essentially the same as previously described.^13^ Several minor alterations were applied for improvement. To maximise the efficiency of free thiol blocking, 6 ml of lysis/blocking buffer were used for lysis. The samples were sonicated using 6 rounds of 20 s ON/5 s OFF at an amplitude of 50% in a tip sonicator (Q500, Qsonica). After acetone precipitation, protein pellets were washed 4 times with ice cold acetone before re-suspension in AENS buffer (50 mM AMBI, pH 8, 1 mM EDTA, 0.1 mM neocuproine, 2% SDS) to maximise removal of unbound MMTS.

### nLC MSMS analysis

nLC MSMS data were acquired using Dionex UltiMate® 3000 Rapid Separation UHPLC interfaced *via* an Easyspray nano-electrospray source to an Orbitrap Fusion™ Tribrid™ mass spectrometer (Thermo Scientific). The trapping column was 100 μm ID, 2 cm long, packed with 5 μm reverse phase C18 material (Reprosil, Dr Maisch, Germany). The analytical column was a Thermo Scientific Easyspray column of 75 μm ID, 50 cm long, packed with 2 μm C18 particles. The loading solvent was 0.1% trifluoroacetic acid (TFA), analytical solvents A and B were 0.1% formic acid (FA) in water and 0.1% FA in acetonitrile (ACN). All separations were carried out at 50 °C. Samples were re-suspended in 1% TFA, 5% DMSO and loaded onto the trap column at 10 μl min^–1^ for 5 min. Peptides were eluted onto and separated over the analytical column with the linear gradient of solvent B (2–35%) for 90 min. An accelerating voltage of 2.1 kV was applied for electrospray. The quadrupole isolation width was set at 1.6 Da. MS1 scans in the *m*/*z* range 300–1200 were acquired in the Orbitrap with 120 000 resolution at an AGC target of 5E5 and a maximum injection time of 60 ms. Tandem mass spectra were acquired in top speed mode with the cycle time set at 3 seconds at a normalised collision energy (NCE) of 38. MS2 spectra were acquired in the Orbitrap at 30 000 resolution, an AGC target of 2E4 and a maximum filling time of 65 ms. Dynamic exclusion was set at 45 s. Each biological replicate was analysed with duplicate injections (technical replicates).

### Database search

MS data were searched against the UniProt human proteome (40 779 entries, July 2, 2014) with common contaminants from MaxQuant (231 entries) using the Mascot server from within Proteome Discoverer version 1.4. The following search parameters were used: enzyme – trypsin; 2 missed cleavages; precursor mass tolerance – 10 ppm; and fragment mass tolerance – 20 mmu; variable modifications were – iodoTMT™ (Cys), carbamidomethylation (Cys) and oxidation (Met). For each biological replicate, search results from the 2 technical replicates were merged together. Duplicate features (peptide sequence + modification) were removed based on descending ion score values.

### Normalisation and significance analysis of redox proteomics data

Ratios of respective reporter ion intensities (127/126, 129/128 and 131/130) were log 2 transformed and normalised to their median value. Normalised SNO (129/128) and SOH (131/130) ratios were corrected by the corresponding ratios of total cysteines (127/126). Rank-based selection criteria were used to identify SNO/SOH sites that change significantly between the control and APAP treatment. The most stringent (rank 1) was a 2 sigma approach, which implies that the changes in individual SNO/SOH levels, corrected by changes in protein abundance levels, are more than two times the standard deviation for the particular modification subset, *e.g.* SNO. Peptides with 2 sigma values for a minimum of 2 biological replicates were included in rank 1 group.

Peptides with a SNO and/or SOH fold change equal to or greater than ±1.5 (log 2 ± 0.585) when observed in at least 2 biological replicates were selected as rank 2 peptides. Rank 2 proteins/peptides were predominantly used as additional evidence to support biological processes revealed by the rank 1 group (2 sigma analysis). Peptides with SNO/SOH quantitative values from APAP treatment only were selected as candidates for rank 3. Selection required that the respective reporter ions (129 for SNO and 131 for SOH) were present in at least 2 biological replicates at an intensity ≥1000.

We have quantified 8 iodoTMT™-containing peptides (11 SNO/SOH sites) from 8 proteins which fall in rank 1 (>2 sigma) for quantitative abundance change between control and APAP-treated spheroids. For instance, for the fatty acid synthase (FASN) the SNO levels of C1448 and C1459 were above 2 sigma in all 3 biological replicates. Rank 2 (>1.5-fold change) selected 41 SNO and/or SOH modified peptides. For one protein (EEF2) we found a rank 2 peptide additional to those found in the rank 1 subset. The remaining rank 2 peptides represented a further 40 modified proteins. Finally, the rank 3 group included 13 unique iodoTMT™-containing peptides (21 SNO and/or SOH sites) from 13 proteins. Altogether, we quantified 61 APAP sensitive SNO/SOH proteins.

### Modification assignment

Modification (SNO, SOH, SNO + SOH) was considered present if respective ratios were available for at least 2 of the 3 biological replicates.

### Label-free quantitative proteomics

A fraction of MMTS-blocked protein samples in AENS, from each iodoTMT™-6plex experiment, was used for the label-free analysis. Both the control and APAP treated samples (200 μg protein per condition) were digested on a filter using sodium deoxycholate (SDC)^31^ as previously described.^13^ After digestion, peptides were recovered by centrifugation (14 000*g*, 5 min) and SDC was removed by precipitation with TFA and phase transfer into ethyl acetate.^32^

nLC MSMS analysis and database search were performed as for the iodoTMT™-6plex experiment, with few modifications. For MS2, fragment ions were acquired in the ion trap using a rapid mode with an AGC target of 5E3 ions, a maximum injection time of 35 ms and the NCE of 32. In the database search, fragment mass tolerance was 0.6 Da, cysteine carbamidomethylation was used as a fixed modification and methionine oxidation as a variable modification. Quantitative information from label-free experiments was retrieved from nLC MSMS data using the chromatographic alignment feature within Progenesis LC MS (Nonlinear Dynamics) as previously described.^13^ Significance analysis was performed using the ANOVA test built into Progenesis LC MS software. Proteins with at least 3 quantified peptides and ANOVA significance value *p* ≤ 0.01 were considered significant.

### Data availability

The mass spectrometry proteomics data have been deposited in the ProteomeXchange Consortium *via* the PRIDE partner repository with the dataset identifier PXD003587.

### Functional and gene ontology (GO) analyses

String (Search Tool for the Retrieval of Interacting Genes/Proteins, v. 9.1) was used to generate functional networks of all the mapped SNO/SOH proteins.^33^ The following parameters were used: a confidence score of 0.900, no text mining and no disconnected proteins. Proteins were manually assigned to biological processes and/or molecular functions based on UniProt annotations. Ingenuity® Knowledge Base (v. 21249400) was used in functional analysis of differentially expressed proteins (from label-free analysis) and differentially regulated SNO/SOH proteins. A GO enrichment analysis was performed using GOrilla.^34^ The reference dataset used consisted of all the proteins identified in label-free proteomics analysis (*n* = 3). The significance, *p*-value threshold was *p* < 0.001. Reported *p*-values are enrichment probability values corrected (multiplied) by the number of tested GO terms (7530 for process and 1034 for component analyses).

## Results

The major objective of this study was to characterise the cellular response to APAP treatment using doses comparable to the recommended doses used *in vivo* (*i.e.* therapeutic doses at which the drug-induced changes are reversible). To do this, we studied the APAP-induced changes in the redox proteome of C3A spheroids. This *in vitro* model has been shown to be stable (on the basis of gene expression, protein abundance and physiological performance) over at least 24 days^35^ and provides a faithful reflection of *in vivo* toxicology.^24^ We measured simultaneously both the changes in abundance of proteins and the changes in their SNO/SOH PTMs. The experimental workflow is illustrated in ESI Fig. S1.†

Time-course experiments measuring cellular ATP levels and global changes in protein SOH, SNO and carbonylation were initially performed to establish the optimal treatment conditions to be used in the analysis of the C3A redox proteome.^13^

### Redox balance and cell viability upon APAP treatment

**Fig. 1:**
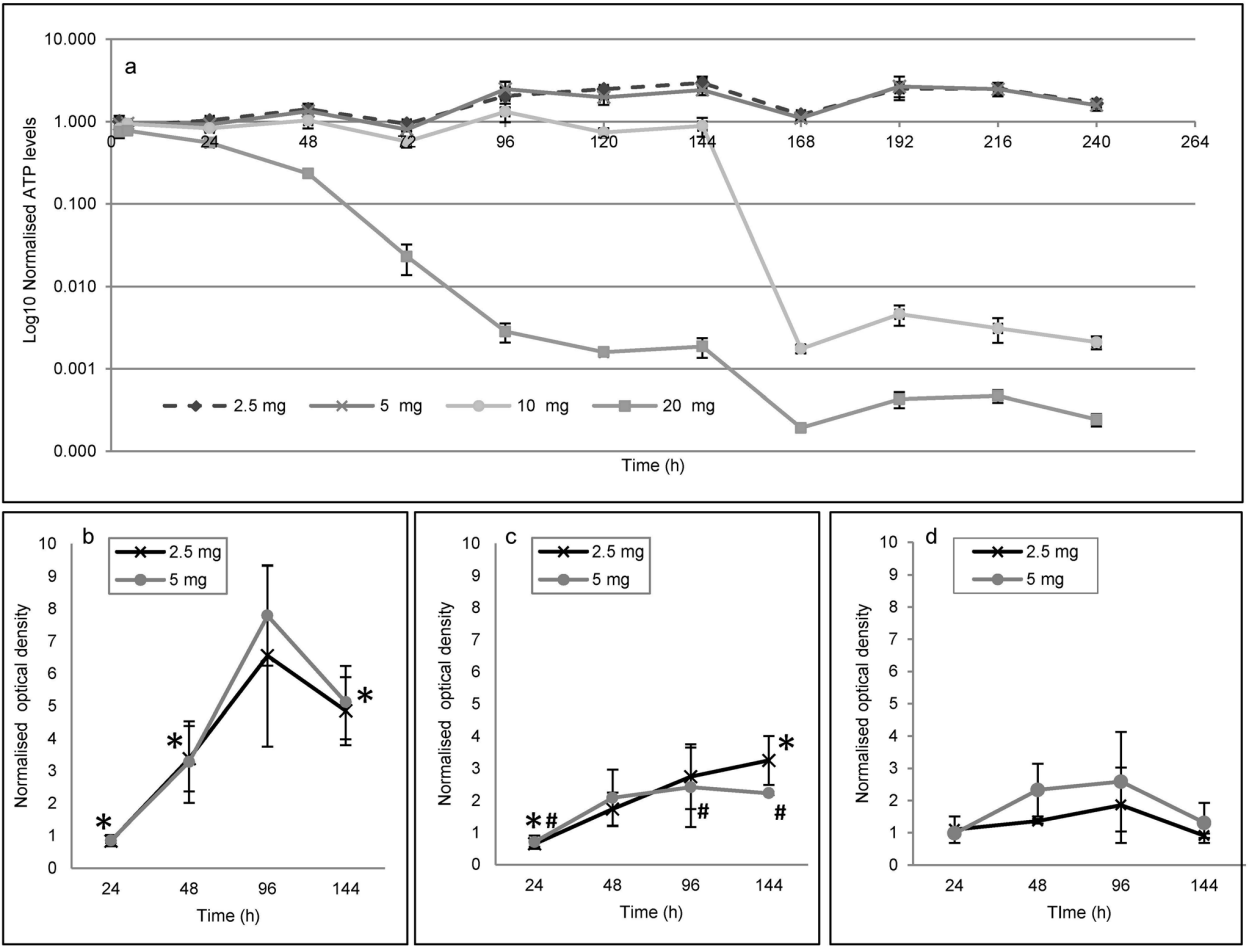
Time-course analysis of protein oxidation changes in C3A spheroids upon APAP treatment. Cell viability was accessed by the ATP production assay (a). The analysis, for 4 different APAP doses, was performed over 10 days with measurements taken every 24 h. Due to the significantly increased cytotoxicity, doses 10 and 20 mg APAP per mg soluble cellular proteins (mg mg^–1^) were excluded from subsequent analyses of protein oxidation changes. APAP-induced changes in reversible cysteine modifications, *S*-nitrosylation (b) and *S*-sulfenylation (c) were determined using iodoTMT™-zero labelling and western blot detection with anti-TMT™ antibody. (d) Protein carbonylation was measured after DNPH derivatisation and western blot detection with anti-DNPH antibody. Protein oxidation levels were visualised with chemiluminescence and optical density analysis was performed using Image Studio Lite, v.4.0. Error bars indicate standard error. Significant differences in SNO levels were detected after 48 h treatment with 2.5 and 5 mg of APAP (marked by * in panel b). Levels of SOH were significantly changed after 144 h of treatment with 2.5 mg APAP (indicated by * in panel c) and after 96 h and 144 h in 5 mg of APAP concentration (indicated by # in panel c). Statistically significant differences were calculated using a *t*-test (*p* < 0.05, *n* = 3).

The viability of C3A spheroids following APAP treatment was measured by ATP production.^36^ Similar results were obtained by measuring adenylate kinase (ref. 17 and data not shown). The *in vivo* recommended dose of 4 g per day for an adult corresponds to an *in vitro* treatment dose of 5 milligrams APAP per milligram cellular hepatic soluble protein (mg mg^–1^). The analysis was therefore performed using 0, 2.5, 5, 10 and 20 mg APAP per mg. Treatment was carried out every 48 h (Fig. 1a) and ATP levels were measured every 24 h for 10 days.

For the lower APAP doses (2.5 and 5 mg mg^–1^), we observed fluctuations in the ATP levels over time, characterised by slight drops immediately after treatment followed by recoveries, showing intensified ATP production from 24 to 48 h after treatment as the cell presumably repairs the damage done (Fig. 1a). The overall cell viability was not affected throughout the treatment period and ATP levels returned to baseline levels in the absence of further APAP treatment. Contrary to this, at higher APAP doses (10 and 20 mg mg^–1^) ATP production diminished greatly, indicating the onset of cytotoxicity and lethality. Since the goal of this study was to focus on the effects of physiological doses of APAP, the doses of 10 and 20 mg APAP per mg cellular protein were excluded from subsequent analyses.

As the next step in defining the optimal conditions for the study, we analysed the overall redox homeostasis using 2.5 and 5 mg APAP per mg cellular protein at the time points 24, 48, 96 and 144 h. Changes in protein oxidation were monitored by SNO and SOH TMT western blot (showing reversible protein oxidation, Fig. 1b and c respectively) and carbonyl western blot assay (irreversible protein oxidation, Fig. 1d). Our results show that both doses induce a progressive accumulation of SNO modification over time, which apparently peaked at about 96 h (Fig. 1b). This was surprising considering that the biological half-life of APAP is only 2 h. A similar trend of progressive accumulation was also observed for SOH (even though the magnitude of changes was considerably lower). Significant differences in SNO levels were detected after 48 h of treatment with 2.5 and 5 mg of APAP (Fig. 1b). SOH levels also changed significantly after 144 h of treatment with 2.5 mg APAP and after 96 h and 144 h in 5 mg of APAP (Fig. 1c). Interestingly, at 2.5 mg mg^–1^ the accumulation of SOH was lower and the oxidation levels did not appear to saturate, even after 144 h (Fig. 1c).

The largest, simultaneous increase in SNO and SOH levels was observed at 96 h after cell treatment with 5 mg APAP per mg cellular protein. However, at this point, cell activity was already affected as indicated by the strong increase in ATP production (Fig. 1a). At 5 mg mg^–1^ after 48 h there was only a minimal effect on ATP production and essentially no observed increase in the irreversible protein carbonylation levels (Fig. 1a and d). Under these conditions increases in both SNO and SOH levels were observed. Therefore, this treatment (5 mg APAP per mg cellular proteins for 48 h) was used for all subsequent experiments. At that level, 6 consecutive APAP treatments had no effect on the overall viability of the spheroids during a 10 day treatment regimen (Fig. 1a).

### APAP-induced changes in protein expression

**Fig. 2:**
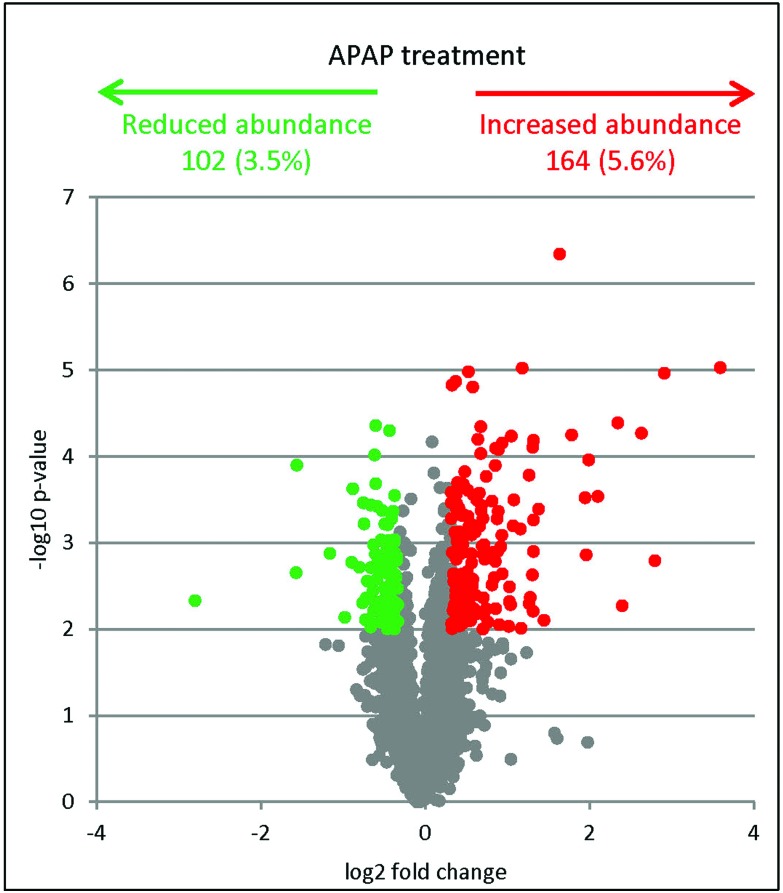
A volcano plot representing proteins changed in abundance after treatment with 5 mg APAP per mg cellular protein for 48 h. The significance cut-off was set at *p* < 0.01 (ANOVA test), and the biological cut-off was set to a fold change of ±1.2 fold. Colours are used to represent insignificant proteins (grey), both biologically and statistically significant proteins being up-regulated (red) and down-regulated (green).

Next we sought to investigate the effect of APAP treatment on the C3A proteome using label-free quantitative mass spectrometry. In total, 2953 proteins were quantified. There was no significant change in abundance between control and APAP treatment for the large majority of proteins detected (Fig. 2). Only 266 proteins (approx. 9%) showed differential regulation (*n* = 3, ANOVA *p* ≤ 0.01, minimum fold change 1.2). In response to APAP treatment, 164 proteins increased in abundance, while the remaining 102 decreased in abundance (a list of all the quantified proteins is provided in ESI Table S1A†).

Ingenuity® Pathway Analysis revealed that the energetic balance of the cells was significantly affected by APAP treatment as indicated by the increased abundance of enzymes belonging to the pentose phosphate pathway (ESI Table S1B†). In addition, there was a significant decrease of several enzymes belonging to the EIF2 signalling pathway (including 9 ribosomal proteins and 5 translation initiation factors, suggesting that APAP treatment affected protein synthesis); an increase in several proteins involved in the NRF2-mediated oxidative stress response pathway and an increase in the abundance of three glutathione *S*-transferases GSTT1, GSTK1, GSTM3 and the related GGH (1.53, 1.20, 1.49 and 1.32 fold respectively). The observed protein abundance changes illustrate activation of antioxidant machinery and increased energy production that are both required to neutralise the effects of APAP treatment.

### Redox proteome map of C3A spheroids

**Fig. 3:**
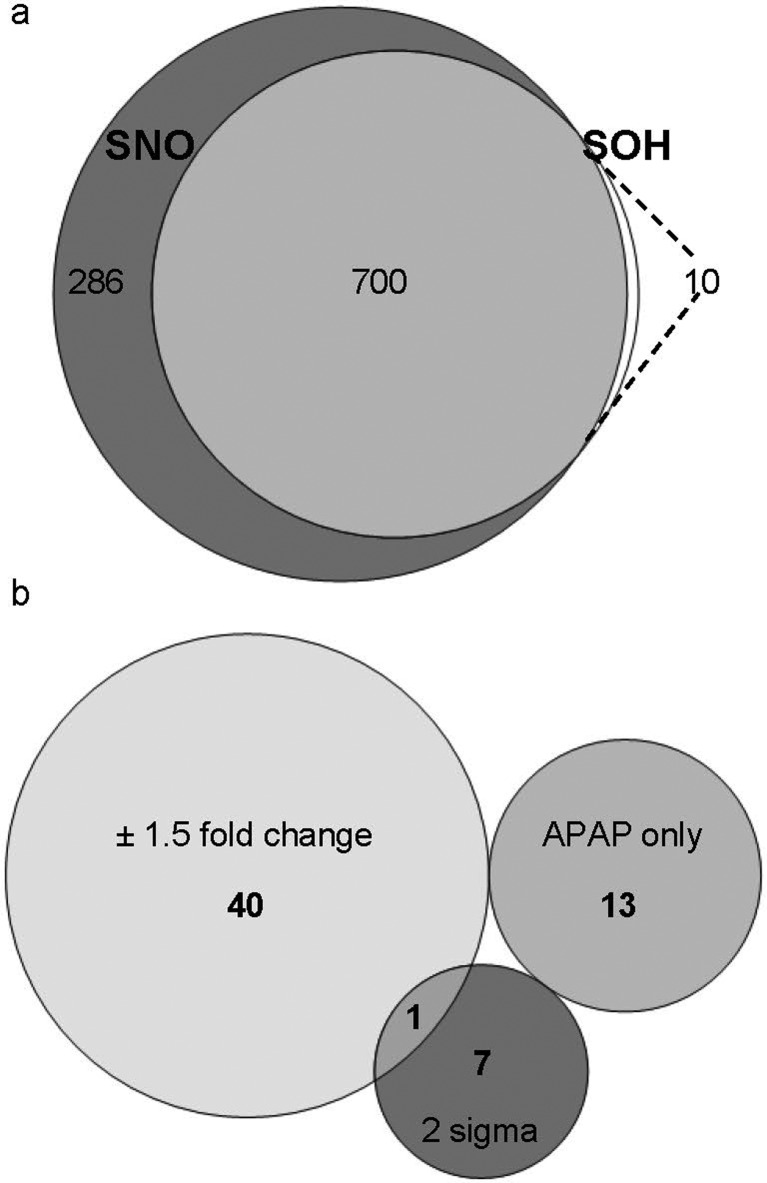
SNO/SOH proteome of C3A spheroids under control and APAP treatment (5 mg APAP per mg cellular protein for 48 h). (a) Overlap between SNO (dark grey) and SOH (white) sites identified in C3A spheroids under basal conditions. (b) APAP-sensitive SNO/SOH sites were selected with rank-based criteria, as defined in the Materials and methods section.

Prior to APAP treatment, we had mapped the SNO/SOH post-translational protein modifications in C3A spheroids. Altogether, we have identified 887 unique, iodoTMT™ containing peptides with SNO, SOH or both modifications (listed in ESI Table S1C†). These peptides correspond to 996 modification sites on 569 different proteins. The majority of modification sites were identified with both SNO and SOH modifications (Fig. 3a). Concurring with the western blot analysis, the relative abundance of SNO was on average higher than that of the corresponding SOH (ESI Fig. S2 and S3†).

The identified SNO/SOH proteins in the C3A proteome belong to basic molecular pathways, *e.g.* protein synthesis and degradation, fatty acid metabolism and glycolysis/gluconeogenesis, as illustrated in ESI Fig. S4.†

Taking into account known SNO/SOH modifications from the curated databases RedoxDB^37^ and dbSNO,^38^ our study has identified 737 SNO and 695 SOH novel sites corresponding to 479 SNO and 453 SOH modified proteins (ESI Fig. S5 and Table S1D†). Thus, this study is the most comprehensive inventory of SNO/SOH sites and proteins observed in human hepatocarcinoma cells to date. These SNO/SOH modified proteins were used as a baseline for APAP treatment studies.

### Alterations in SNO and SOH modification levels upon APAP treatment

Complete quantitative information from SNO/SOH TMT analysis was used to determine APAP sensitive SNO and/or SOH sites within oxidised proteins (ESI Table S1E†).

Considering all unique iodoTMT™-containing peptides, the average SNO occupancy in the control was 1.55% ±0.20 increasing to 2.00% ±0.43 following APAP treatment. For SOH, the average occupancy in the control was 0.56% ±0.15 and this was unchanged following APAP treatment (0.57% ±0.16, ESI Table S1C†). There was no correlation between the changes in protein modification levels and the percentage of site occupancy (ESI Fig. S6†).

To identify the APAP sensitive sites, we applied rank-based selection criteria (Fig. 3b and Table 1). ESI Table S1E† contains an extended version of Table 1; annotated MSMS spectra and assigned ion series masses of all modified peptides are available in ESI Fig. S8†.

**Table 1:** Functional grouping of proteins containing cysteines modified by SNO and/or SOH and regulated upon APAP treatment. An extended table is available in ESI Table S1

UniProt Accession	Protein name (UniProt)	Gene name (primary)	Primary cellular location(s)^*a*^	SNO/SOH site^*b*^	Rank^*c*^	SNO^*d*^	SOH^*d*^
RNA and mRNA processing							
Q00839	Heterogeneous nuclear ribonucleoprotein U	HNRNPU	Nucl	607	1		Up
P42704	Leucine-rich PPR motif-containing protein,	LRPPRC	Nucl/Mito	130	2	Up	
Q9GZT3	SRA stem–loop-interacting RNA-binding protein	SLIRP	Mito	48	2	Up	
P55769	NHP2-like protein 1	NHP2L1	Nucleolus	30	2		Down
Protein synthesis							
P13639	Elongation factor 2	EEF2	Cyto	290	1	Up	
P13639	Elongation factor 2	EEF2	Cyto	369	2		Up
P41250	Glycine–tRNA ligase	GARS	Cyto/Mito	461	3		Up
P41252	Isoleucine–tRNA ligase	IARS	Cyto	336	1		Down
P55884	Eukaryotic translation initiation factor 3 subunit B	EIF3B	Cyto	302	2		Up
P23396	40S ribosomal protein S3	RPS3	Cyto	134	3		Up
P62280	40S ribosomal protein S11	RPS11	Cyto	116	2		Up
P36578	60S ribosomal protein L4	RPL4	Cyto	250	2	Up	
P30050	60S ribosomal protein L12	RPL12	Cyto	162	3	Up	
P62888	60S ribosomal protein L30	RPL30	Cyto	52	2	Up	
Q9Y6G3	39S ribosomal protein L42	MRPL42	Mito	45	2	Down	
Protein folding and trafficking							
Q9Y3B3	Transmembrane emp24 domain-containing protein 7	TMED7	ER/Cyto/Golgi	59; 75	1	Up	
P55735	Protein SEC13 homolog	SEC13	ER/Cyto/Golgi	234	2	Up	
P78371	T-complex protein 1 subunit beta	CCT2	Cyto	412	1		Down
P55145	Mesencephalic astrocyte-derived neurotrophic factor	MANF	Secretory	151	2		Down
Lipid metabolism							
P49327	Fatty acid synthase	FASN	Cyto/Golgi	1448; 1459	1	Up	
Q8NBQ5	Estradiol 17-beta-dehydrogenase 11	HSD17B11	Cyto/Secretory	215; 217	1	Up	
P14324	Farnesyl pyrophosphate synthase	FDPS	Cyto/Mito/Nucl	333, 340	3	Up	
O95573	Long-chain-fatty-acid–CoA ligase 3	ACSL3	Mito/Perox/ER	450	2		Up
O60488	Long-chain-fatty-acid–CoA ligase 4	ACSL4	Mito/Perox/ER	221	2		Up
P37268	Squalene synthase	FDFT1	ER	147	2		Down
WNT signalling							
Q9HB71	Calcyclin-binding protein	CACYBP	Nucl/Cyto	154	2	Up	
Q9GZS3	WD repeat-containing protein 61	WDR61	Nucl/Cyto	303	1		Up
P35222	Catenin beta-1	CTNNB1	Cyto/Nucl	520	2		Up
Q86VP6	Cullin-associated NEDD8-dissociated protein 1	CAND1	Cyto/Nucl	237	2	Up	
P63244	Guanine nucleotide-binding protein subunit beta-2-like 1	GNB2L1	Cyto	168	2	Up	
Q9UK22	F-box only protein 2	FBXO2	Cyto	71	2	Down	Down
P63208	S-phase kinase-associated protein 1	SKP1	Cyto	120	2		Down

^*a*^Primary cellular location(s) according to UniProt: cyto, cytoplasm or cytosol; nucl, nuclear; mito, mitochondrial; ER, endoplasmic reticulum; Golgi, golgi apparatus; perox, peroxisome; secretory, secreted, vesicles, extracellular matrix.

^*b*^SNO/SOH site – amino acid number within the protein sequence that has been modified by SNO and/or SOH.

^*c*^Rank – criteria used to select proteins with significant changes in the SNO and/or SOH modification levels upon APAP treatment were as follows: 1 – peptides with 2 sigma values for a minimum of 2 biological replicates; 2 – peptides with SNO and/or SOH fold change equal to or beyond ±1.5 (log 2 ± 0.585) if observed in a minimum of 2 biological replicates; 3 – peptides that contained only reporter ions corresponding to samples treated with APAP, the respective reporter ions (129 for SNO and 131 for SOH) were present in min. 2 biological replicates at an intensity ≥1000.

^*d*^SNO and SOH – regulation trend observed in quantitative redox proteomics experiments for the particular modification type, up/down – modification abundance increased/decreased in cells treated with APAP.

Significance analysis revealed that less than 10% of all the quantified iodoTMT™-containing peptides were differentially *S*-nitrosylated and/or *S*-sulfenylated as a result of APAP treatment. Gene Ontology (GO) annotation enrichment analysis strongly points to the cytosol/cytoplasm as the most significantly enriched cellular localisation (GO: 0005829; *p* = 1.09 × 10^–7^), while the most heavily overrepresented group was proteins present in extracellular organelles *e.g.* extracellular membrane vesicles (GO: 0043230; *p* = 1.64 × 10^–27^). The latter group contained liver-specific proteins such as prothrombin (F2), fibronectin (FN1) and alpha-fetoprotein (AFP). In AFP, 8 SNO/SOH sites were identified, all of which are canonically involved in intrinsic disulfide bonds. The primary functional Gene Ontologies for rank 1 peptides were: RNA and mRNA processing, protein synthesis, protein folding and trafficking, lipid metabolism and canonical Wnt/β-catenin signalling pathway, as annotated in UniProt (Table 1).

**Fig. 4:**
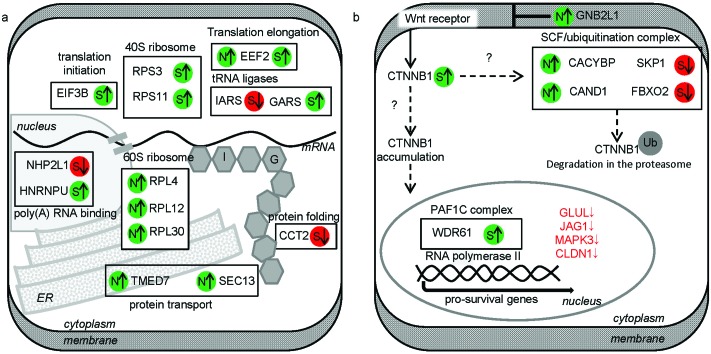
APAP-sensitive proteins related to protein synthesis (a). Proteins with cysteine SNO/SOH levels changed significantly between APAP treatment and control were used to map out the pathway based on manual inspection of UniProt annotations. Proteins are represented by their respective gene names. Changes in cysteine oxidations are marked with arrows. N – *S*-nitrosylation, S – *S*-sulfenylation, ↑ – min. 1.5-fold increase in abundance, ↓ – min. 1.5 decrease in abundance. I and G are abbreviations of amino acids, isoleucine and glycine respectively. The corresponding data on protein expression and SNO/SOH modification levels are provided in ESI Table S1.† APAP-sensitive proteins from the canonical Wnt/β-catenin signalling pathway (b). Proteins with cysteine SNO/SOH levels changed significantly between APAP treatment and control were used to map out the pathway based on manual inspection of UniProt annotations. Proteins are represented by their gene names. Changes in cysteine oxidations are marked with arrows. N – *S*-nitrosylation, S – *S*-sulfenylation, ↑ – min. 1.5-fold increase in abundance, ↓ – min. 1.5 decrease in abundance. Alternative routes of Wnt/β-catenin signal transduction are marked with dashed arrows. Protein names marked with arrows indicate proteins up (↑) or down (↓) regulated under APAP treatment. SCF – Skp, cullin, F-box containing complex (multiprotein E3 ubiquitin ligase complex); Ub – ubiquitination; PAF1C – RNA Polymerase-associated factor 1 complex. The corresponding data on protein expression and SNO/SOH modification levels are provided in ESI Table S1.†

Protein synthesis was the largest and the most diverse group affected, comprising 14 proteins (16 SNO/SOH sites). They covered a range of associated processes from RNA binding to ribosomal translation, protein folding and transport (Fig. 4a). In particular, the protein translation machinery was highly represented, containing 10 modified proteins (Table 1). In general, there was an increase in abundance of both SNO and SOH. The exceptions were NHP2-like protein 1 (NHP2L1), the cytoplasmic isoleucine–tRNA ligase (IARS) and the T-complex protein 1 subunit beta (CCT2) for which we observed a decrease in SOH abundance.

The canonical Wnt/β-catenin signalling pathway was also highly represented with 7 differentially modified proteins (Table 1). One of these was from rank 1 (SOH-peptide from WD repeat-containing protein 61 (WDR61)) while the other 6 proteins were rank 2 selected peptides. Of the latter group, 4 proteins were from the SCF (SKP1-CUL1-F-box protein) E3 ubiquitin ligase complex (Fig. 4b).

Six proteins involved in lipid synthesis and metabolism were also differentially modified (Table 1). All but one exhibited a significant increase in SNO/SOH levels upon APAP treatment, and this exception was squalene synthase (FDFT1). Two of the proteins bearing increased modification levels (FASN and FDFT1) require NADPH for their function.

In addition to the above functional groups, proteins modified upon APAP treatment were involved in glycolysis, gluconeogenesis, DNA damage and stress response. The list is provided in ESI Table S1B.†

The levels of SNO and SOH were decreased for 9 and 11 proteins respectively upon APAP treatment (ESI Table S1E†). This unexpected finding can be explained by the fact that cysteines oxidised to SNO or SOH can also become further oxidised to sulfinic (SO_2_H) and sulfonic acid (SO_3_H), indicators of severe, irreversible oxidation of proteins. These modifications cannot be detected by the SNO/SOH TMT method, but may contribute to the decreased signal of SNO or SOH.

### Cellular location of SNO/SOH modified proteins

In untreated control cells, SNO/SOH modified proteins are found distributed throughout the cell with no particular localisation being over- or under-represented (according to GO enrichment analysis, data not shown). However, after APAP treatment, the majority of proteins that show changes in SNO and/or SOH levels were found to be localised to the cytoplasm and endoplasmic reticulum (23 and 5 respectively), mitochondria (7) and nucleus (8) (Table 1). In addition, a number of differentially modified proteins were identified as proteins bound for secretion *e.g.* estradiol 17-beta-dehydrogenase 11 (HSD17B11). Please see ESI Fig. S7 and Table S1E† for more details.

### Site occupancy

The quantitative information obtained from SNO/SOH TMT analysis can also be used to estimate the levels of relative modification site occupancy (the fraction of the residue occupied by the identified modification).^13^ Such levels are considered to reflect important biological functions of SNO and SOH.^39^ For example, modification site occupancy defines levels of protection against irreversible oxidation or enzyme activity.^11^

In our study, site occupancy differs considerably from protein to protein (a complete list of site occupancy values for all SNO/SOH proteins is presented in ESI Table S1C†).

**Fig. 5:**
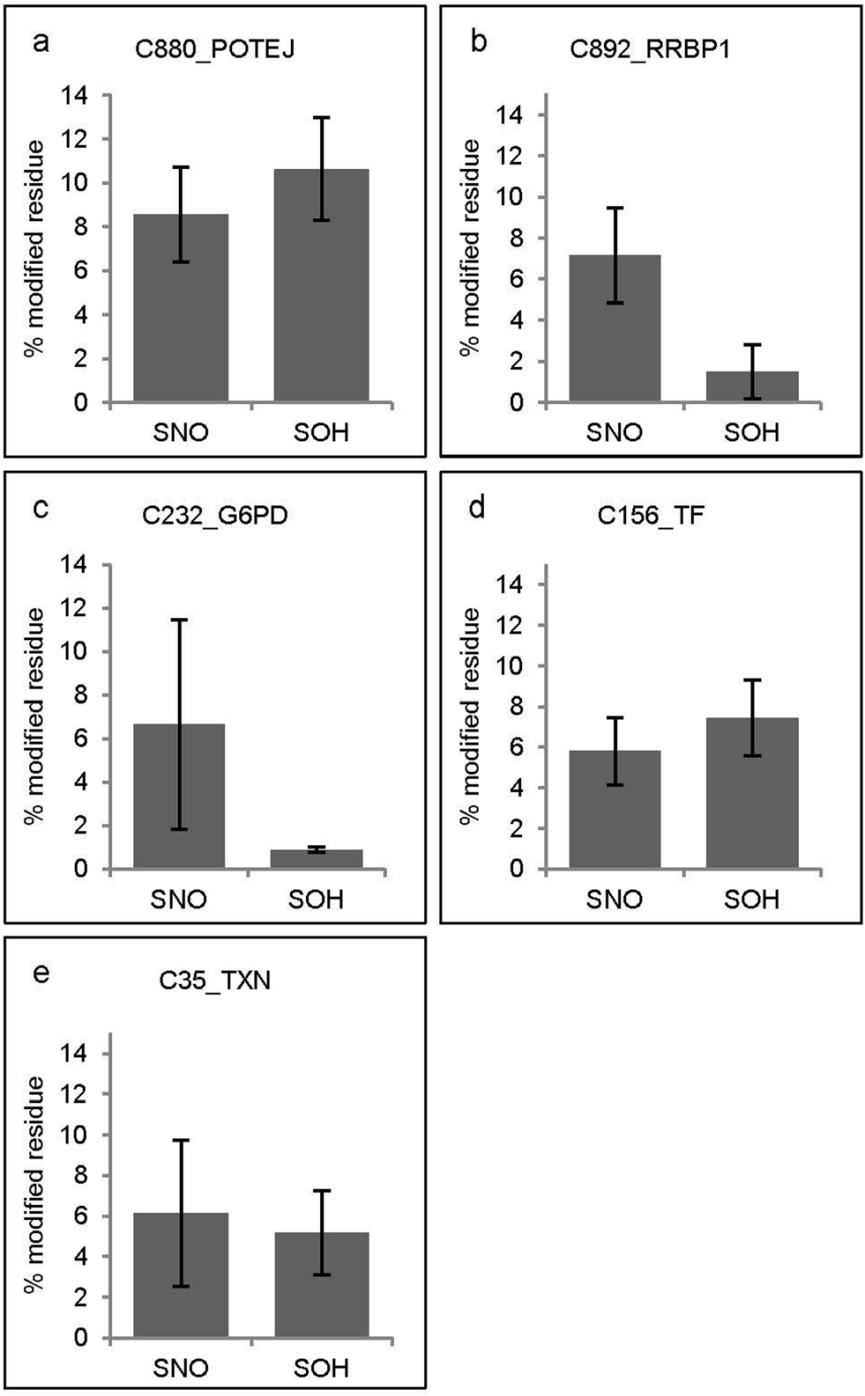
Proteins with the highest observed levels of relative site occupancy for SNO (a, b and c) and SOH (a, d and e) modifications in cells treated with APAP (5 mg APAP per mg cellular protein for 48 h). Average site occupancy values were calculated for min. 2 biological replicates, error bars correspond to ±standard deviation. Presented are gene names corresponding to the following proteins: POTEJ – POTE ankyrin domain family member J; RRBP1 – ribosome-binding protein 1; G6PD – glucose-6-phosphate 1-dehydrogenase; TF – serotransferrin; TXN – thioredoxin. The corresponding data for relative SNO SOH site occupancy levels for all sites and proteins identified in this study are provided in ESI Table S1C.†

In addition to examining the APAP-induced differentially modified cysteines, sites exhibiting high site occupancy were also considered. The cysteine site C880 from POTEJ (POTE ankyrin domain family member J) was the site with the highest oxidation occupancy upon APAP treatment for both modifications: 8.6 ±2.2% was SNO modified and a further 10.6 ±2.4% was SOH modified (Fig. 5a). In other words, almost a fifth (19.2%) of the C880 site was oxidised. The second most SNO modified site was C892 of the ribosome binding protein 1 (RRBP1, located in the endoplasmic reticulum) with 7.2 ±2.3% SNO occupancy (Fig. 5b). The third most SNO modified site was C232 from the cytosolic glucose-6-phosphate dehydrogenase (G6PD) where 6.6 ±4.8% of the site was modified (Fig. 5c).

Amongst *S*-sulfenylations, C156 from the secretion-destined sero transferrin (TF) was the second most modified site with 7.4% ±1.9 SOH (Fig. 5d). The C35 site from the antioxidant thioredoxin (TXN) was the third most abundant SOH site with 5.2% ±2.1 (Fig. 5e).

Within the top 10 SNO/SOH highest occupancy sites, 3 were extracellular/secreted proteins, the significance of which is unclear.

None of the cellular or extracellular proteins, which show high site occupancy, was differentially modified upon APAP treatment, underlining that the changes in SNO/SOH sites described above were highly specific.

### Structural features

We observed that some SNO/SOH modified peptides/proteins share some specific structural features, which suggests that the SNO and SOH could dramatically impact the protein's function. This included both differentially modified sites (APAP sensitive) and sites with the highest occupancy (oxidation susceptible). For instance, metal binding was a property of 20 of the 61 SNO/SOH containing proteins. In one case, the actual SNO/SOH cysteine was directly involved in metal ion coordination (C174 in ADH5 – a GSH-dependent formaldehyde dehydrogenase). This group of 20 metal binding proteins also included 5 cases where the modified cysteine participated in disulfide bond formation and thus may interfere with protein structural integrity^40^ (ESI Table S1E†).

Finally, two different proteins were observed where the modified cysteine plays a catalytic role: SNO and SOH modifications of the active site C248 from thiosulfate sulfurtransferase (TST) (ProSITE-ProRule annotation) and C35 from thioredoxin (TXN).^41^ Interestingly, although SNO and SOH levels of TXN did not change significantly, both were amongst those with the highest occupancy (6.1 ±3.6% and 5.2 ±2.1% respectively).

## Discussion

As described in the Introduction, hepatocarcinoma cells grown in 3D cultures have been shown to structurally, metabolically and physiologically resemble hepatocytes in the liver (reviewed in ref. 19, 20 and 23). For this reason, we would postulate that the response to pharmacological doses of APAP described in this study should provide a basic description of what occurs *in vivo*.

### APAP induces mild oxidative stress

In this study, we have used advanced mass spectrometric techniques to quantify global changes in abundance of proteins and their *S*-nitrosylation and *S*-sulfenylation levels in C3A 3D spheroids after exposure to therapeutic-equivalent doses of APAP (5 mg APAP per mg total soluble protein) to characterise the early stage response. C3A spheroids can be repeatedly treated with this dose regimen without reducing their viability. Measurements of ATP production illustrated that there was no significant reduction in ATP levels, suggesting no apparent disturbance of mitochondrial function. There was also no significant irreversible protein damage as determined by carbonyl western blot but we did observe increasing levels of reversible SNO and SOH modifications (which peaked after about 96 h).

In general, the proteomic data from both protein abundance and oxidative modification levels indicate a significant, but mild oxidative insult. The distributions of the changes in SNO and SOH modifications were highly specific and occurred in a number of low abundance proteins. The proteins involved fit well with existing data describing the effect of APAP on hepatocytes *in vitro* and *in vivo*^42^–^44^ and reveal a novel mechanism for signal transduction.

### Metabolism of APAP and the source of oxidative stress

The spatial location of proteins showing APAP-induced changes in SNO and SOH levels showed that many were found in the endoplasmic reticulum (ER) and the cytoplasm. Fewer were found in the mitochondria and nuclei and among proteins destined for secretion (Table 1 and ESI Table S1A and S1E†).

The localisation of modified proteins to the ER and cytoplasm is consistent with the observation that P450 cytochromes are a known source of reactive species following APAP treatment.^1^,^45^ The reactive species generated are primarily in the form of H_2_O_2_ and peroxinitrite (ONOO^–^) which are both known to induce SNO/SOH formation.^1^ The high reactivity and short half-life of peroxinitrite combined with its limited ability to diffuse across biological membranes^46^ contribute to its highly localised impact. In addition to H_2_O_2_ and peroxinitrite, NAPQI is also produced in the ER and is known to play a central role in APAP-induced hepatotoxicity.^2^,^3^,^47^

In the ER and cytoplasm, changes were seen in both protein abundance and oxidative modifications. In general, their abundance was decreased and they were heavily *S*-nitrosylated and/or *S*-sulfenylated. The ER protein, RRBP1, was the second most SNO modified protein (Fig. 5b). Additionally, two proteins directly associated with the ER, TMED7 and SEC13 possessed significantly increased SNO levels under APAP treatment (Table 1).

In the cytoplasm, the abundance of all 13 components of 40S ribosome, that were detected, was reduced on average by 1.25-fold (in a remarkably coordinated manner showing a standard deviation (SD) of only ±0.05). The same effect was seen for all of the 17 detected 60S ribosomal proteins (1.26-fold, SD ±0.07). Revealing a hitherto unexpected specificity, components of the 40S ribosome were differentially *S*-sulfenylated whereas all the identified proteins associated with the 60S ribosome were *S*-nitrosylated (ESI Table S1E†). This specificity can be extended to the initiation factor EIF3B which binds to and shows the same SOH modification as the small subunit whereas the elongation factor EEF2 interacts with both subunits and shows both SOH and SNO modifications.

In contrast, the level of SNO modification of the 39S mitochondrial ribosomal protein L42 was decreased. Two examples of mitochondrial proteins which show increased SOH modification were the long-chain acyl-CoA synthetases 3 and 4 (Table 1). These are both located in the outer mitochondrial membrane.

Together, the presence of APAP-inducible SNO/SOH modifications predominantly at the ER and associated ribosomes (assigned cytoplasmic location), as well as the apparent lack of significant oxidation in mitochondria and nuclei, suggests that, at least at the physiological doses of APAP used here, the source of the APAP-induced reactive species is located in or close to the ER.

### Recovery of the cellular redox balance

The recovery strategy that the cell appears to use in order to cope with alterations in the intracellular redox balance is to redirect its energy utilisation. The changes appear to be orchestrated by the Nrf2-mediated response pathway to oxidative damage and the canonical Wnt/β-catenin signalling pathway.

### The Nrf2-mediated response

Despite the fact that therapeutically relevant APAP doses were used, we observed an increase in the abundance of sixteen proteins involved in the Nrf2 mediated response to oxidative damage, indicating Nrf2 activation (ESI Table S1†).

In the mouse liver, APAP-induced mild oxidative stress results in the build-up of Nrf2 and its translocation into the nucleus, where it functions as a transcription factor.^48^ Once activated, Nrf2 has been shown to protect against APAP-induced hepatotoxicity^43^,^49^,^50^ and induce the production of Mrp3 and Mrp4 transporters, which may expel potentially toxic chemicals^51^ and initiate cell regeneration.^44^

One of the targets of Nrf2, the heterodimer glutamate cysteine ligase (composed of an enzymatic (GCLC) and a regulatory subunit (GCLM)) catalyzes the first and rate-limiting step in the production of GSH.^49^ Abundance of both of these enzymes was slightly increased (GCLC 1.31 and GCLM 1.15-fold). GSH synthase (GSS) catalyzing the second step was increased by 1.19-fold. Thus these changes in abundance may be the result of the protective Nrf2 transcription.^50^,^52^

Another enzyme HAGH that catalyzes the hydrolysis of *S*-d-lactoylglutathione to form GSH and d-lactic acid was also increased by 1.36-fold. Increases in GSH levels have been seen from 8 to 48 hours after acute APAP treatment in mice.^53^ Despite the increases in protein amounts, none of these GSH synthesis pathway proteins changed their SNO or SOH modification levels, suggesting that their regulation by oxidative stress occurred by Nrf2 regulating their synthesis.

The reactive APAP metabolite NAPQI can bind to the cysteine thiol of GSH. This critical mechanism of detoxification is mediated at least in part by phase-two metabolic enzymes of the glutathione *S*-transferase family^4^ and can lead to GSH depletion. The abundances of three glutathione *S*-transferases, GSTT1, GSTK1, GSTM3, and the related GGH were increased (1.53, 1.20, 1.49 and 1.32 fold respectively). While these changes appear to be modest, previous proteomic studies have shown that changes like these are typical and that they can produce significant alterations in cellular structure and activity.^30^ In a previous study comparing growth in 2D or 3D culture, we had observed that the protein levels of three other glutathione *S*-transferases were also modulated: the microsomal MGST1 was reduced by 2.4-fold while the cytosolic GSTK1 and GSTO1 were increased by 1.3 and 2.8-fold respectively, illustrating that the absolute levels of these enzymes are also culture specific.^30^

Wu *et al*. have demonstrated that Nrf2 can regulate NADPH generation and consumption,^54^ thus providing the link to regulation of the pentose phosphate pathway. There was an increase in enzymes of the pentose phosphate pathway. The abundances of most of its enzymes, G6PD, H6PD, PGD, TALDO1, and PGLS, were increased (1.82, 1.28, 1.26, 1.21 and 1.21-fold change respectively). The only exception was RPIA (which was reduced by –1.52-fold). G6PD is the key rate limiting enzyme of the pentose phosphate pathway and was the protein showing the highest increase in abundance. G6PD has not previously been reported as *S*-nitrosylated. Its cysteine C232 was found to be the third most *S*-nitrosylated site, although its relative occupancy did not change significantly between the control and APAP treatment. This might indicate that for this protein, feedback activation occurs *via* protein synthesis and not by modification. Evidence supporting the importance of the pentose phosphate pathway can also be found in TALDO1–/– mice developed by inactivation of the Taldo1 genomic locus.^55^ These are very sensitive to APAP treatment and exhibit a loss of mitochondrial membrane potential, reduced ATP/ADP ratio, and reduced β-catenin phosphorylation and spontaneously develop hepatocellular carcinomas. All of these features can be reversed by a lifelong treatment with *N*-acetylcysteine.^56^

Processes that require reducing power, *e.g.* NADPH and/or ATP, were also affected by mild APAP treatment. Changes in the SNO/SOH levels in proteins of the fatty acid synthesis/metabolism were pronounced following APAP treatment. FASN (the multi-enzyme protein that catalyses fatty acid synthesis) and FDFT1 (squalene synthase in the cholesterol synthetic pathway), both requiring NADPH for enzymatic activity, were differentially SNO/SOH modified under APAP treatment (5.6 and –1.8 average fold change). Similarly, two ATP driven long-chain-fatty-acid–CoA ligases (ACSL3 and 4, which convert free long-chain fatty acids into fatty acyl-CoA esters, and play a key role in lipid synthesis and fatty acid degradation) showed increased SOH modification after APAP treatment (2.5 and 1.5-fold). While the effect on enzyme kinetics cannot be deduced from these studies, it has been shown by several other groups that mitochondrial β-oxidation of fatty acids is impaired during APAP toxicity^53^ and our results would support this. Administration of carnitine, the carrier of fatty acids, into mitochondria provided protection from APAP toxicity, suggesting a link between fatty acid oxidation and APAP toxicity.^57^

### Wnt/β-catenin signalling is affected by oxidative stress

Here for the first time we report SNO and SOH modifications of components in the canonical Wnt/β-catenin signalling pathway. These changes are considered to be the cellular response to the oxidative insult rather than the primary ‘damage’.

Canonical Wnt/β-catenin signalling has been shown to be crucial in regulating the establishment of hepatic metabolic zonation^58^ as well as hepatoprotection and liver regeneration after drug-induced liver injury.^59^,^60^ PTMs, like phosphorylation and ubiquitination, have been shown to play an important role in regulating this pathway.^61^ In general, an APAP-induced increase in SNO/SOH was observed in all detected Wnt/β-catenin pathway-related proteins, with the exception of SKP1 and FBXO2, where levels decrease.

The major targets affected by SNO/SOH were the components of the SCF (Skp1/Cul1/F-box) ubiquitin ligase complex (SKP1, FBOX2, CAND and CACYBP). The SCF complex is a multicomponent E3 ubiquitin ligase, responsible for polyubiquitination and targeting of proteins for proteasomal degradation. While SNO/SOH modification of the SCF complex has not been described before, other E3 ubiquitin ligases have been shown to be inhibited by SNO modification.^62^

Both β-catenin and WDR61 exhibit an unchanged abundance but an increased SOH modification upon APAP treatment. WDR61 is a component of the PAF1 complex, which is responsible for the activation of transcription of Wnt genes by RNA polymerase II. The exact role of SNO modification of WDR61 is yet to be verified. Of importance might be the newly established link between Wnt signalling and the Nrf2/ARE oxidative stress response.^63^ A reduction in the binding of Nrf2 to β-catenin or a reduction in Nrf2 ubiquitination (and hence a reduction in its proteolytic degradation) could lead to its translocation into the nucleus and activation of G6PD and NQO2.

Corroborating this inactivation of β-catenin, we detected several other β-catenin responsive proteins in reduced abundance *e.g.* glutamine synthetase (GLUL) and MAPK3 (–2.9 and –1.2-fold).

Wnt signalling reduced the abundance of several proteins related to protein synthesis (including six eukaryotic translation initiation factors) by a factor of about 1.25. EIF3B was the only initiation factor where the degree of SOH modification was increased (1.5-fold).

The observed reduction in protein synthesis was mirrored by a reduction in all of the detected proteins associated with nucleolar ribosome assembly (RPS3, NOB1, TSR1, BRIX1 and DRG1 on average by 1.35-fold) and the reduced abundance of the ribosomal proteins (by 1.25 fold) noted above.

Given the low abundance of the SNO and SOH modifications in the cell and their transient nature, we hypothesise that SNO/SOH modifications of the Wnt/β-catenin pathway could represent a novel intracrine signalling mechanism. This mechanism could account for the Wnt/β-catenin-driven induction of the cytochromes CYP2E1 and CYP1A2 seen in mice^64^ and primary human hepatocytes^65^ and for the induction of liver regeneration seen in mice^44^,^59^ and thus provide evidence for a further protective strategy against APAP-induced acute liver injury (AILI).

### Modified secreted proteins

Intriguingly, we identified a number of SNO/SOH modified proteins which are destined to be secreted, *e.g.* MANF and HSD17B11. Where SNO/SOH proteins enter the extracellular space, they might spread oxidative damage due to the release of coordinated transition metals^66^ or *via* transnitrosylation.^11^ Finally, SNO/SOH modified proteins, when present in body fluids, would be expected to be ‘trapped’ in their modified format and therefore are potential candidate biomarkers reflecting acute or chronic use of APAP. They would be detectable in plasma, using strategies like SNO/SOH TMT^13^ or Quantitative Cysteine Oxidation Analysis by Multiple Reaction Monitoring (OxMRM).^67^ Such early stage indicators of reversible hepatic stress could be complementary to existing late stage markers such as alanine and aspartate aminotransferases whose presence in plasma is an indicator of irreversible liver damage.^1^

## Conclusions

**Fig. 6:**
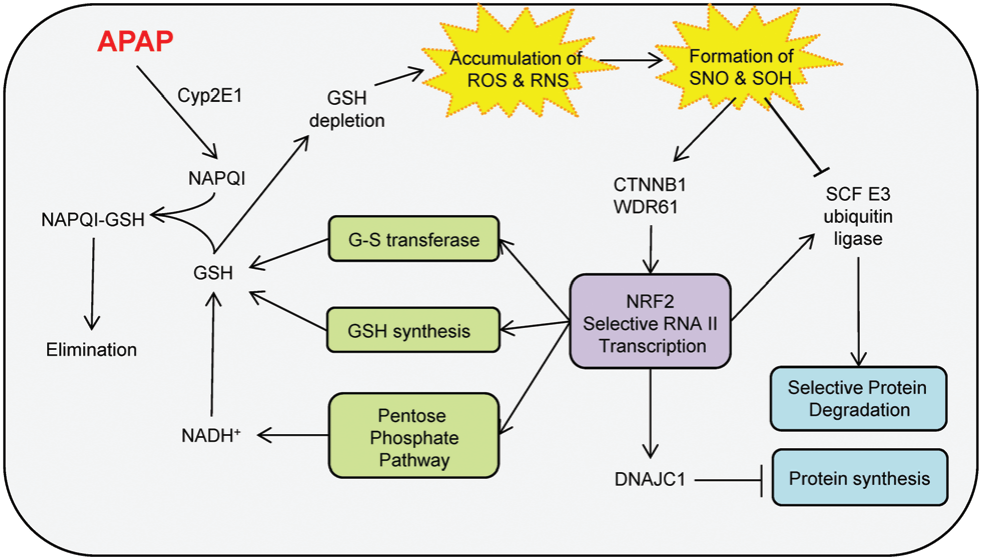
Summary of molecular pathways affected by APAP treatment (5 mg APAP per mg cellular protein for 48 h) observed using label free proteomics and SNO/SOH TMT. Detoxification of NAPQI leads to depletion of GSH and accumulation of endogenous RNS and ROS that contribute to generation of modified proteins. In response, cells activate pathways that lead to decreased protein synthesis and increased production of reducing power *via* the pentose phosphate pathway.

This is the first proteome-wide analysis of reversible cysteine modifications, *S*-nitrosylation and *S*-sulfenylation following APAP treatment. Our study provides the largest collection of SNO/SOH proteins resolving basal, APAP-susceptible and APAP sensitive modification sites and suggests that these modifications provide an additional novel signalling capacity allowing the cell to respond homeostatically to external stimuli like APAP treatment. By analysing the redox proteome of the hepatocarcinoma cell line C3A treated with a therapeutically relevant dose of APAP, we have revealed that the cell responds to APAP insult using a variety of strategies simultaneously (summarised in Fig. 6). While the depletion of GSH and the appearance of NAPQI are well known from acute toxicity studies, little or nothing is known about the activation of the pentose phosphate pathway, the general retardation of protein synthesis, and the molecular events leading to the activation of the Wnt/β-catenin and Nrf2 signalling pathways.

From these studies it appears that the strategic response of the cell to APAP-induced generation of reactive species (in or close to the ER) is the activation of the Wnt/β-catenin and Nrf2 signalling pathways and the disruption of fatty acid metabolism. These signalling pathways coordinate the maintenance of GSH and NADPH levels by diverting energy resources from ribosome assembly and protein synthesis to the pentose phosphate pathway as an effective measure to restore homeostasis. Altogether, our results help unify various observations on the effects of APAP treatment *in vitro* and *in vivo*.

## Funding information

This work was supported by the Sino-Danish Center for Education and Research and Augustinus Fonden (KWo); University of Southern Denmark (ARW); MC2 Biotek and University of Southern Denmark (KW and SJF); Villum Fonden Center for Analytical Biosciences (61310-0110-13761) and Center for Epigenetics, (62310-0112-023541) (ARW, KWo, JW).

## Author Contributions

Conceived and designed the experiments: ARW, KWo and KWr. Performed the experiments: KWr, KWo, JW. Analysed the data: KWo, ARW, KWr, JW, SJF. Contributed to the writing of the manuscript: KWo, ARW, SJF, KWr, JW.

## Supplementary Material

Supplementary information

Supplementary information
