# Supplementary material for: Acetaminophen-induced S-nitrosylation and S-sulfenylation signalling in 3D cultured hepatocarcinoma cell spheroids
Source: Toxicol Res (Camb). 2016 Mar 2;5(3):905–20. doi: 10.1039/c5tx00469a (PMC6072433; doi:10.1039/c5tx00469a)

Electronic Supplementary Material (ESI) for Toxicology Research.
This journal is © The Royal Society of Chemistry 2016

Supplementary Figures S1 – S7

Acetaminophen-induced S-nitrosylation and S-sulfenylation signalling in 3D cultured hepatocarcinoma cell spheroids.

Katarzyna Wojdyla[1, 3, *], Krzysztof Wrzesinski[2, *], James Williamson[1, 4], Stephen J. Fey[2], Adelina Rogowska-Wrzesinska[1]

[1] Protein Research Group Department of Biochemistry and Molecular Biology, University of Southern Denmark, Campusvej 55, 5230 Odense M, Denmark
[2]Tissue Culture Engineering Laboratory, Department of Biochemistry and Molecular Biology, University of Southern Denmark, Campusvej 55, 5230 Odense M, Denmark

[3] Present address: Cancer Institute, University College London, Paul O'Gorman Building, 72 HuntleyStreet, London, WC1E6DD, UK.
[4] Present address: Cambridge Institute for Medical Research, Wellcome Trust/MRC Building, Hills Road, Cambridge, CB2 0XY, UK

[*] These authors contributed equally

Corresponding author: Adelina Rogowska-Wrzesinska, adelinar@bmb.sdu.dk, Department of Biochemistry and Molecular Biology, University of Southern Denmark, Campusvej 55, 5230 Odense M, Denmark

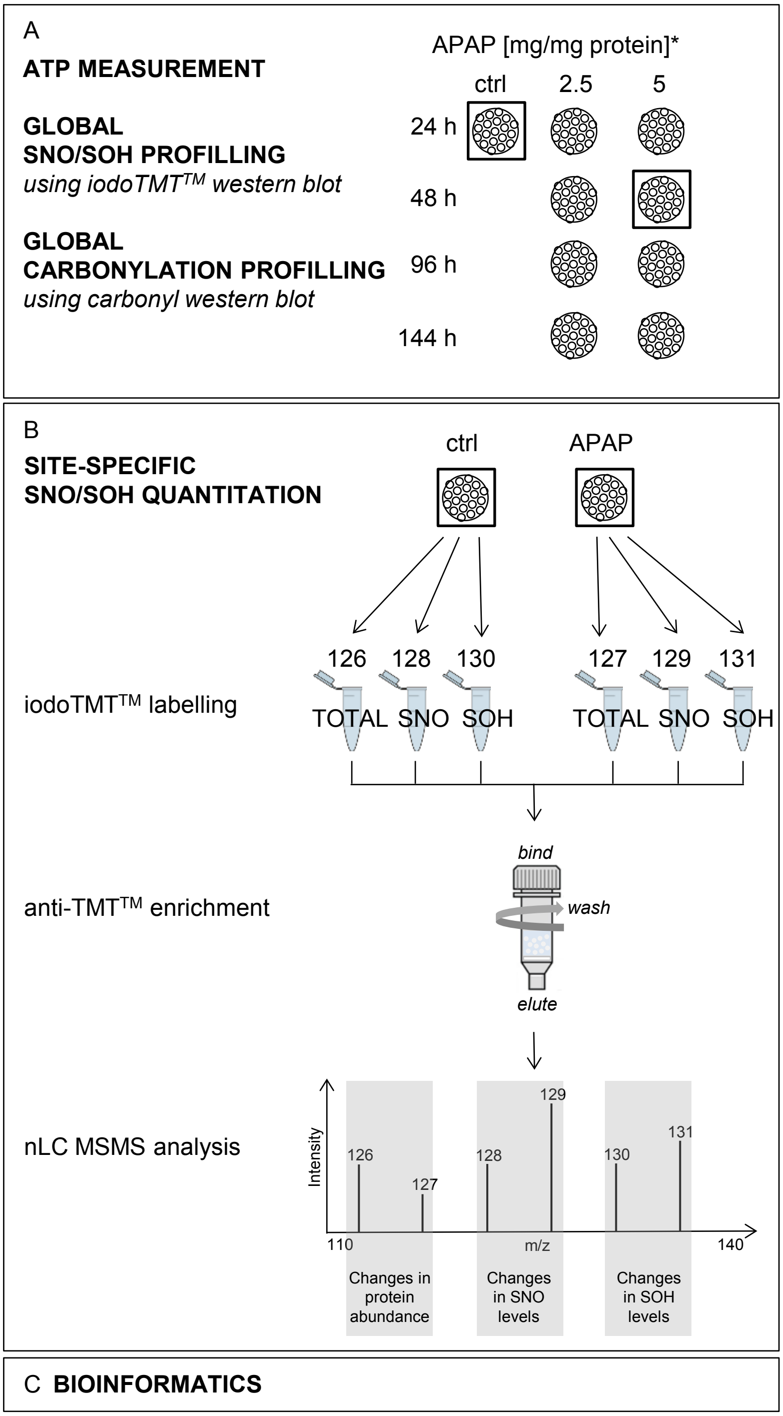

Figure S1. Overview over the analytical strategy applied to characterise the redox proteome of C3A spheroids. A. Time-course treatment was used to define APAP dose that increase both S-nitrosylation (SNO) and S-sulfenylation (SOH) levels with minimal effect on cell viability (assessed by ATP and protein carbonyl production). * APAP doses were calculated in mg per mg cellular protein B. The redox proteome of C3A spheroids was characterised using SNO/SOH TMT. Qualitative information was used to map SNO/SOH sites under basal conditions. Quantitative ratios of respective reporter ions were used to determine changes in SNO/SOH levels in response to APAP treatment (marked with grey boxes on the schematic spectrum). C. Data were extracted and integrated using bioinformatics approaches.

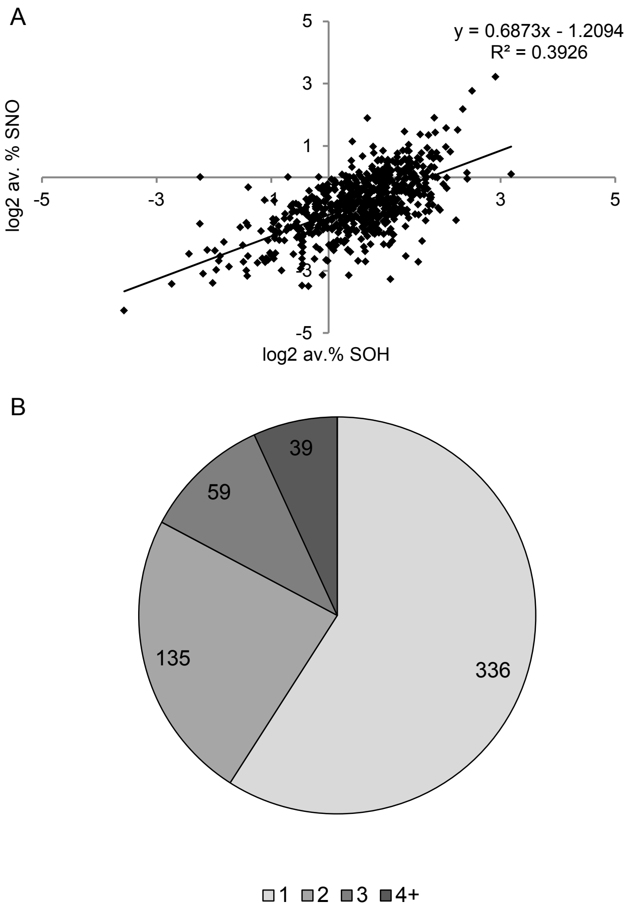

Figure S2. Characterisation of the SNO/SOH proteome of C3A spheroids.
In non-treated C3A spheroids, we have identified 887 unique, iodoTMTTM-containing peptides with SNO, SOH or both modifications. Those peptides correspond to 996 modification sites from 569 proteins (Supplementary Table S1). The majority of modification sites were shown to be able to carry both SNO and SOH modifications. The results showed that the relative abundance of SNO was on average higher than that of the corresponding SOH. **A.** Correlation of % relative abundances of SNO and SOH in identified, modified peptides. A large proportion of modified proteins contained a single SNO/SOH modified cysteine residue. **B.** Number of SNO and/or SOH modified cysteines per protein. Numbers: 1, 2, 3, 4+ correspond to the numbers of SNO/SOH modification sites identified within a sequence of a single protein. Serotransferrin (TF) and Fatty acid synthase (FASN) were the most heavily oxidised, with 13 SNO and 11 SOH different modified cysteine residues (Supplementary Table S1).

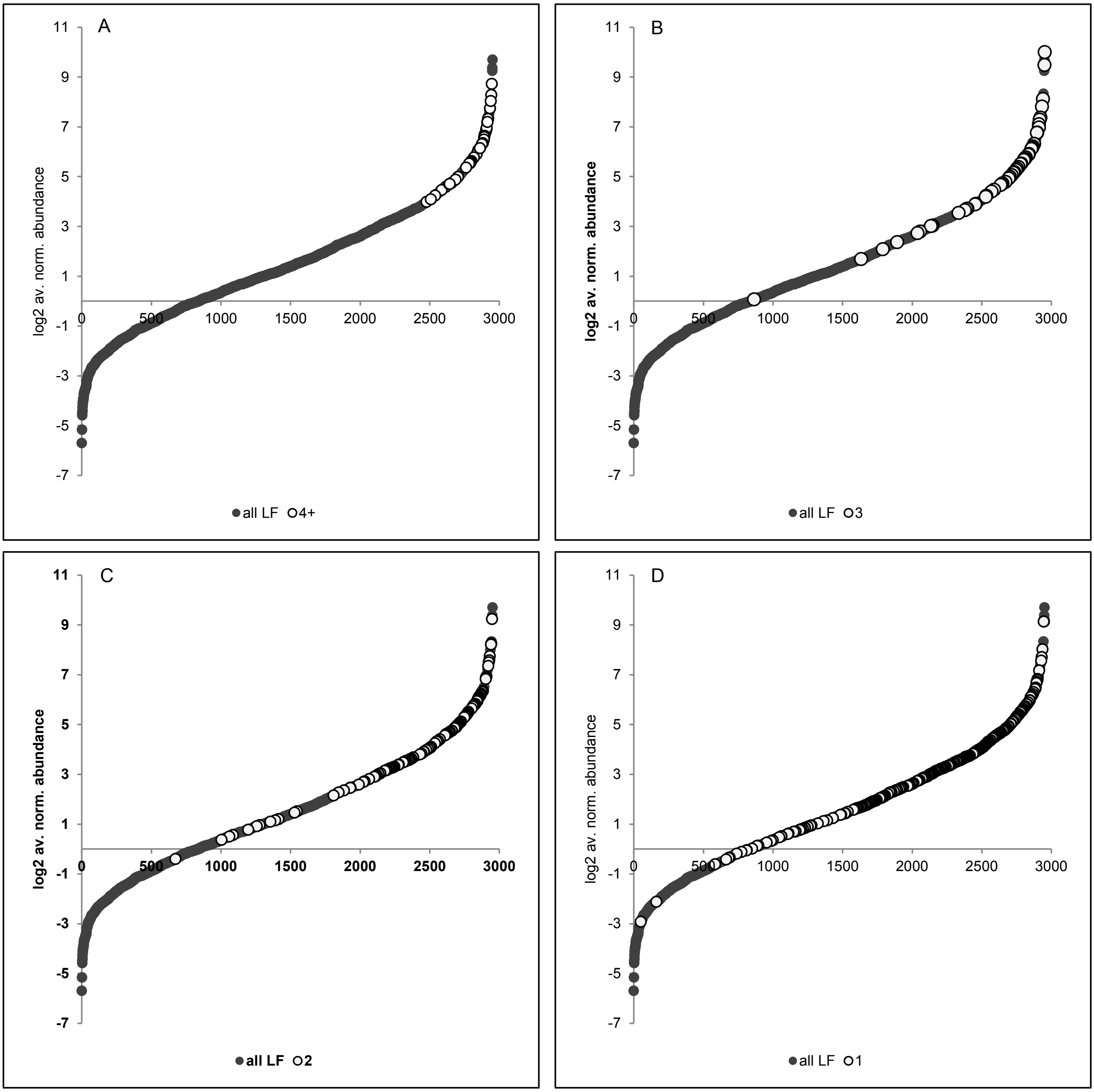

Figure S3. SNO/SOH frequency per protein is proportional to relative abundance of the protein as determined by label-free proteomics approach.
In order to understand the features of the multiply modified proteins we have plotted their relative abundance within the analysed sample (based on the label-free proteomics experiments) and aligned it with the number of modification sites identified. Although the heavily modified proteins belong to the group of the most abundant proteins within the dataset, modification sites were also identified in relatively low abundant proteins e.g. in Scaffold attachment factor B2 (SAFB2) and GPI transamidase component PIG-T (PIGT), confirming the relatively high sensitivity of SNO/SOH TMT strategy. The distribution of SNO/SOH modified proteins with different numbers of modified cysteine residues was overlaid on their respective abundances. All LF – all quantified proteins; 4+ - proteins containing more than 4 SNO/SOH sites (A); 3 - proteins containing 3 SNO/SOH sites (B); 2 - proteins containing 2 SNO/SOH sites (C); 1 – proteins containing single SNO/SOH site (D).

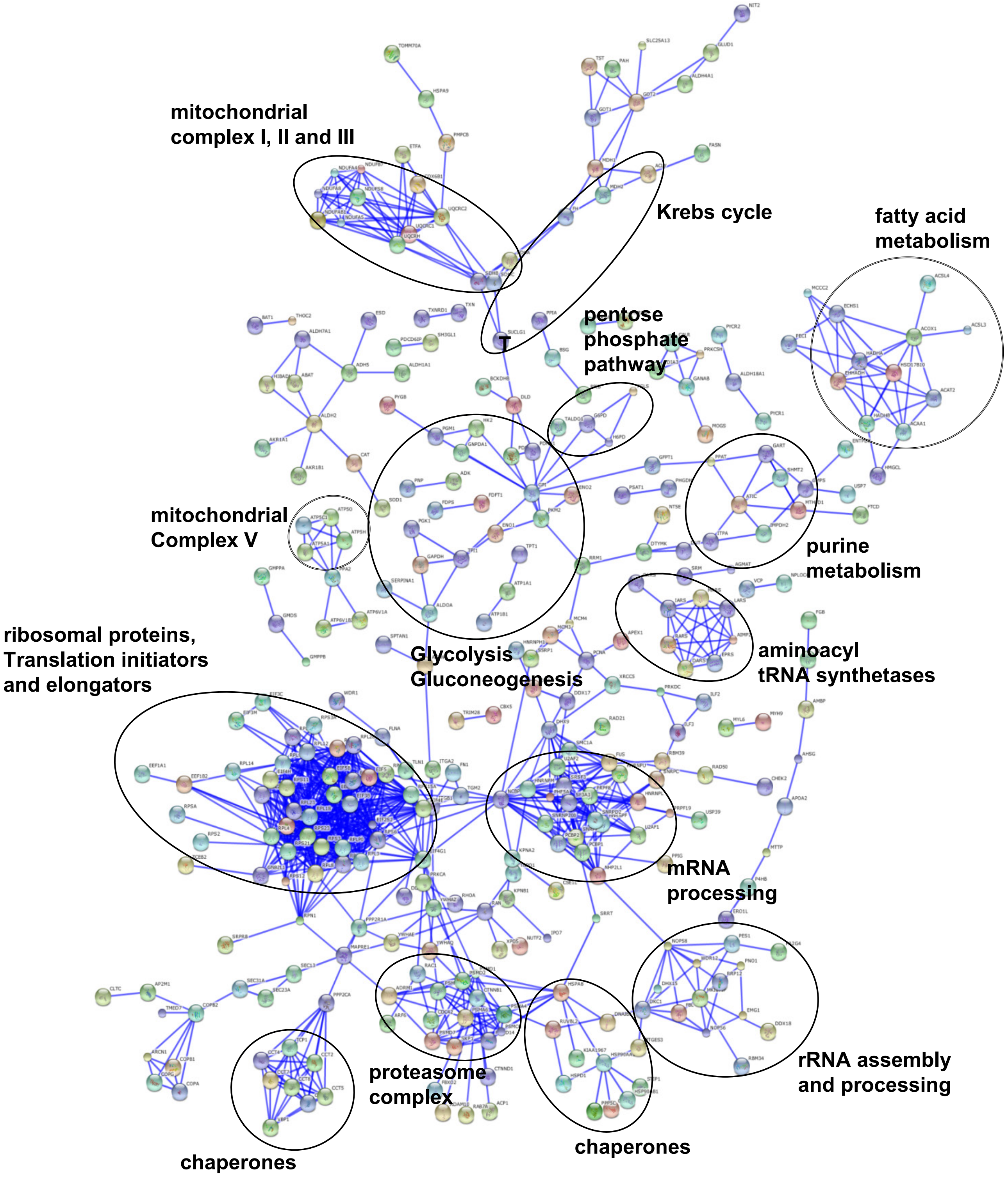

Figure S4. Molecular pathways observed in the basal SNO/SOH proteome of C3A spheroids. Functional analysis of SNO and/or SOH modified proteins under basal conditions, performed using String, v. 9.1. Grouping into functional classes was facilitated by manual verification of UniProt annotations. For details on network generation please see materials and methods section. The indicated proteins are grouped into biological processes and/or molecular functions.

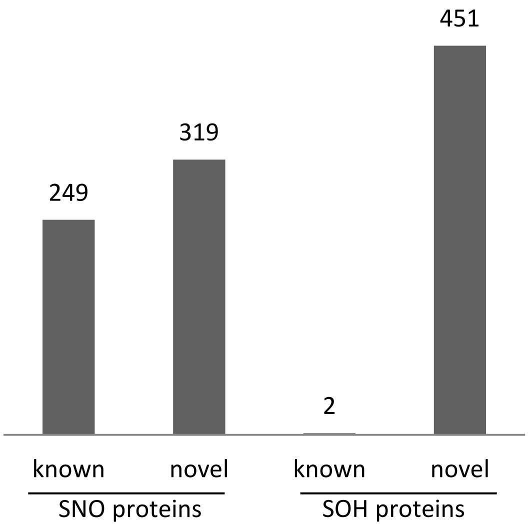

Figure S5 Number of known (RedoxDB and dbSNO) and novel SNO and SOH modified proteins identified in this study. All mapped SNO/SOH proteins together with individual modification sites were compared with the content of two databases containing curated information on cysteine oxidation, RedoxDB (Sun *et al.*, 2012) and dbSNO (Chen *et al.*, 2014). In total, we identified 249 SNO and 2 SOH proteins contained within the databases, including 213 matching SNO sites. A further 22 sites were identified in the databases as having modifications other than SNO or SOH, e.g. disulfide bonds. Additionally, we assigned sites to 38 proteins annotated as modified but which previously had no modification site specific information. For example, we mapped 6 SNO/SOH sites to D-3-phosphoglycerate dehydrogenase (PHGDH), previously only annotated as S-nitrosylated in RedoxDB. For mitochondrial aspartate aminotransferase (GOT2), a protein known as serum marker of drug-induced liver damage (Hinson, Roberts and James, 2010), we confirmed the single known SNO site and identified 4 additional SNO/SOH sites (Supplementary Table S1). Taking these known modifications into account, we identified 319 SNO and 451 SOH novel modified proteins corresponding to 477 SNO and 336 SOH sites. For example, we mapped 3 SNO/SOH and 1 SNO site of mitochondrial glutamate dehydrogenase 1 (GLUD1), whose increased plasma activity is a biomarker of mitochondrial damage (McGill *et al.*, 2012). Data for only one of these sites, C212 has been very recently deposited in dbSNO from the study of Lee, et.al. (Lee *et al.*, 2014). The above results are summarised in Supplementary Table S1.

References:

Sun, M. A., Wang, Y., Cheng, H., Zhang, Q., Ge, W., and Guo, D. (2012). RedoxDB--a curated database for experimentally verified protein oxidative modification. *Bioinformatics* **28**(19), 2551-2, 10.1093/bioinformatics/bts468.
Chen, Y. J., Lu, C. T., Su, M. G., Huang, K. Y., Ching, W. C., Yang, H. H., Liao, Y. C., Chen, Y. J., and Lee, T. Y. (2014). dbSNO 2.0: a resource for exploring structural environment, functional and disease association and regulatory network of protein S-nitrosylation. *Nucleic acids research* doi: 10.1093/nar/gku1176, 10.1093/nar/gku1176.
Hinson, J. A., Roberts, D. W., and James, L. P. (2010). Mechanisms of acetaminophen-induced liver necrosis. *Handbook of experimental pharmacology* doi: 10.1007/978-3-642-00663-0_12(196), 369-405, 10.1007/978-3-642-00663-0_12.
McGill, M. R., Sharpe, M. R., Williams, C. D., Taha, M., Curry, S. C., and Jaeschke, H. (2012). The mechanism underlying acetaminophen-induced hepatotoxicity in humans and mice involves mitochondrial damage and nuclear DNA fragmentation. *The Journal of clinical investigation* **122**(4), 1574-83, 10.1172/JCI59755.
Lee, Y. I., Giovinazzo, D., Kang, H. C., Lee, Y., Jeong, J. S., Doulias, P. T., Xie, Z., Hu, J., Ghasemi, M., Ischiropoulos, H., Qian, J., Zhu, H., Blackshaw, S., Dawson, V. L., and Dawson, T. M. (2014). Protein microarray characterization of the S-nitrosoproteome. *Molecular & cellular proteomics : MCP* **13**(1), 63-72, 10.1074/mcp.M113.032235.

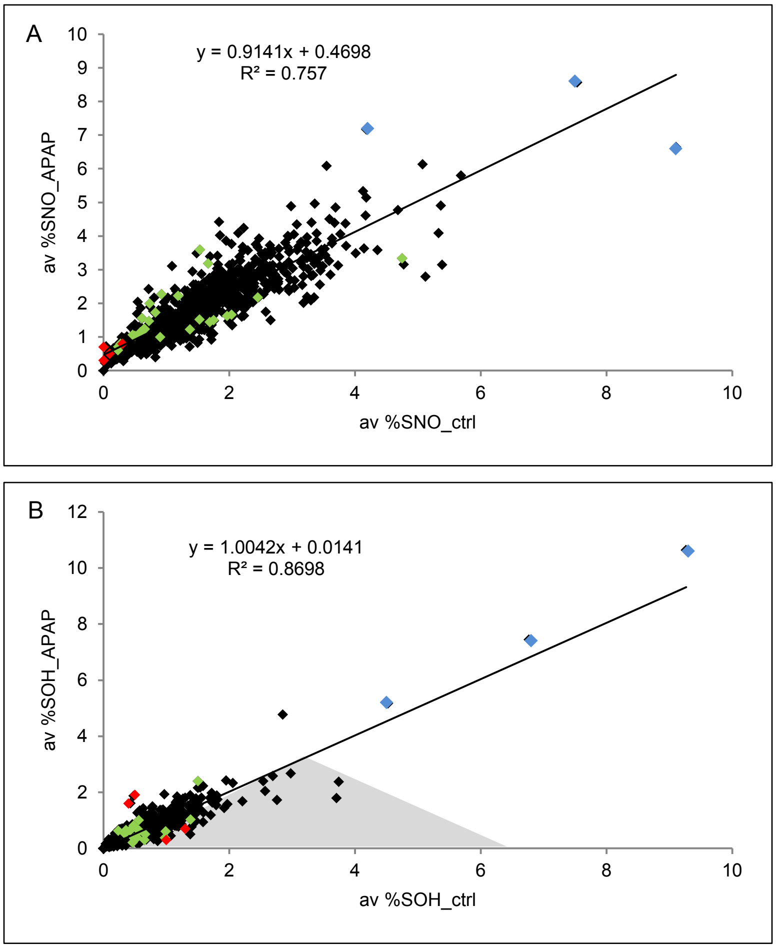

Figure S6. Relative site occupancy is complementary to relative fold change. The plots are of average, relative site occupancy in control and under APAP treatment (min. 2 replicates) for SNO (A) and SOH (B). Marked in red on A and B are relative site occupancies of 2 sigma peptides; in green – sites with +/- 1.5 fold change in SNO/SOH between control and APAP treatment; in blue – sites with highest relative SNO/SOH occupancy under APAP treatment. Marked with grey triangle in B. is the area corresponding to SOH sites of lower relative site occupancy under APAP treatment as compared to control. This potentially indicate further oxidation of sulfenic acid to sulfinic and sulfonic acid undetectable by SNO/SOH TMT.

Figure S7. Peptides carrying SNO or SOH modifications listed in Table 1. Annotated MSMS spectra and assigned ion series masses are presented.

Sequence: AVVVCPK, C5-TMT6-Cys (329.22660 Da)
Charge: +2, Monoisotopic m/z: 522.82666 Da (+1.15 mmu/+2.2 ppm), MH+: 1044.64604 Da, RT: 17.33 min,
Identified with: Mascot (v1.30); IonScore:22, Exp Value:3.6E-002, Ions matched by search engine: 4/48
Fragment match tolerance used for search: 0.05 Da

b and y ion series

#1	a^+	a^{2+}	b^+	b^{2+}	Seq.	y^+	y^{2+}	#2
1	44.04948	22.52838	72.04440	36.52584	A			7
2	143.11790	72.06259	171.11282	86.06005	V	973.60663	487.30695	6
3	242.18632	121.59680	270.18124	135.59426	V	874.53821	437.77274	5
4	341.25474	171.13101	369.24966	185.12847	V	775.46979	388.23853	4
5	773.49053	387.24890	801.48544	401.24636	C-TMT6-	676.40137	338.70432	3
6	870.54330	435.77529	898.53821	449.77274	P	244.16558	122.58643	2
7					K	147.11281	74.06004	1

annotated MSMS spectrum

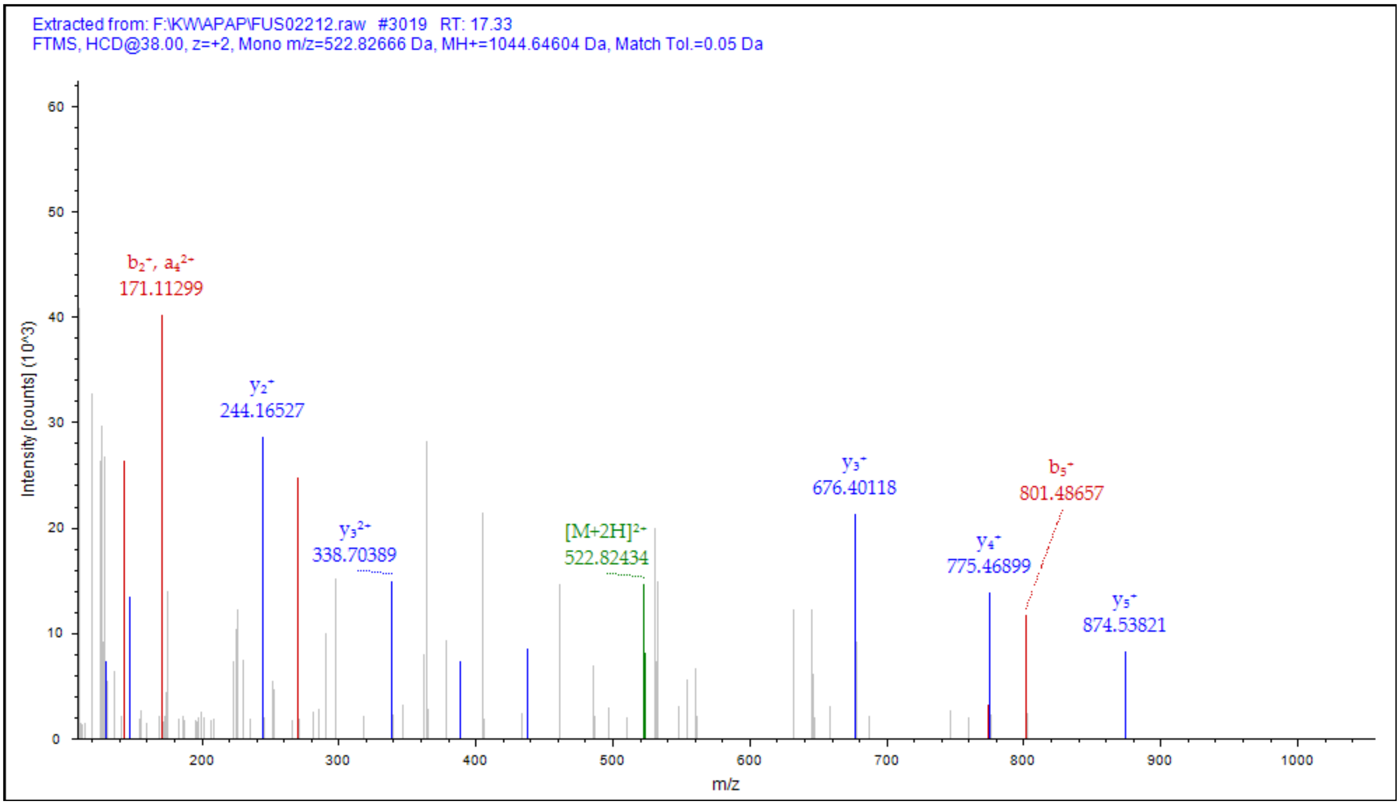

Sequence: TFCQLILDPIFK, C3-TMT6-Cys (329.22660 Da)
Charge: +3, Monoisotopic m/z: 589.67328 Da (-0.81 mmu/-1.37 ppm), MH+: 1767.00528 Da, RT: 77.33 min,
Identified with: Mascot (v1.30); IonScore:21, Exp Value:1.2E-001, Ions matched by search engine: 6/120
Fragment match tolerance used for search: 0.05 Da

b and y ion series

#1	a^+	a^{2+}	a^{3+}	b^+	b^{2+}	b^{3+}	Seq.	y^+	y^{2+}	y^{3+}	#2
1	74.06004	37.53366	25.35820	102.05496	51.53112	34.68984	T				12
2	221.12846	111.06787	74.38101	249.12338	125.06533	83.71264	F	1665.96002	833.48365	555.99152	11
3	653.36425	327.18576	218.45960	681.35916	341.18322	227.79124	C-TMT6-	1518.89160	759.94944	506.96872	10
4	781.42283	391.21505	261.14579	809.41774	405.21251	270.47743	Q	1086.65581	543.83154	362.89012	9
5	894.50690	447.75709	298.84048	922.50181	461.75454	308.17212	L	958.59723	479.80225	320.20393	8
6	1007.59097	504.29912	336.53517	1035.58588	518.29658	345.86681	I	845.51316	423.26022	282.50924	7
7	1120.67504	560.84116	374.22986	1148.66995	574.83861	383.56150	L	732.42909	366.71818	244.81455	6
8	1235.70199	618.35463	412.57218	1263.69690	632.35209	421.90382	D	619.34502	310.17615	207.11986	5
9	1332.75476	666.88102	444.92310	1360.74967	680.87847	454.25474	P	504.31807	252.66267	168.77754	4
10	1445.83883	723.42305	482.61779	1473.83374	737.42051	491.94943	I	407.26530	204.13629	136.42662	3
11	1592.90725	796.95726	531.64060	1620.90216	810.95472	540.97224	F	294.18123	147.59425	98.73193	2
12							K	147.11281	74.06004	49.70912	1

annotated MSMS spectrum

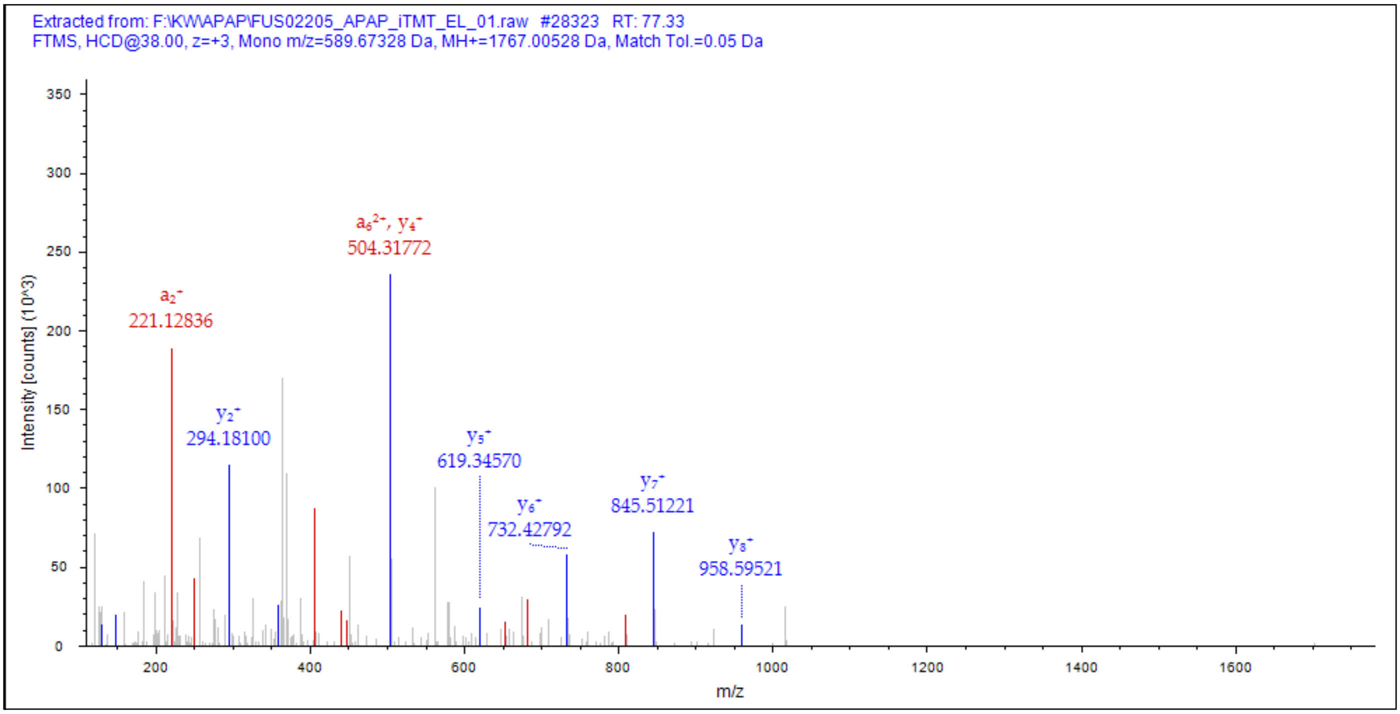

Sequence: VCMDFNIIR, C2-TMT6-Cys (329.22660 Da)
Charge: +3, Monoisotopic m/z: 480.59448 Da (-0.4 mmu/-0.84 ppm), MH+: 1439.76889 Da, RT: 53.52 min,
Identified with: Mascot (v1.30); IonScore:20, Exp Value:2.6E-001, Ions matched by search engine: 7/76
Fragment match tolerance used for search: 0.05 Da

b and y ion series

#1	a^+	a^{2+}	a^{3+}	b^+	b^{2+}	b^{3+}	Seq.	y^+	y^{2+}	y^{3+}	#2
1	72.08078	36.54403	24.69845	100.07570	50.54149	34.03008	V				9
2	504.31657	252.66192	168.77704	532.31148	266.65938	178.10868	C-TMT6-	1340.70169	670.85448	447.57208	8
3	635.35707	318.18217	212.45721	663.35198	332.17963	221.78884	M	908.46590	454.73659	303.49348	7
4	750.38402	375.69565	250.79952	778.37893	389.69310	260.13116	D	777.42540	389.21634	259.81332	6
5	897.45244	449.22986	299.82233	925.44735	463.22731	309.15397	F	662.39845	331.70286	221.47100	5
6	1011.49537	506.25132	337.83664	1039.49028	520.24878	347.16828	N	515.33003	258.16865	172.44819	4
7	1124.57944	562.79336	375.53133	1152.57435	576.79081	384.86297	I	401.28710	201.14719	134.43388	3
8	1237.66351	619.33539	413.22602	1265.65842	633.33285	422.55766	I	288.20303	144.60515	96.73919	2
9							R	175.11896	88.06312	59.04450	1

annotated MSMS spectrum

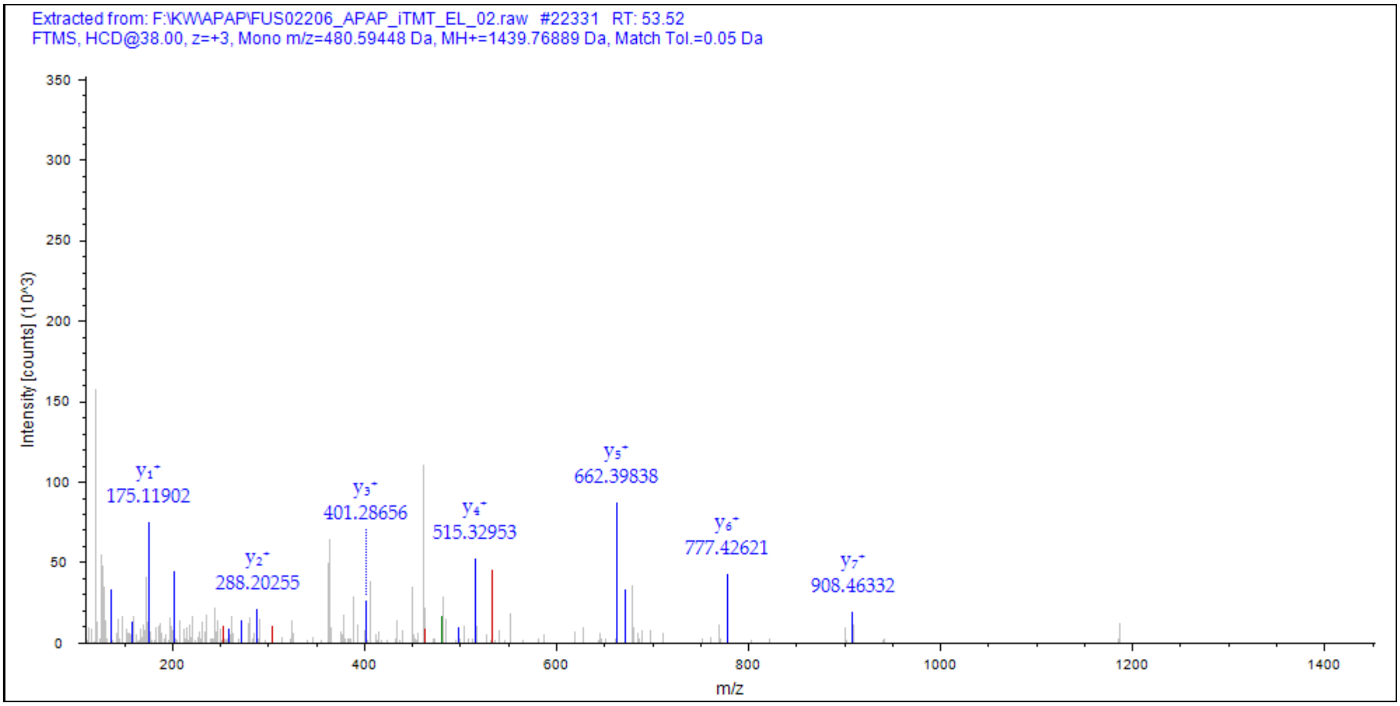

Sequence: TVYGGGCSEMLMAHAVTQLANR, C7-TMT6-Cys (329.22660 Da), M10-Oxidation (15.99492 Da)
Charge: +4, Monoisotopic m/z: 664.33185 Da (+0.11 mmu/+0.16 ppm), MH+: 2654.30556 Da, RT: 56.76 min,
Identified with: Mascot (v1.30); IonScore:29, Exp Value:5.5E-002, Ions matched by search engine: 6/184
Fragment match tolerance used for search: 0.05 Da

b and y ion series

#1	a^+	a^{2+}	a^{3+}	a^{4+}	b^+	b^{2+}	b^{3+}	b^{4+}	Seq.	y^+	y^{2+}	y^{3+}	y^{4+}	#2
1	74.06004	37.53366	25.35820	19.27047	102.05496	51.53112	34.68984	26.26920	T					22
2	173.12846	87.06787	58.38101	44.03757	201.12338	101.06533	67.71264	51.03630	V	2553.25746	1277.13237	851.75734	639.06982	21
3	336.19178	168.59953	112.73545	84.80340	364.18670	182.59699	122.06708	91.80213	Y	2454.18904	1227.59816	818.73453	614.30272	20
4	393.21325	197.11026	131.74260	99.05877	421.20817	211.10772	141.07424	106.05750	G	2291.12572	1146.06650	764.38009	573.53689	19
5	450.23472	225.62100	150.74976	113.31414	478.22964	239.61846	160.08140	120.31287	G	2234.10425	1117.55576	745.37293	559.28152	18
6	507.25619	254.13173	169.75692	127.56951	535.25111	268.12919	179.08855	134.56823	G	2177.08278	1089.04503	726.36578	545.02615	17
7	939.49198	470.24963	313.83551	235.62845	967.48689	484.24708	323.16715	242.62718	C-TMT6-	2120.06131	1060.53429	707.35862	530.77079	16
8	1026.52401	513.76564	342.84619	257.38646	1054.51892	527.76310	352.17782	264.38519	S	1687.82553	844.41640	563.28003	422.71184	15
9	1155.56661	578.28694	385.86039	289.64711	1183.56152	592.28440	395.19202	296.64584	E	1600.79350	800.90039	534.26935	400.95383	14
10	1302.60202	651.80465	434.87219	326.40596	1330.59694	665.80211	444.20383	333.40469	M-Oxidation	1471.75090	736.37909	491.25515	368.69318	13
11	1415.68609	708.34668	472.56688	354.67698	1443.68101	722.34414	481.89852	361.67571	L	1324.71548	662.86138	442.24334	331.93433	12
12	1546.72659	773.86693	516.24705	387.43711	1574.72151	787.86439	525.57869	394.43583	M	1211.63141	606.31934	404.54865	303.66331	11
13	1617.76371	809.38549	539.92609	405.19639	1645.75863	823.38295	549.25773	412.19511	A	1080.59091	540.79909	360.86849	270.90319	10
14	1754.82262	877.91495	585.61239	439.46111	1782.81754	891.91241	594.94403	446.45984	H	1009.55379	505.28053	337.18945	253.14391	9
15	1825.85974	913.43351	609.29143	457.22039	1853.85466	927.43097	618.62307	464.21912	A	872.49488	436.75108	291.50314	218.87918	8
16	1924.92816	962.96772	642.31424	481.98750	1952.92308	976.96518	651.64588	488.98623	V	801.45776	401.23252	267.82410	201.11990	7
17	2025.97584	1013.49156	675.99680	507.24942	2053.97076	1027.48902	685.32844	514.24815	T	702.38934	351.69831	234.80130	176.35279	6
18	2154.03442	1077.52085	718.68299	539.26406	2182.02934	1091.51831	728.01463	546.26279	Q	601.34166	301.17447	201.11874	151.09087	5
19	2267.11849	1134.06288	756.37768	567.53508	2295.11341	1148.06034	765.70932	574.53381	L	473.28308	237.14518	158.43254	119.07623	4
20	2338.15561	1169.58144	780.05672	585.29436	2366.15053	1183.57890	789.38836	592.29309	A	360.19901	180.60314	120.73785	90.80521	3
21	2452.19854	1226.60291	818.07103	613.80509	2480.19346	1240.60037	827.40267	620.80382	N	289.16189	145.08458	97.05881	73.04593	2
22									R	175.11896	88.06312	59.04450	44.53520	1

annotated MSMS spectrum

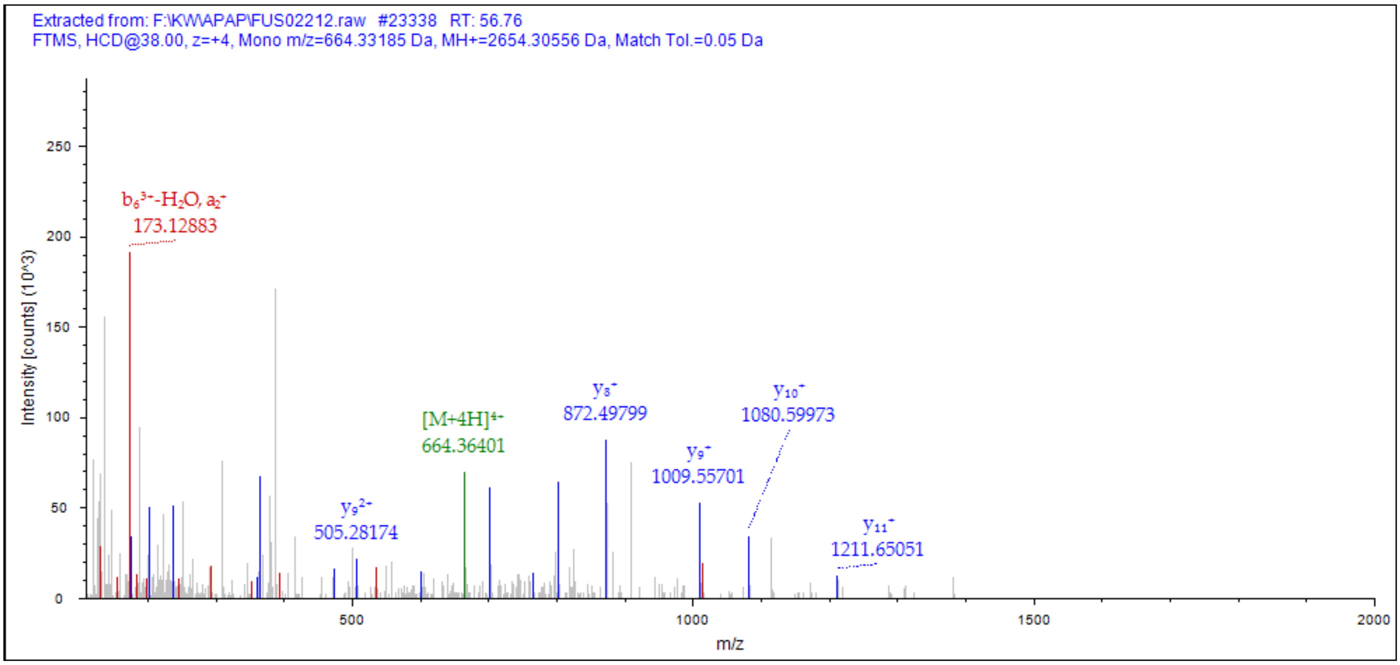

Sequence: CTLEFQVITGGHYDVDCR, C1-TMT6-Cys (329.22660 Da), C17-TMT6-Cys (329.22660 Da)
Charge: +3, Monoisotopic m/z: 905.46661 Da (+1.92 mmu/+2.12 ppm), MH+: 2714.38529 Da, RT: 49.74 min,
Identified with: Mascot (v1.30); IonScore:44, Exp Value:1.6E-003, Ions matched by search engine: 11/184
Fragment match tolerance used for search: 0.05 Da

b and y ion series

#1	a^+	a^{2+}	a^{3+}	b^+	b^{2+}	b^{3+}	Seq.	y^+	y^{2+}	y^{3+}	#2
1	405.24815	203.12771	135.75423	433.24306	217.12517	145.08587	C-TMT6-				18
2	506.29583	253.65155	169.43679	534.29074	267.64901	178.76843	T	2282.14376	1141.57552	761.38610	17
3	619.37990	310.19359	207.13148	647.37481	324.19104	216.46312	L	2181.09608	1091.05168	727.70354	16
4	748.42250	374.71489	250.14568	776.41741	388.71234	259.47732	E	2068.01201	1034.50964	690.00885	15
5	895.49092	448.24910	299.16849	923.48583	462.24655	308.50013	F	1938.96941	969.98834	646.99465	14
6	1023.54950	512.27839	341.85468	1051.54441	526.27584	351.18632	Q	1791.90099	896.45413	597.97185	13
7	1122.61792	561.81260	374.87749	1150.61283	575.81005	384.20913	V	1663.84241	832.42484	555.28565	12
8	1235.70199	618.35463	412.57218	1263.69690	632.35209	421.90382	I	1564.77399	782.89063	522.26285	11
9	1336.74967	668.87847	446.25474	1364.74458	682.87593	455.58638	T	1451.68992	726.34860	484.56816	10
10	1393.77114	697.38921	465.26190	1421.76605	711.38666	474.59353	G	1350.64224	675.82476	450.88560	9
11	1450.79261	725.89994	484.26905	1478.78752	739.89740	493.60069	G	1293.62077	647.31402	431.87844	8
12	1587.85152	794.42940	529.95536	1615.84643	808.42685	539.28699	H	1236.59930	618.80329	412.87128	7
13	1750.91484	875.96106	584.30980	1778.90975	889.95851	593.64143	Y	1099.54039	550.27383	367.18498	6
14	1865.94179	933.47453	622.65211	1893.93670	947.47199	631.98375	D	936.47707	468.74217	312.83054	5
15	1965.01021	983.00874	655.67492	1993.00512	997.00620	665.00656	V	821.45012	411.22870	274.48822	4
16	2080.03716	1040.52222	694.01724	2108.03207	1054.51967	703.34887	D	722.38170	361.69449	241.46542	3
17	2512.27294	1256.64011	838.09583	2540.26786	1270.63757	847.42747	C-TMT6-	607.35475	304.18101	203.12310	2
18							R	175.11896	88.06312	59.04450	1

annotated MSMS spectrum

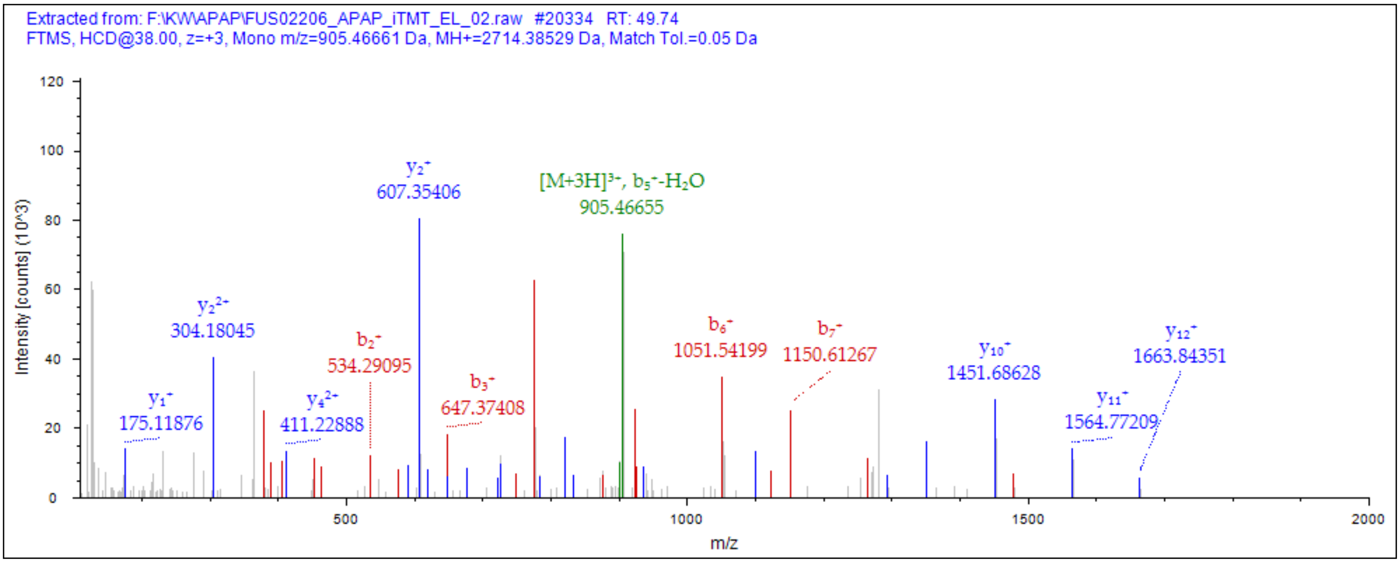

Sequence: AINCATSGVVGLVNCLR, C4-TMT6-Cys (329.22660 Da), C15-TMT6-Cys (329.22660 Da)
Charge: +4, Monoisotopic m/z: 587.83899 Da (+0.85 mmu/+1.44 ppm), MH+: 2348.33413 Da, RT: 59.75 min,
Identified with: Mascot (v1.30); IonScore:21, Exp Value:5.9E-002, Ions matched by search engine: 8/184
Fragment match tolerance used for search: 0.05 Da

b and y ion series

#1	a^{+}	a^{2+}	a^{3+}	a^{4+}	b^{+}	b^{2+}	b^{3+}	b^{4+}	Seq.	y^{+}	y^{2+}	y^{3+}	y^{4+}	#2
1	44.04948	22.52838	15.35468	11.76783	72.04440	36.52584	24.68632	18.76656	A					17
2	157.13355	79.07041	53.04937	40.03885	185.12847	93.06787	62.38101	47.03757	I	2277.29363	1139.15045	759.76939	570.07887	16
3	271.17648	136.09188	91.06368	68.54958	299.17140	150.08934	100.39532	75.54831	N	2164.20956	1082.60842	722.07470	541.80785	15
4	703.41227	352.20977	235.14227	176.60852	731.40718	366.20723	244.47391	183.60725	C-TMT6-	2050.16663	1025.58695	684.06039	513.29712	14
5	774.44939	387.72833	258.82131	194.36780	802.44430	401.72579	268.15295	201.36653	A	1617.93085	809.46906	539.98180	405.23817	13
6	875.49707	438.25217	292.50387	219.62972	903.49198	452.24963	301.83551	226.62845	T	1546.89373	773.95050	516.30276	387.47889	12
7	962.52910	481.76819	321.51455	241.38773	990.52401	495.76564	330.84619	248.38646	S	1445.84605	723.42666	482.62020	362.21697	11
8	1019.55057	510.27892	340.52171	255.64310	1047.54548	524.27638	349.85334	262.64183	G	1358.81402	679.91065	453.60952	340.45896	10
9	1118.61899	559.81313	373.54451	280.41020	1146.61390	573.81059	382.87615	287.40893	V	1301.79255	651.39991	434.60237	326.20359	9
10	1217.68741	609.34734	406.56732	305.17731	1245.68232	623.34480	415.89896	312.17604	V	1202.72413	601.86570	401.57956	301.43649	8
11	1274.70888	637.85808	425.57448	319.43268	1302.70379	651.85553	434.90611	326.43141	G	1103.65571	552.33149	368.55675	276.66938	7
12	1387.79295	694.40011	463.26917	347.70369	1415.78786	708.39757	472.60080	354.70242	L	1046.63424	523.82076	349.54960	262.41402	6
13	1486.86137	743.93432	496.29197	372.47080	1514.85628	757.93178	505.62361	379.46953	V	933.55017	467.27872	311.85491	234.14300	5
14	1600.90430	800.95579	534.30628	400.98153	1628.89921	814.95324	543.63792	407.98026	N	834.48175	417.74451	278.83210	209.37589	4
15	2033.14008	1017.07368	678.38488	509.04048	2061.13500	1031.07114	687.71652	516.03921	C-TMT6-	720.43882	360.72305	240.81779	180.86516	3
16	2146.22415	1073.61571	716.07957	537.31150	2174.21907	1087.61317	725.41121	544.31022	L	288.20303	144.60515	96.73919	72.80622	2
17									R	175.11896	88.06312	59.04450	44.53520	1

annotated MSMS spectrum

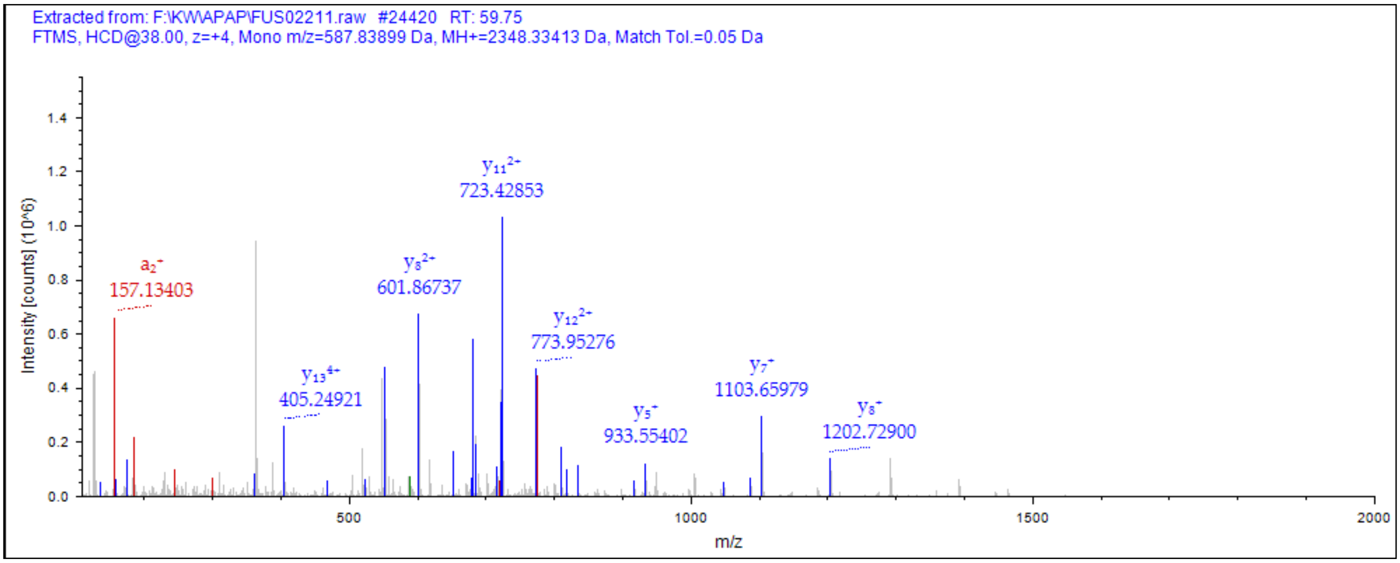

Sequence: TTCLCPNFVNTGFIK, C3-TMT6-Cys (329.22660 Da), C5-TMT6-Cys (329.22660 Da)
Charge: +4, Monoisotopic m/z: 579.82037 Da (-0.31 mmu/-0.54 ppm), MH+: 2316.25966 Da, RT: 49.31 min,
Identified with: Mascot (v1.30); IonScore:17, Exp Value:3.9E-001, Ions matched by search engine: 6/144
Fragment match tolerance used for search: 0.05 Da

b and y ion series

#1	a^{+}	a^{2+}	a^{3+}	a^{4+}	b^{+}	b^{2+}	b^{3+}	b^{4+}	Seq.	y^{+}	y^{2+}	y^{3+}	y^{4+}	#2
1	74.06004	37.53366	25.35820	19.27047	102.05496	51.53112	34.68984	26.26920	T					15
2	175.10772	88.05750	59.04076	44.53239	203.10264	102.05496	68.37240	51.53112	T	2215.21324	1108.11026	739.07593	554.55877	14
3	607.34351	304.17539	203.11935	152.59133	635.33842	318.17285	212.45099	159.59006	C-TMT6-	2114.16556	1057.58642	705.39337	529.29685	13
4	720.42758	360.71743	240.81404	180.86235	748.42249	374.71488	250.14568	187.86108	L	1681.92978	841.46853	561.31478	421.23790	12
5	1152.66336	576.83532	384.89264	288.92130	1180.65828	590.83278	394.22428	295.92003	C-TMT6-	1568.84571	784.92649	523.62009	392.96688	11
6	1249.71613	625.36170	417.24356	313.18449	1277.71105	639.35916	426.57520	320.18322	P	1136.60992	568.80860	379.54149	284.90794	10
7	1363.75906	682.38317	455.25787	341.69522	1391.75398	696.38063	464.58951	348.69395	N	1039.55715	520.28221	347.19057	260.64475	9
8	1510.82748	755.91738	504.28068	378.46233	1538.82240	769.91484	513.61232	385.46106	F	925.51422	463.26075	309.17626	232.13401	8
9	1609.89590	805.45159	537.30349	403.22943	1637.89082	819.44905	546.63512	410.22816	V	778.44580	389.72654	260.15345	195.36691	7
10	1723.93883	862.47305	575.31780	431.74017	1751.93375	876.47051	584.64943	438.73889	N	679.37738	340.19233	227.13064	170.59980	6
11	1824.98651	912.99689	609.00036	457.00209	1852.98143	926.99435	618.33199	464.00081	T	565.33445	283.17086	189.11633	142.08907	5
12	1882.00798	941.50763	628.00751	471.25745	1910.00290	955.50509	637.33915	478.25618	G	464.28677	232.64702	155.43377	116.82715	4
13	2029.07640	1015.04184	677.03032	508.02456	2057.07132	1029.03930	686.36196	515.02329	F	407.26530	204.13629	136.42662	102.57178	3
14	2142.16047	1071.58387	714.72501	536.29558	2170.15539	1085.58133	724.05665	543.29430	I	260.19688	130.60208	87.40381	65.80468	2
15									K	147.11281	74.06004	49.70912	37.53366	1

annotated MSMS spectrum

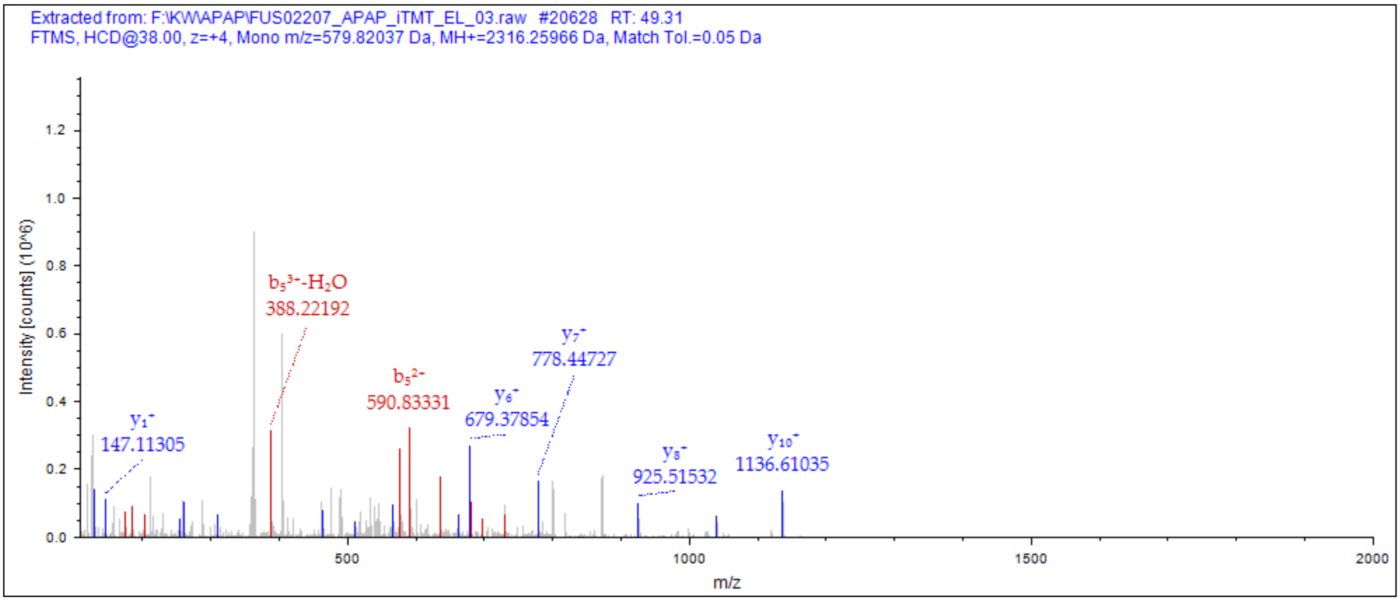

Sequence: IVSVGDDQEIHIYDCPI, C15-TMT6-Cys (329.22660 Da)
Charge: +3, Monoisotopic m/z: 749.05054 Da (-0.07 mmu/-0.1 ppm), MH+: 2245.13706 Da, RT: 63.39 min,
Identified with: Mascot (v1.30); IonScore:25, Exp Value:1.1E-001, Ions matched by search engine: 5/146
Fragment match tolerance used for search: 0.05 Da

b and y ion series

#1	a⁺	a²⁺	a³⁺	b⁺	b²⁺	b³⁺	Seq.	y⁺	y²⁺	y³⁺	#2
1	86.09643	43.55185	29.37033	114.09135	57.54931	38.70197	I				17
2	185.16485	93.08606	62.39314	213.15977	107.08352	71.72477	V	2132.05321	1066.53024	711.35592	16
3	272.19688	136.60208	91.40381	300.19180	150.59954	100.73545	S	2032.98479	1016.99603	678.33311	15
4	371.26530	186.13629	124.42662	399.26022	200.13375	133.75826	V	1945.95276	973.48002	649.32244	14
5	428.28677	214.64702	143.43378	456.28169	228.64448	152.76541	G	1846.88434	923.94581	616.29963	13
6	543.31372	272.16050	181.77609	571.30864	286.15796	191.10773	D	1789.86287	895.43507	597.29247	12
7	658.34067	329.67397	220.11841	686.33559	343.67143	229.45005	D	1674.83592	837.92160	558.95016	11
8	786.39925	393.70326	262.80460	814.39417	407.70072	272.13624	Q	1559.80897	780.40812	520.60784	10
9	915.44185	458.22456	305.81880	943.43677	472.22202	315.15044	E	1431.75039	716.37883	477.92165	9
10	1028.52592	514.76660	343.51349	1056.52084	528.76406	352.84513	I	1302.70779	651.85753	434.90745	8
11	1165.58483	583.29605	389.19980	1193.57975	597.29351	398.53143	H	1189.62372	595.31550	397.21276	7
12	1278.66890	639.83809	426.89449	1306.66382	653.83555	436.22612	I	1052.56481	526.78604	351.52645	6
13	1441.73222	721.36975	481.24893	1469.72714	735.36721	490.58056	Y	939.48074	470.24401	313.83176	5
14	1556.75917	778.88322	519.59124	1584.75409	792.88068	528.92288	D	776.41742	388.71235	259.47732	4
15	1988.99496	995.00112	663.66984	2016.98987	1008.99857	673.00147	C-TMT6-	661.39047	331.19887	221.13501	3
16	2086.04773	1043.52750	696.02076	2114.04264	1057.52496	705.35240	P	229.15468	115.08098	77.05641	2
17							I	132.10191	66.55459	44.70549	1

annotated MSMS spectrum

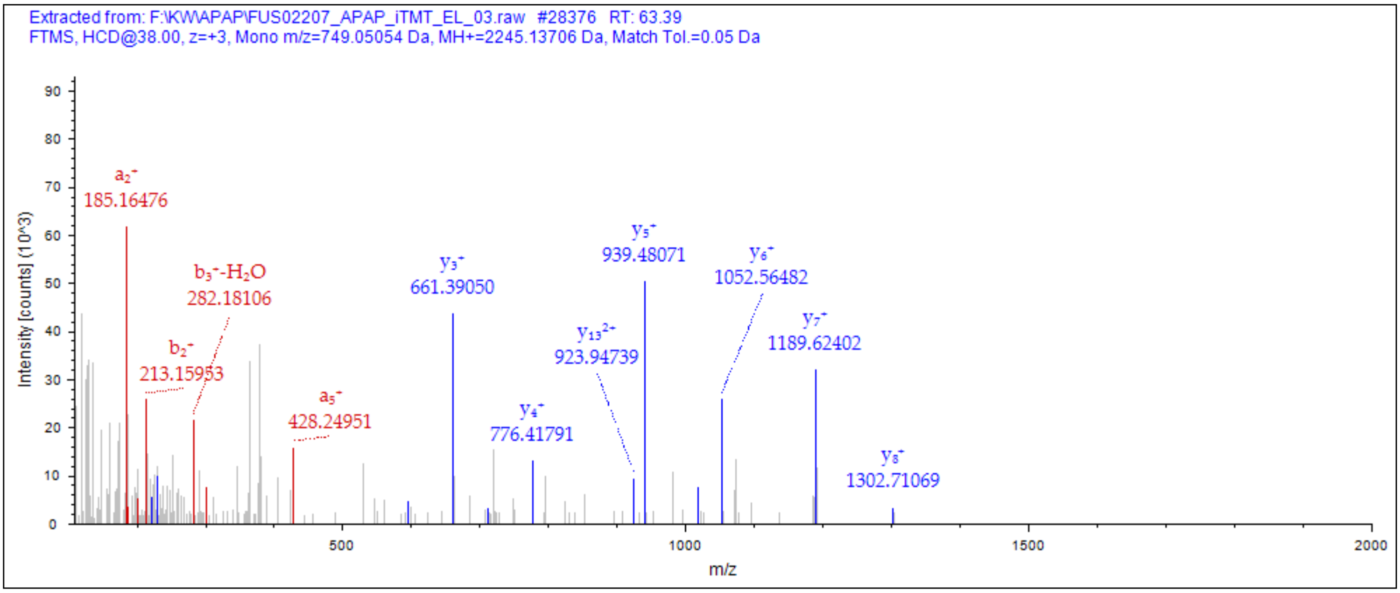

Sequence: CILPFDK, C1-TMT6-Cys (329.22660 Da)
Charge: +3, Monoisotopic m/z: 388.89355 Da (+0.41 mmu/+1.06 ppm), MH+: 1164.66611 Da, RT: 45.73 min,
Identified with: Mascot (v1.30); IonScore:22, Exp Value:1.2E-001, Ions matched by search engine: 7/48
Fragment match tolerance used for search: 0.05 Da

b and y ion series

#1	a^{+}	a^{2+}	a^{3+}	b^{+}	b^{2+}	b^{3+}	Seq.	y^{+}	y^{2+}	y^{3+}	#2
1	405.24815	203.12771	135.75423	433.24306	217.12517	145.08587	C-TMT6-				7
2	518.33222	259.66975	173.44892	546.32713	273.66720	182.78056	I	732.42909	366.71818	244.81455	6
3	631.41629	316.21178	211.14361	659.41120	330.20924	220.47525	L	619.34502	310.17615	207.11986	5
4	728.46906	364.73817	243.49454	756.46397	378.73562	252.82617	P	506.26095	253.63411	169.42517	4
5	875.53748	438.27238	292.51734	903.53239	452.26983	301.84898	F	409.20818	205.10773	137.07424	3
6	990.56443	495.78585	330.85966	1018.55934	509.78331	340.19130	D	262.13976	131.57352	88.05144	2
7							K	147.11281	74.06004	49.70912	1

annotated MSMS spectrum

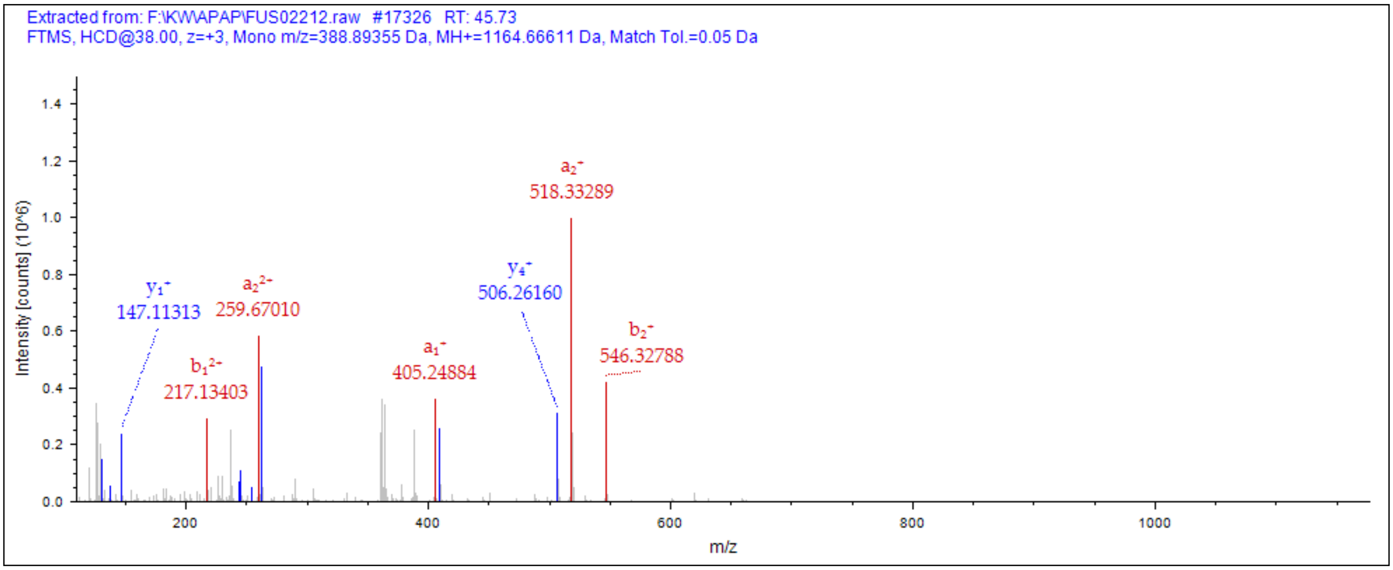

Sequence: STYSPLPDDYNCNVELALTSDGR, C12-TMT6-Cys (329.22660 Da)
Charge: +3, Monoisotopic m/z: 953.79352 Da (-0.3 mmu/-0.32 ppm), MH+: 2859.36600 Da, RT: 66.60 min,
Identified with: Mascot (v1.30); IonScore:40, Exp Value:3.8E-003, Ions matched by search engine: 18/224
Fragment match tolerance used for search: 0.05 Da

b and y ion series

#1	a^+	a^{2+}	a^{3+}	b^+	b^{2+}	b^{3+}	Seq.	y^+	y^{2+}	y^{3+}	#2
1	60.04439	30.52583	20.68632	88.03931	44.52329	30.01795	S				23
2	161.09207	81.04967	54.36888	189.08699	95.04713	63.70051	T	2772.33488	1386.67108	924.78314	22
3	324.15539	162.58133	108.72332	352.15031	176.57879	118.05495	Y	2671.28720	1336.14724	891.10058	21
4	411.18742	206.09735	137.73399	439.18234	220.09481	147.06563	S	2508.22388	1254.61558	836.74614	20
5	508.24019	254.62373	170.08492	536.23511	268.62119	179.41655	P	2421.19185	1211.09956	807.73547	19
6	621.32426	311.16577	207.77961	649.31918	325.16323	217.11124	L	2324.13908	1162.57318	775.38454	18
7	718.37703	359.69215	240.13053	746.37195	373.68961	249.46217	P	2211.05501	1106.03114	737.68985	17
8	833.40398	417.20563	278.47285	861.39890	431.20309	287.80448	D	2114.00224	1057.50476	705.33893	16
9	948.43093	474.71910	316.81516	976.42585	488.71656	326.14680	D	1998.97529	999.99128	666.99661	15
10	1111.49425	556.25076	371.16960	1139.48917	570.24822	380.50124	Y	1883.94834	942.47781	628.65430	14
11	1225.53718	613.27223	409.18391	1253.53210	627.26969	418.51555	N	1720.88502	860.94615	574.29986	13
12	1657.77297	829.39012	553.26251	1685.76788	843.38758	562.59414	C-TMT6-	1606.84209	803.92468	536.28555	12
13	1771.81590	886.41159	591.27682	1799.81081	900.40904	600.60845	N	1174.60630	587.80679	392.20695	11
14	1870.88432	935.94580	624.29962	1898.87923	949.94325	633.63126	V	1060.56337	530.78532	354.19264	10
15	1999.92692	1000.46710	667.31382	2027.92183	1014.46455	676.64546	E	961.49495	481.25111	321.16983	9
16	2113.01099	1057.00913	705.00851	2141.00590	1071.00659	714.34015	L	832.45235	416.72981	278.15563	8
17	2184.04811	1092.52769	728.68755	2212.04302	1106.52515	738.01919	A	719.36828	360.18778	240.46094	7
18	2297.13218	1149.06973	766.38224	2325.12709	1163.06718	775.71388	L	648.33116	324.66922	216.78190	6
19	2398.17986	1199.59357	800.06480	2426.17477	1213.59102	809.39644	T	535.24709	268.12718	179.08721	5
20	2485.21189	1243.10958	829.07548	2513.20680	1257.10704	838.40712	S	434.19941	217.60334	145.40465	4
21	2600.23884	1300.62306	867.41780	2628.23375	1314.62051	876.74943	D	347.16738	174.08733	116.39398	3
22	2657.26031	1329.13379	886.42495	2685.25522	1343.13125	895.75659	G	232.14043	116.57385	78.05166	2
23							R	175.11896	88.06312	59.04450	1

annotated MSMS spectrum

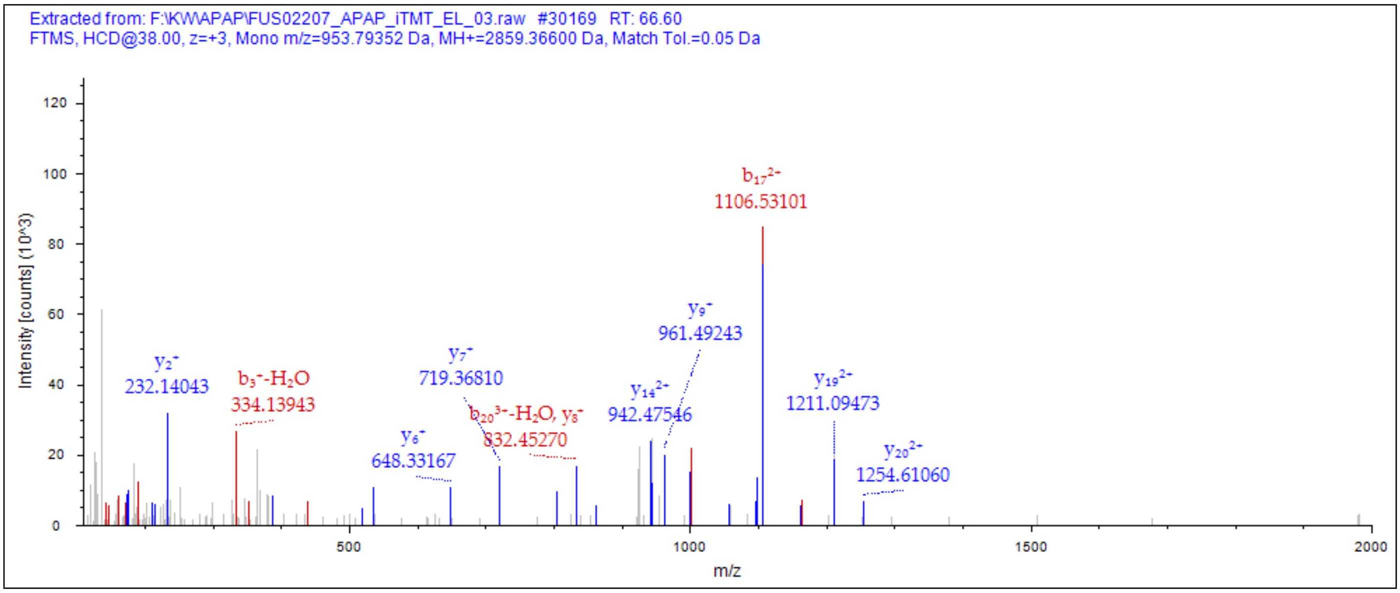

Sequence: TALLDISCVK, C8-TMT6-Cys (329.22660 Da)
Charge: +2, Monoisotopic m/z: 696.41107 Da (+0.93 mmu/+1.34 ppm), MH+: 1391.81487 Da, RT: 46.65 min,
Identified with: Mascot (v1.30); IonScore:53, Exp Value:5.3E-005, Ions matched by search engine: 6/72
Fragment match tolerance used for search: 0.05 Da

b and y ion series

#1	a^{+}	a^{2+}	b^{+}	b^{2+}	Seq.	y^{+}	y^{2+}	#2
1	74.06004	37.53366	102.05496	51.53112	T			10
2	145.09716	73.05222	173.09208	87.04968	A	1290.76533	645.88630	9
3	258.18123	129.59425	286.17615	143.59171	L	1219.72821	610.36774	8
4	371.26530	186.13629	399.26022	200.13375	L	1106.64414	553.82571	7
5	486.29225	243.64976	514.28717	257.64722	D	993.56007	497.28367	6
6	599.37632	300.19180	627.37124	314.18926	I	878.53312	439.77020	5
7	686.40835	343.70781	714.40327	357.70527	S	765.44905	383.22816	4
8	1118.64414	559.82571	1146.63905	573.82316	C-TMT6-	678.41702	339.71215	3
9	1217.71256	609.35992	1245.70747	623.35737	V	246.18123	123.59425	2
10					K	147.11281	74.06004	1

annotated MSMS spectrum

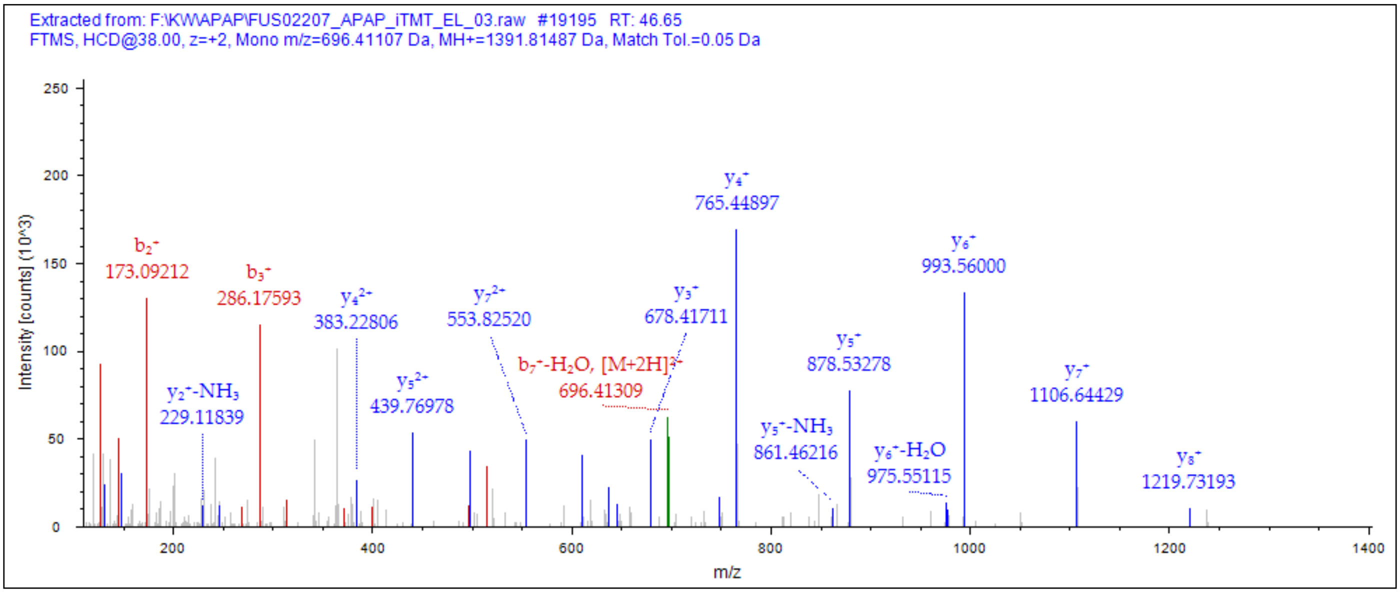

Sequence: LLLCGGAPLSATTQR, C4-TMT6-Cys (329.22660 Da)
Charge: +3, Monoisotopic m/z: 610.68768 Da (+0.52 mmu/+0.85 ppm), MH+: 1830.04850 Da, RT: 46.16 min,
Identified with: Mascot (v1.30); IonScore:21, Exp Value:7.4E-002, Ions matched by search engine: 9/116
Fragment match tolerance used for search: 0.05 Da

b and y ion series

#1	a^+	a^{2+}	a^{3+}	b^+	b^{2+}	b^{3+}	Seq.	y^+	y^{2+}	y^{3+}	#2
1	86.09643	43.55185	29.37033	114.09135	57.54931	38.70197	L				15
2	199.18050	100.09389	67.06502	227.17542	114.09135	76.39666	L	1716.96288	858.98508	572.99248	14
3	312.26457	156.63592	104.75971	340.25949	170.63338	114.09135	L	1603.87881	802.44304	535.29779	13
4	744.50036	372.75382	248.83830	772.49527	386.75127	258.16994	C-TMT6-	1490.79474	745.90101	497.60310	12
5	801.52183	401.26455	267.84546	829.51674	415.26201	277.17710	G	1058.55895	529.78311	353.52450	11
6	858.54330	429.77529	286.85262	886.53821	443.77274	296.18425	G	1001.53748	501.27238	334.51734	10
7	929.58042	465.29385	310.53166	957.57533	479.29130	319.86329	A	944.51601	472.76164	315.51019	9
8	1026.63319	513.82023	342.88258	1054.62810	527.81769	352.21422	P	873.47889	437.24308	291.83115	8
9	1139.71726	570.36227	380.57727	1167.71217	584.35972	389.90891	L	776.42612	388.71670	259.48022	7
10	1226.74929	613.87828	409.58795	1254.74420	627.87574	418.91958	S	663.34205	332.17466	221.78553	6
11	1297.78641	649.39684	433.26699	1325.78132	663.39430	442.59862	A	576.31002	288.65865	192.77486	5
12	1398.83409	699.92068	466.94955	1426.82900	713.91814	476.28118	T	505.27290	253.14009	169.09582	4
13	1499.88177	750.44452	500.63211	1527.87668	764.44198	509.96374	T	404.22522	202.61625	135.41326	3
14	1627.94035	814.47381	543.31830	1655.93526	828.47127	552.64994	Q	303.17754	152.09241	101.73070	2
15							R	175.11896	88.06312	59.04450	1

annotated MSMS spectrum

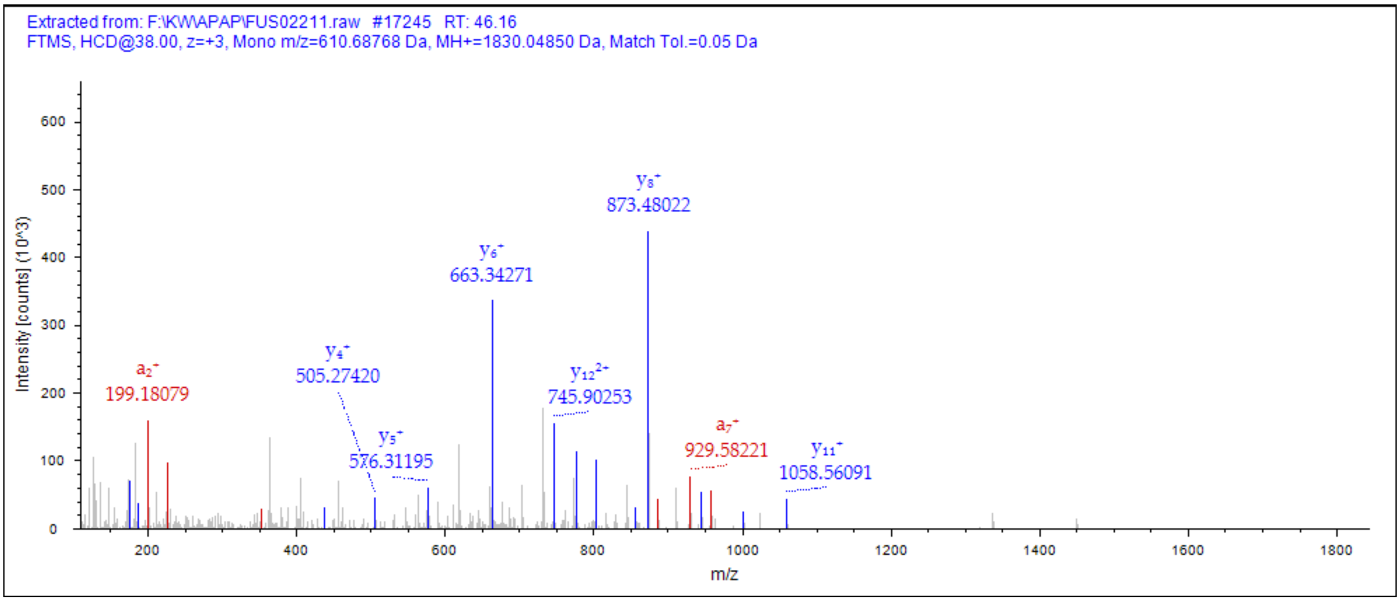

Sequence: ICSWNVDGLR, C2-TMT6-Cys (329.22660 Da)
Charge: +3, Monoisotopic m/z: 497.93692 Da (+0.73 mmu/+1.48 ppm), MH+: 1491.79621 Da, RT: 44.66 min,
Identified with: Mascot (v1.30); IonScore:24, Exp Value:1.2E-001, Ions matched by search engine: 7/92
Fragment match tolerance used for search: 0.05 Da

b and y ion series

#1	a⁺	a²⁺	a³⁺	b⁺	b²⁺	b³⁺	Seq.	y⁺	y²⁺	y³⁺	#2
1	86.09643	43.55185	29.37033	114.09135	57.54931	38.70197	I				10
2	518.33222	259.66975	173.44892	546.32713	273.66720	182.78056	C-TMT6-	1378.70994	689.85861	460.24150	9
3	605.36425	303.18576	202.45960	633.35916	317.18322	211.79124	S	946.47415	473.74071	316.16290	8
4	791.44357	396.22542	264.48604	819.43848	410.22288	273.81768	W	859.44212	430.22470	287.15222	7
5	905.48650	453.24689	302.50035	933.48141	467.24434	311.83199	N	673.36280	337.18504	225.12578	6
6	1004.55492	502.78110	335.52316	1032.54983	516.77855	344.85479	V	559.31987	280.16357	187.11147	5
7	1119.58187	560.29457	373.86547	1147.57678	574.29203	383.19711	D	460.25145	230.62936	154.08867	4
8	1176.60334	588.80531	392.87263	1204.59825	602.80276	402.20427	G	345.22450	173.11589	115.74635	3
9	1289.68741	645.34734	430.56732	1317.68232	659.34480	439.89896	L	288.20303	144.60515	96.73919	2
10							R	175.11896	88.06312	59.04450	1

annotated MSMS spectrum

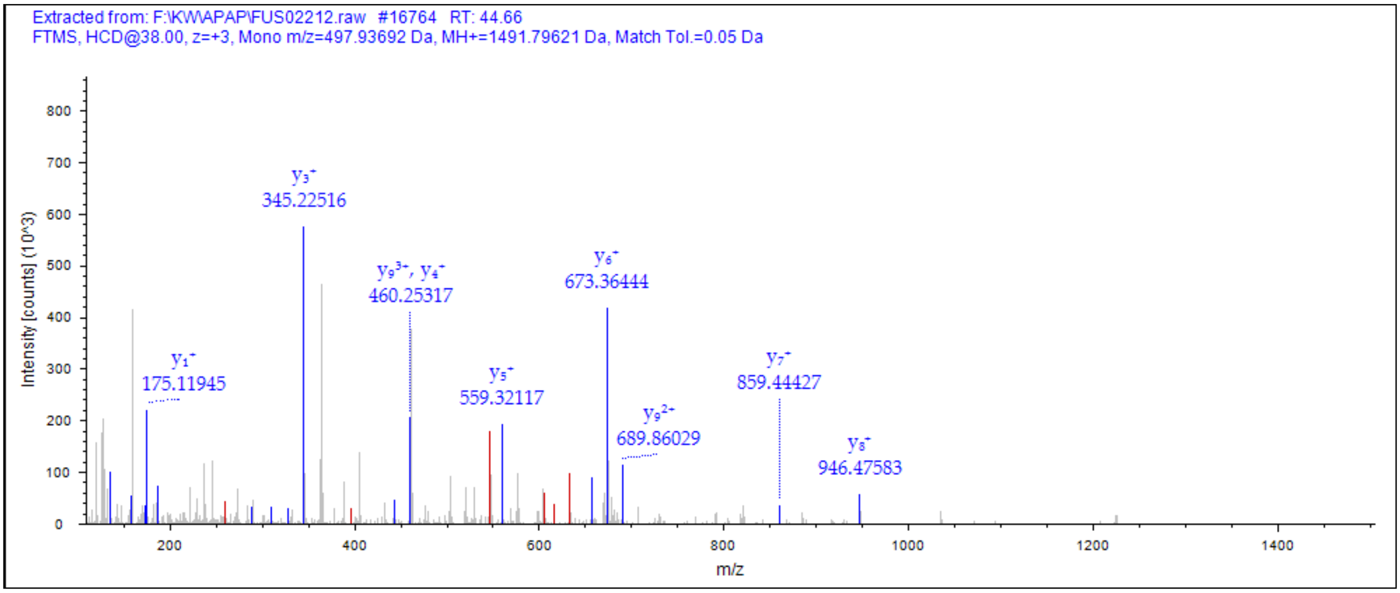

Sequence: LQGEGLSVAGIVCHVGK, C13-TMT6-Cys (329.22660 Da)
Charge: +4, Monoisotopic m/z: 499.78476 Da (-1 mmu/-1.99 ppm), MH+: 1996.11721 Da, RT: 47.20 min,
Identified with: Mascot (v1.30); IonScore:16, Exp Value:3.7E-001, Ions matched by search engine: 4/188
Fragment match tolerance used for search: 0.05 Da

b and y ion series

#1	a^+	a^{2+}	a^{3+}	a^{4+}	b^+	b^{2+}	b^{3+}	b^{4+}	Seq.	y^+	y^{2+}	y^{3+}	y^{4+}	#2
1	86.09643	43.55185	29.37033	22.27957	114.09135	57.54931	38.70197	29.27829	L					17
2	214.15501	107.58114	72.05652	54.29421	242.14993	121.57860	81.38816	61.29294	Q	1883.03712	942.02220	628.35056	471.51474	16
3	271.17648	136.09188	91.06368	68.54958	299.17140	150.08934	100.39532	75.54831	G	1754.97854	877.99291	585.66436	439.50009	15
4	400.21908	200.61318	134.07788	100.81023	428.21400	214.61064	143.40952	107.80896	E	1697.95707	849.48217	566.65721	425.24472	14
5	457.24055	229.12391	153.08504	115.06560	485.23547	243.12137	162.41667	122.06432	G	1568.91447	784.96087	523.64301	392.98407	13
6	570.32462	285.66595	190.77973	143.33661	598.31954	299.66341	200.11136	150.33534	L	1511.89300	756.45014	504.63585	378.72871	12
7	657.35665	329.18196	219.79040	165.09462	685.35157	343.17942	229.12204	172.09335	S	1398.80893	699.90810	466.94116	350.45769	11
8	756.42507	378.71617	252.81321	189.86173	784.41999	392.71363	262.14485	196.86045	V	1311.77690	656.39209	437.93048	328.69968	10
9	827.46219	414.23473	276.49225	207.62101	855.45711	428.23219	285.82389	214.61973	A	1212.70848	606.85788	404.90768	303.93258	9
10	884.48366	442.74547	295.49941	221.87637	912.47858	456.74293	304.83104	228.87510	G	1141.67136	571.33932	381.22864	286.17330	8
11	997.56773	499.28750	333.19410	250.14739	1025.56265	513.28496	342.52573	257.14612	I	1084.64989	542.82858	362.22148	271.91793	7
12	1096.63615	548.82171	366.21690	274.91450	1124.63107	562.81917	375.54854	281.91322	V	971.56582	486.28655	324.52679	243.64691	6
13	1528.87194	764.93961	510.29550	382.97344	1556.86685	778.93706	519.62713	389.97217	C-TMT6-	872.49740	436.75234	291.50398	218.87981	5
14	1665.93085	833.46906	555.98180	417.23817	1693.92576	847.46652	565.31344	424.23690	H	440.26161	220.63444	147.42539	110.82086	4
15	1764.99927	883.00327	589.00461	442.00527	1792.99418	897.00073	598.33624	449.00400	V	303.20270	152.10499	101.73908	76.55613	3
16	1822.02074	911.51401	608.01176	456.26064	1850.01565	925.51146	617.34340	463.25937	G	204.13428	102.57078	68.71628	51.78903	2
17									K	147.11281	74.06004	49.70912	37.53366	1

annotated MSMS spectrum

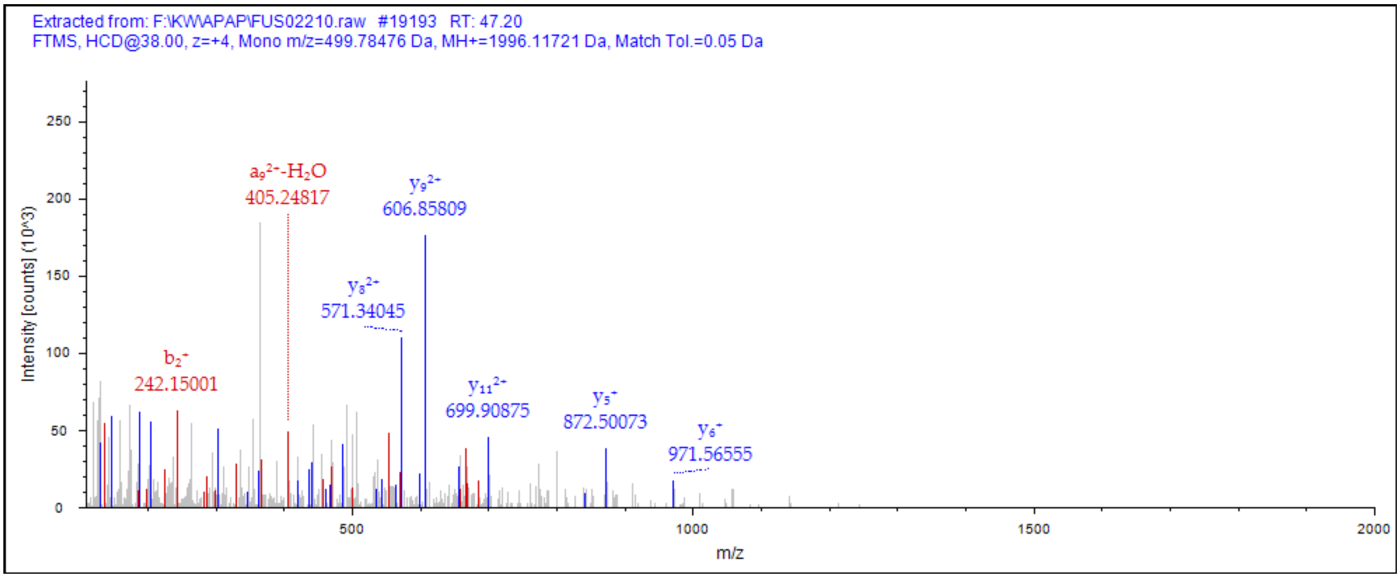

Sequence: TNMLLQLDGSTPICEDIGR, C14-TMT6-Cys (329.22660 Da)
Charge: +3, Monoisotopic m/z: 802.41852 Da (+1.43 mmu/+1.79 ppm), MH+: 2405.24100 Da, RT: 70.27 min,
Identified with: Mascot (v1.30); IonScore:38, Exp Value:6.6E-003, Ions matched by search engine: 17/212
Fragment match tolerance used for search: 0.05 Da

b and y ion series

#1	a^+	a^{2+}	a^{3+}	b^+	b^{2+}	b^{3+}	Seq.	y^+	y^{2+}	y^{3+}	#2
1	74.06004	37.53366	25.35820	102.05496	51.53112	34.68984	T				19
2	188.10297	94.55512	63.37251	216.09789	108.55258	72.70415	N	2304.18903	1152.59815	768.73453	18
3	319.14347	160.07537	107.05268	347.13839	174.07283	116.38431	M	2190.14610	1095.57669	730.72022	17
4	432.22754	216.61741	144.74737	460.22246	230.61487	154.07900	L	2059.10560	1030.05644	687.04005	16
5	545.31161	273.15944	182.44206	573.30653	287.15690	191.77369	L	1946.02153	973.51440	649.34536	15
6	673.37019	337.18873	225.12825	701.36511	351.18619	234.45989	Q	1832.93746	916.97237	611.65067	14
7	786.45426	393.73077	262.82294	814.44918	407.72823	272.15458	L	1704.87888	852.94308	568.96448	13
8	901.48121	451.24424	301.16526	929.47613	465.24170	310.49689	D	1591.79481	796.40104	531.26979	12
9	958.50268	479.75498	320.17241	986.49760	493.75244	329.50405	G	1476.76786	738.88757	492.92747	11
10	1045.53471	523.27099	349.18309	1073.52963	537.26845	358.51473	S	1419.74639	710.37683	473.92031	10
11	1146.58239	573.79483	382.86565	1174.57731	587.79229	392.19729	T	1332.71436	666.86082	444.90964	9
12	1243.63516	622.32122	415.21657	1271.63008	636.31868	424.54821	P	1231.66668	616.33698	411.22708	8
13	1356.71923	678.86325	452.91126	1384.71415	692.86071	462.24290	I	1134.61391	567.81059	378.87615	7
14	1788.95502	894.98115	596.98986	1816.94993	908.97860	606.32149	C-TMT6-	1021.52984	511.26856	341.18146	6
15	1917.99762	959.50245	640.00406	1945.99253	973.49990	649.33569	E	589.29405	295.15066	197.10287	5
16	2033.02457	1017.01592	678.34637	2061.01948	1031.01338	687.67801	D	460.25145	230.62936	154.08867	4
17	2146.10864	1073.55796	716.04106	2174.10355	1087.55541	725.37270	I	345.22450	173.11589	115.74635	3
18	2203.13011	1102.06869	735.04822	2231.12502	1116.06615	744.37986	G	232.14043	116.57385	78.05166	2
19							R	175.11896	88.06312	59.04450	1

annotated MSMS spectrum

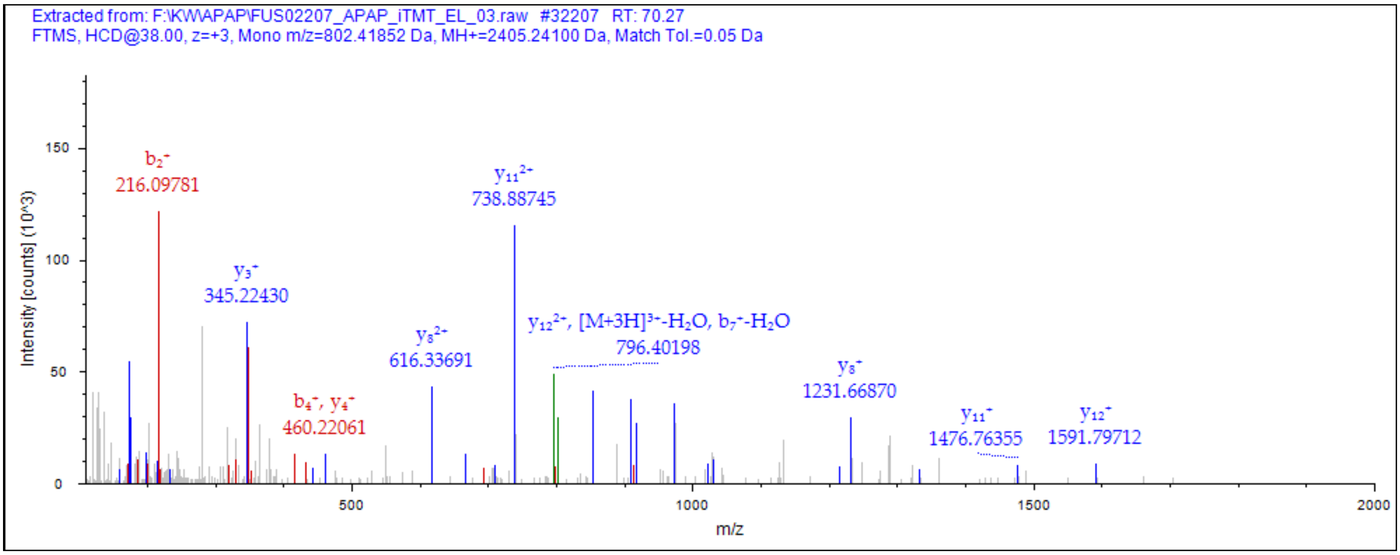

Sequence: TYFSCTSAHTSTGDGTAMITR, C5-TMT6-Cys (329.22660 Da), M18-Oxidation (15.99492 Da)
Charge: +4, Monoisotopic m/z: 639.05280 Da (-0.46 mmu/-0.72 ppm), MH+: 2553.18935 Da, RT: 26.15 min,
Identified with: Mascot (v1.30); IonScore:37, Exp Value:4.4E-003, Ions matched by search engine: 11/160
Fragment match tolerance used for search: 0.05 Da

b and y ion series

#1	a^{+}	a^{2+}	a^{3+}	a^{4+}	b^{+}	b^{2+}	b^{3+}	b^{4+}	Seq.	y^{+}	y^{2+}	y^{3+}	y^{4+}	#2
1	74.06004	37.53366	25.35820	19.27047	102.05496	51.53112	34.68984	26.26920	T					21
2	237.12336	119.06532	79.71264	60.03630	265.11828	133.06278	89.04428	67.03503	Y	2452.14350	1226.57539	818.05268	613.79133	20
3	384.19178	192.59953	128.73545	96.80340	412.18670	206.59699	138.06708	103.80213	F	2289.08018	1145.04373	763.69824	573.02550	19
4	471.22381	236.11554	157.74612	118.56141	499.21873	250.11300	167.07776	125.56014	S	2142.01176	1071.50952	714.67544	536.25840	18
5	903.45960	452.23344	301.82472	226.62036	931.45451	466.23089	311.15635	233.61909	C-TMT6	2054.97973	1027.99350	685.66476	514.50039	17
6	1004.50728	502.75728	335.50728	251.88228	1032.50219	516.75473	344.83891	258.88101	T	1622.74395	811.87561	541.58617	406.44144	16
7	1091.53931	546.27329	364.51795	273.64028	1119.53422	560.27075	373.84959	280.63901	S	1521.69627	761.35177	507.90361	381.17952	15
8	1162.57643	581.79185	388.19699	291.39956	1190.57134	595.78931	397.52863	298.39829	A	1434.66424	717.83576	478.89293	359.42152	14
9	1299.63534	650.32131	433.88330	325.66429	1327.63025	664.31876	443.21493	332.66302	H	1363.62712	682.31720	455.21389	341.66224	13
10	1400.68302	700.84515	467.56586	350.92621	1428.67793	714.84260	476.89749	357.92494	T	1226.56821	613.78774	409.52759	307.39751	12
11	1487.71505	744.36116	496.57653	372.68422	1515.70996	758.35862	505.90817	379.68295	S	1125.52053	563.26390	375.84503	282.13559	11
12	1588.76273	794.88500	530.25909	397.94614	1616.75764	808.88246	539.59073	404.94487	T	1038.48850	519.74789	346.83435	260.37758	10
13	1645.78420	823.39574	549.26625	412.20151	1673.77911	837.39319	558.59789	419.20024	G	937.44082	469.22405	313.15179	235.11566	9
14	1760.81115	880.90921	587.60857	440.95824	1788.80606	894.90667	596.94020	447.95697	D	880.41935	440.71331	294.14463	220.86029	8
15	1817.83262	909.41995	606.61572	455.21361	1845.82753	923.41740	615.94736	462.21234	G	765.39240	383.19984	255.80232	192.10356	7
16	1918.88030	959.94379	640.29828	480.47553	1946.87521	973.94124	649.62992	487.47426	T	708.37093	354.68910	236.79516	177.84819	6
17	1989.91742	995.46235	663.97732	498.23481	2017.91233	1009.45980	673.30896	505.23354	A	607.32325	304.16526	203.11260	152.58627	5
18	2136.95283	1068.98005	712.98913	534.99367	2164.94775	1082.97751	722.32077	541.99239	M-Oxidati	536.28613	268.64670	179.43356	134.82699	4
19	2250.03690	1125.52209	750.68382	563.26468	2278.03182	1139.51955	760.01546	570.26341	I	389.25071	195.12899	130.42175	98.06814	3
20	2351.08458	1176.04593	784.36638	588.52660	2379.07950	1190.04339	793.69802	595.52533	T	276.16664	138.58696	92.72706	69.79712	2
21									R	175.11896	88.06312	59.04450	44.53520	1

annotated MSMS spectrum

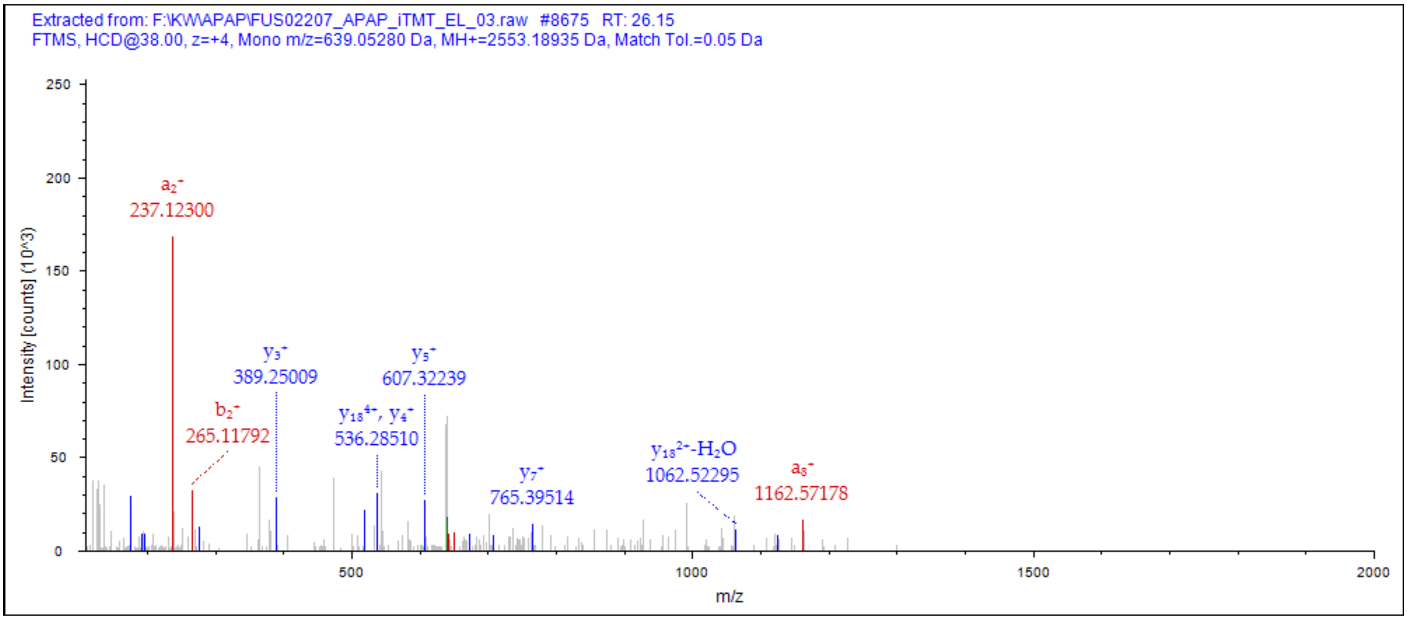

Sequence: AALEALGSCLNNK, C9-TMT6-Cys (329.22660 Da)
Charge: +2, Monoisotopic m/z: 816.95148 Da (+0.78 mmu/+0.95 ppm), MH+: 1632.89568 Da, RT: 40.30 min,
Identified with: Mascot (v1.30); IonScore:31, Exp Value:2.9E-002, Ions matched by search engine: 10/104
Fragment match tolerance used for search: 0.05 Da

b and y ion series

#1	a^+	a^{2+}	b^+	b^{2+}	Seq.	y^+	y^{2+}	#2
1	44.04948	22.52838	72.04440	36.52584	A			13
2	115.08660	58.04694	143.08152	72.04440	A	1561.85701	781.43214	12
3	228.17067	114.58897	256.16559	128.58643	L	1490.81989	745.91358	11
4	357.21327	179.11027	385.20819	193.10773	E	1377.73582	689.37155	10
5	428.25039	214.62883	456.24531	228.62629	A	1248.69322	624.85025	9
6	541.33446	271.17087	569.32938	285.16833	L	1177.65610	589.33169	8
7	598.35593	299.68160	626.35085	313.67906	G	1064.57203	532.78965	7
8	685.38796	343.19762	713.38288	357.19508	S	1007.55056	504.27892	6
9	1117.62375	559.31551	1145.61866	573.31297	C-TMT6-	920.51853	460.76290	5
10	1230.70782	615.85755	1258.70273	629.85500	L	488.28274	244.64501	4
11	1344.75075	672.87901	1372.74566	686.87647	N	375.19867	188.10297	3
12	1458.79368	729.90048	1486.78859	743.89793	N	261.15574	131.08151	2
13					K	147.11281	74.06004	1

annotated MSMS spectrum

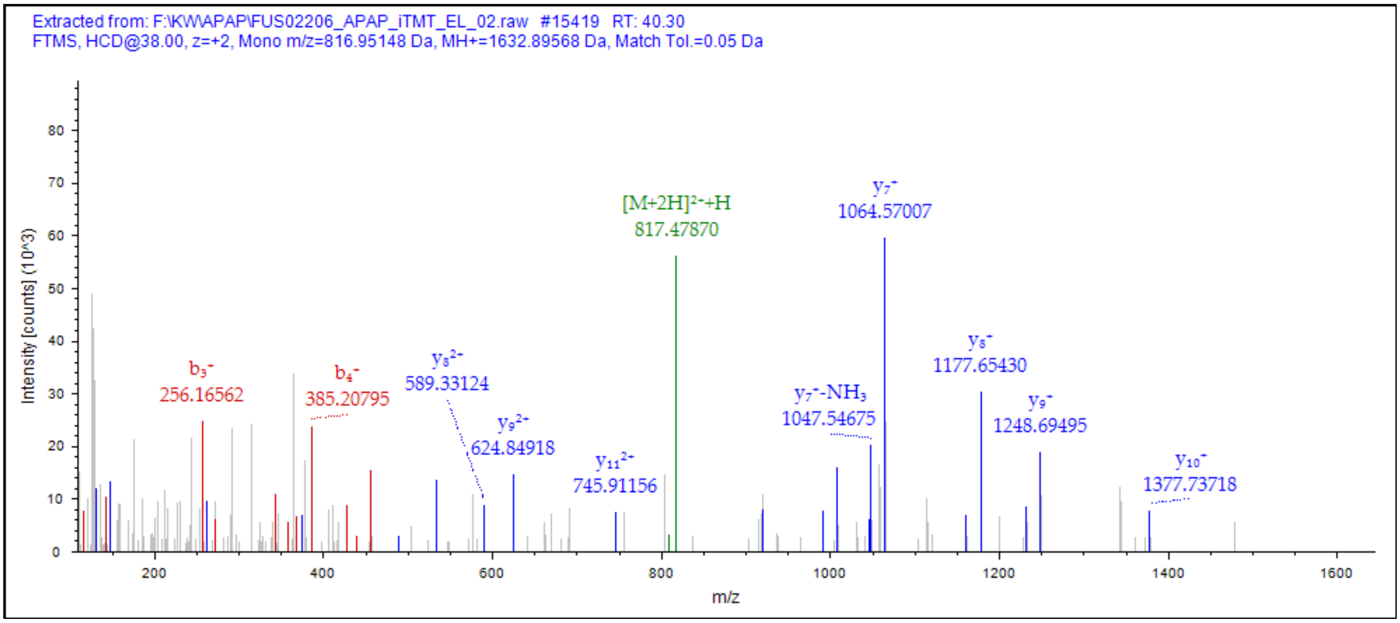

Sequence: LACGVIGIAQ, C3-TMT6-Cys (329.22660 Da)
Charge: +2, Monoisotopic m/z: 637.37830 Da (-0.35 mmu/-0.55 ppm), MH+: 1273.74932 Da, RT: 47.59 min,
Identified with: Mascot (v1.30); IonScore:37, Exp Value:2.9E-003, Ions matched by search engine: 6/72
Fragment match tolerance used for search: 0.05 Da

b and y ion series

#1	a^+	a^{2+}	b^+	b^{2+}	Seq.	y^+	y^{2+}	#2
1	86.09643	43.55185	114.09135	57.54931	L			10
2	157.13355	79.07041	185.12847	93.06787	A	1160.66595	580.83661	9
3	589.36934	295.18831	617.36425	309.18576	C-TMT6-	1089.62883	545.31805	8
4	646.39081	323.69904	674.38572	337.69650	G	657.39304	329.20016	7
5	745.45923	373.23325	773.45414	387.23071	V	600.37157	300.68942	6
6	858.54330	429.77529	886.53821	443.77274	I	501.30315	251.15521	5
7	915.56477	458.28602	943.55968	472.28348	G	388.21908	194.61318	4
8	1028.64884	514.82806	1056.64375	528.82551	I	331.19761	166.10244	3
9	1099.68596	550.34662	1127.68087	564.34407	A	218.11354	109.56041	2
10					Q	147.07642	74.04185	1

annotated MSMS spectrum

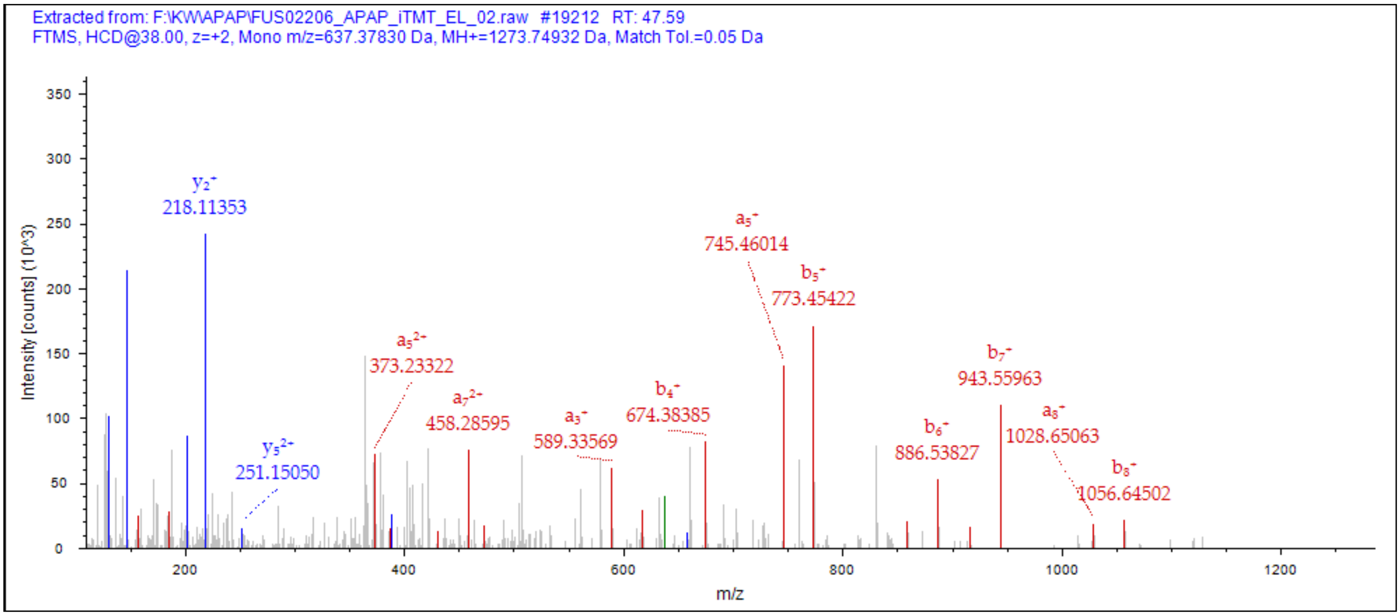

Sequence: KVDLSQPLIATCR, C12-TMT6-Cys (329.22660 Da)
Charge: +3, Monoisotopic m/z: 591.67981 Da (-0.2 mmu/-0.34 ppm), MH+: 1773.02488 Da, RT: 37.55 min,
Identified with: Mascot (v1.30); IonScore:25, Exp Value:2.9E-002, Ions matched by search engine: 16/144
Fragment match tolerance used for search: 0.05 Da

b and y ion series

#1	a^{+}	a^{2+}	a^{3+}	b^{+}	b^{2+}	b^{3+}	Seq.	y^{+}	y^{2+}	y^{3+}	#2
1	101.10733	51.05730	34.37396	129.10225	65.05476	43.70560	K				13
2	200.17575	100.59151	67.39677	228.17067	114.58897	76.72841	V	1644.93051	822.96889	548.98169	12
3	315.20270	158.10499	105.73909	343.19762	172.10245	115.07072	D	1545.86209	773.43468	515.95888	11
4	428.28677	214.64702	143.43378	456.28169	228.64448	152.76541	L	1430.83514	715.92121	477.61656	10
5	515.31880	258.16304	172.44445	543.31372	272.16050	181.77609	S	1317.75107	659.37917	439.92187	9
6	643.37738	322.19233	215.13065	671.37230	336.18979	224.46228	Q	1230.71904	615.86316	410.91120	8
7	740.43015	370.71871	247.48157	768.42507	384.71617	256.81321	P	1102.66046	551.83387	368.22500	7
8	853.51422	427.26075	285.17626	881.50914	441.25821	294.50790	L	1005.60769	503.30748	335.87408	6
9	966.59829	483.80278	322.87095	994.59321	497.80024	332.20259	I	892.52362	446.76545	298.17939	5
10	1037.63541	519.32134	346.54999	1065.63033	533.31880	355.88163	A	779.43955	390.22341	260.48470	4
11	1138.68309	569.84518	380.23255	1166.67801	583.84264	389.56419	T	708.40243	354.70485	236.80566	3
12	1570.91888	785.96308	524.31114	1598.91379	799.96053	533.64278	C-TMT6-	607.35475	304.18101	203.12310	2
13							R	175.11896	88.06312	59.04450	1

annotated MSMS spectrum

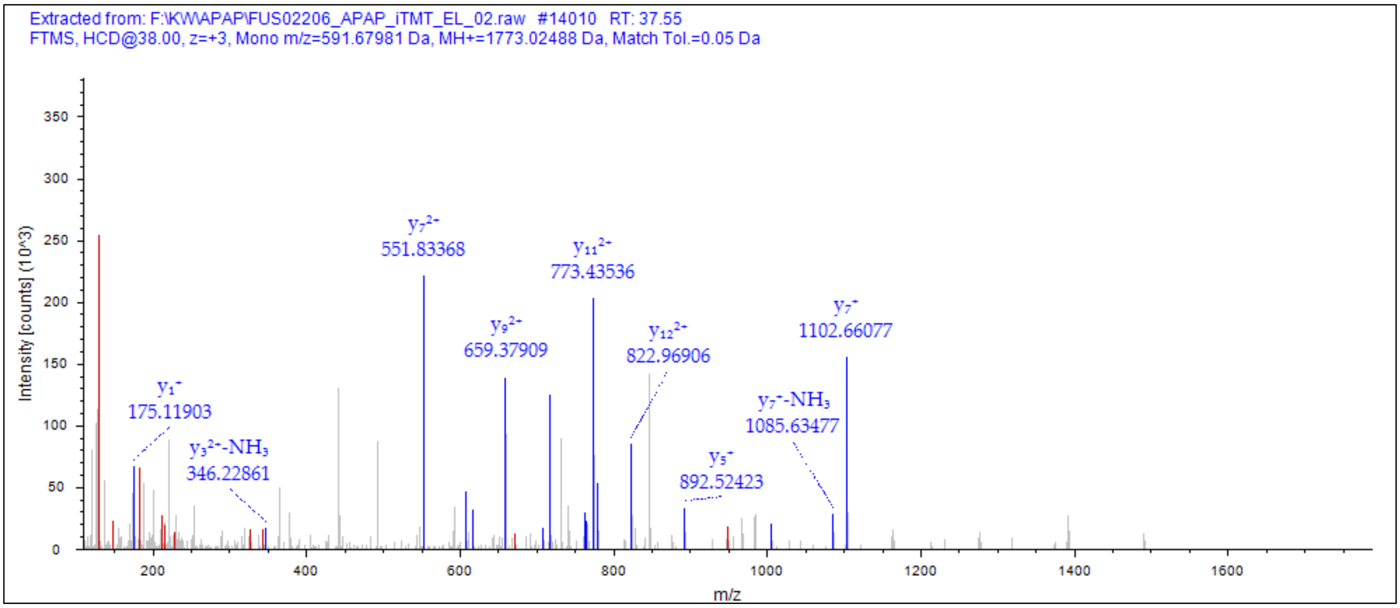

Sequence: AKPYEGSILEADCDILIPAASEK, C13-TMT6-Cys (329.22660 Da)
Charge: +3, Monoisotopic m/z: 921.48828 Da (+0.6 mmu/+0.65 ppm), MH+: 2762.45029 Da, RT: 60.92 min,
Identified with: Mascot (v1.30); IonScore:18, Exp Value:5.0E-001, Ions matched by search engine: 19/260
Fragment match tolerance used for search: 0.05 Da

b and y ion series

#1	a^+	a^{2+}	a^{3+}	b^+	b^{2+}	b^{3+}	Seq.	y^+	y^{2+}	y^{3+}	#2
1	44.04948	22.52838	15.35468	72.04440	36.52584	24.68632	A				23
2	172.14445	86.57586	58.05300	200.13937	100.57332	67.38464	K	2691.41137	1346.20932	897.80864	22
3	269.19722	135.10225	90.40393	297.19214	149.09971	99.73556	P	2563.31640	1282.16184	855.11032	21
4	432.26054	216.63391	144.75837	460.25546	230.63137	154.09000	Y	2466.26363	1233.63545	822.75939	20
5	561.30314	281.15521	187.77257	589.29806	295.15267	197.10420	E	2303.20031	1152.10379	768.40495	19
6	618.32461	309.66594	206.77972	646.31953	323.66340	216.11136	G	2174.15771	1087.58249	725.39075	18
7	705.35664	353.18196	235.79040	733.35156	367.17942	245.12204	S	2117.13624	1059.07176	706.38360	17
8	818.44071	409.72399	273.48509	846.43563	423.72145	282.81673	I	2030.10421	1015.55574	677.37292	16
9	931.52478	466.26603	311.17978	959.51970	480.26349	320.51142	L	1917.02014	959.01371	639.67823	15
10	1060.56738	530.78733	354.19398	1088.56230	544.78479	363.52562	E	1803.93607	902.47167	601.98354	14
11	1131.60450	566.30589	377.87302	1159.59942	580.30335	387.20466	A	1674.89347	837.95037	558.96934	13
12	1246.63145	623.81936	416.21534	1274.62637	637.81682	425.54697	D	1603.85635	802.43181	535.29030	12
13	1678.86724	839.93726	560.29393	1706.86215	853.93471	569.62557	C-TMT6-	1488.82940	744.91834	496.94798	11
14	1793.89419	897.45073	598.63625	1821.88910	911.44819	607.96788	D	1056.59361	528.80044	352.86939	10
15	1906.97826	953.99277	636.33094	1934.97317	967.99022	645.66257	I	941.56666	471.28697	314.52707	9
16	2020.06233	1010.53480	674.02563	2048.05724	1024.53226	683.35726	L	828.48259	414.74493	276.83238	8
17	2133.14640	1067.07684	711.72032	2161.14131	1081.07429	721.05195	I	715.39852	358.20290	239.13769	7
18	2230.19917	1115.60322	744.07124	2258.19408	1129.60068	753.40288	P	602.31445	301.66086	201.44300	6
19	2301.23629	1151.12178	767.75028	2329.23120	1165.11924	777.08192	A	505.26168	253.13448	169.09208	5
20	2372.27341	1186.64034	791.42932	2400.26832	1200.63780	800.76096	A	434.22456	217.61592	145.41304	4
21	2459.30544	1230.15636	820.44000	2487.30035	1244.15381	829.77163	S	363.18744	182.09736	121.73400	3
22	2588.34804	1294.67766	863.45420	2616.34295	1308.67511	872.78583	E	276.15541	138.58134	92.72332	2
23							K	147.11281	74.06004	49.70912	1

annotated MSMS spectrum

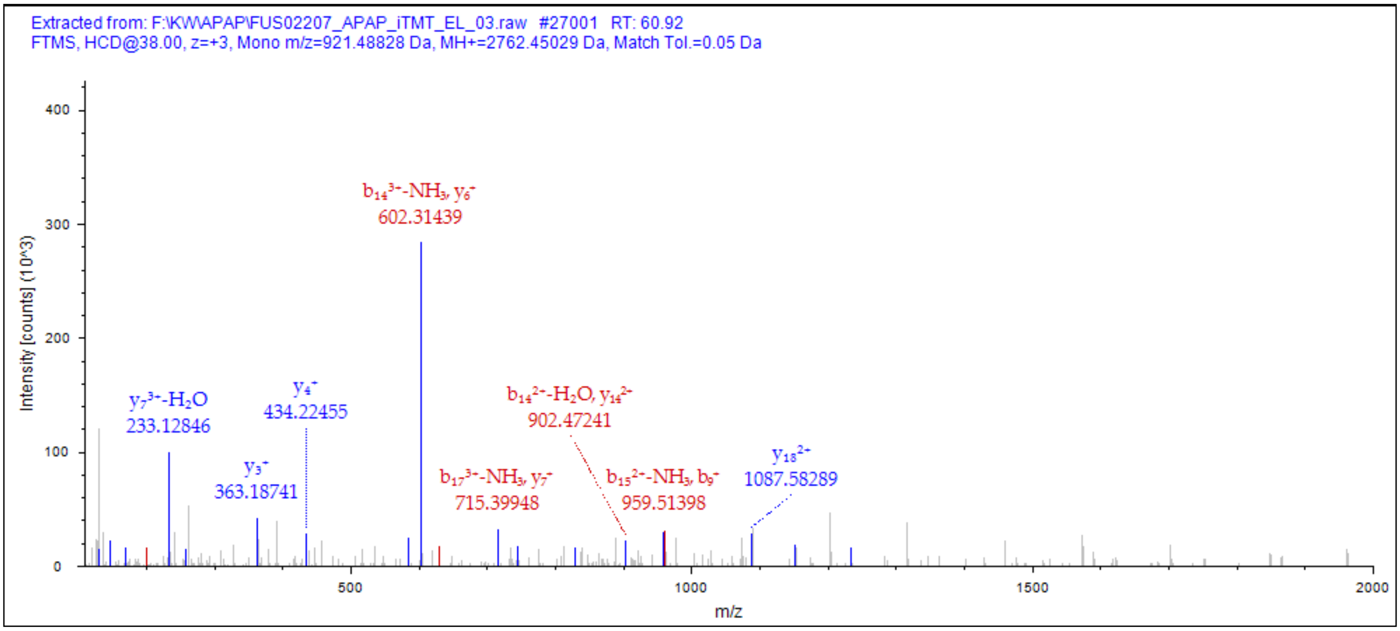

Sequence: CELSSSVQTDINLPYLTMDSSGPK, C1-TMT6-Cys (329.22660 Da), M18-Oxidation (15.99492 Da)
Charge: +3, Monoisotopic m/z: 977.48328 Da (+0.91 mmu/+0.93 ppm), MH+: 2930.43528 Da, RT: 67.20 min,
Identified with: Mascot (v1.30); IonScore:56, Exp Value:1.0E-004, Ions matched by search engine: 19/248
Fragment match tolerance used for search: 0.05 Da

b and y ion series

#1	a^+	a^{2+}	a^{3+}	b^+	b^{2+}	b^{3+}	Seq.	y^+	y^{2+}	y^{3+}	#2
1	405.24815	203.12771	135.75423	433.24306	217.12517	145.08587	C-TMT6-				24
2	534.29075	267.64901	178.76843	562.28566	281.64647	188.10007	E	2498.19678	1249.60203	833.40378	23
3	647.37482	324.19105	216.46312	675.36973	338.18850	225.79476	L	2369.15418	1185.08073	790.38958	22
4	734.40685	367.70706	245.47380	762.40176	381.70452	254.80544	S	2256.07011	1128.53869	752.69489	21
5	821.43888	411.22308	274.48448	849.43379	425.22053	283.81611	S	2169.03808	1085.02268	723.68421	20
6	908.47091	454.73909	303.49515	936.46582	468.73655	312.82679	S	2082.00605	1041.50666	694.67353	19
7	1007.53933	504.27330	336.51796	1035.53424	518.27076	345.84960	V	1994.97402	997.99065	665.66286	18
8	1135.59791	568.30259	379.20415	1163.59282	582.30005	388.53579	Q	1895.90560	948.45644	632.64005	17
9	1236.64559	618.82643	412.88671	1264.64050	632.82389	422.21835	T	1767.84702	884.42715	589.95386	16
10	1351.67254	676.33991	451.22903	1379.66745	690.33736	460.56067	D	1666.79934	833.90331	556.27130	15
11	1464.75661	732.88194	488.92372	1492.75152	746.87940	498.25536	I	1551.77239	776.38983	517.92898	14
12	1578.79954	789.90341	526.93803	1606.79445	803.90086	536.26967	N	1438.68832	719.84780	480.23429	13
13	1691.88361	846.44544	564.63272	1719.87852	860.44290	573.96436	L	1324.64539	662.82633	442.21998	12
14	1788.93638	894.97183	596.98364	1816.93129	908.96928	606.31528	P	1211.56132	606.28430	404.52529	11
15	1951.99970	976.50349	651.33808	1979.99461	990.50094	660.66972	Y	1114.50855	557.75791	372.17437	10
16	2065.08377	1033.04552	689.03277	2093.07868	1047.04298	698.36441	L	951.44523	476.22625	317.81993	9
17	2166.13145	1083.56936	722.71533	2194.12636	1097.56682	732.04697	T	838.36116	419.68422	280.12524	8
18	2313.16686	1157.08707	771.72714	2341.16178	1171.08453	781.05878	M-Oxidation	737.31348	369.16038	246.44268	7
19	2428.19381	1214.60054	810.06946	2456.18873	1228.59800	819.40109	D	590.27806	295.64267	197.43087	6
20	2515.22584	1258.11656	839.08013	2543.22076	1272.11402	848.41177	S	475.25111	238.12919	159.08855	5
21	2602.25787	1301.63257	868.09081	2630.25279	1315.63003	877.42245	S	388.21908	194.61318	130.07788	4
22	2659.27934	1330.14331	887.09797	2687.27426	1344.14077	896.42960	G	301.18705	151.09716	101.06720	3
23	2756.33211	1378.66969	919.44889	2784.32703	1392.66715	928.78053	P	244.16558	122.58643	82.06004	2
24							K	147.11281	74.06004	49.70912	1

annotated MSMS spectrum

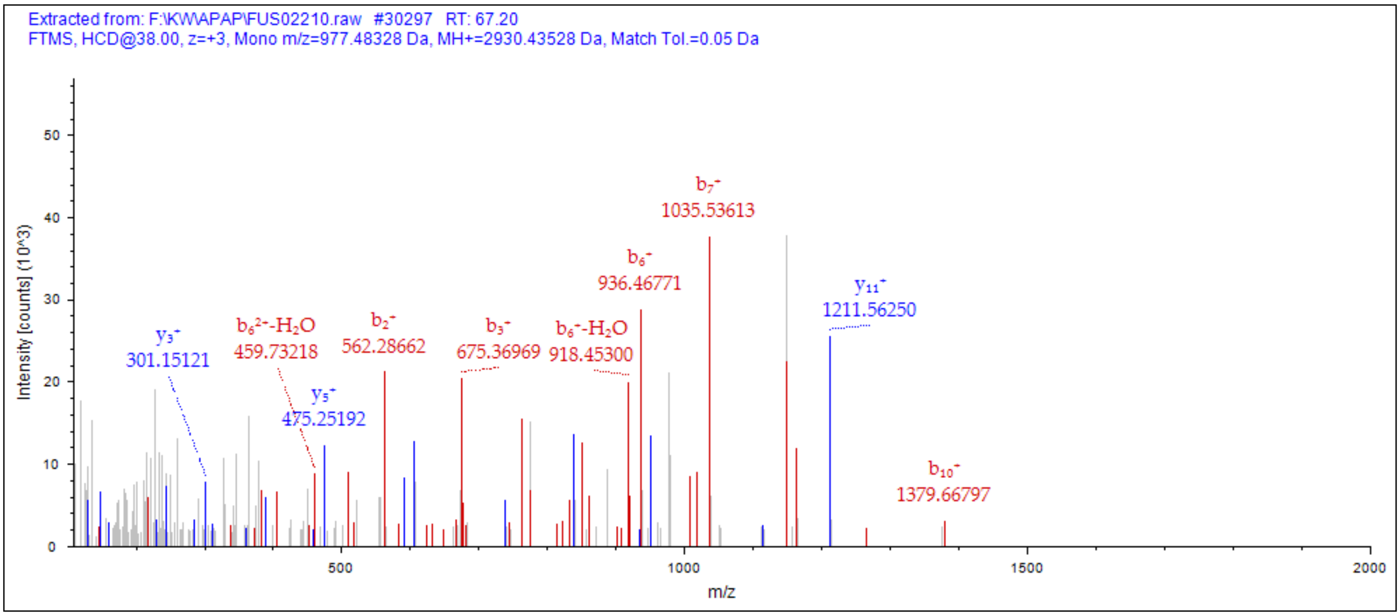

Sequence: NALVSHLDGTTPVCEDIGR, C14-TMT6-Cys (329.22660 Da)
Charge: +3, Monoisotopic m/z: 776.07196 Da (-0.34 mmu/-0.44 ppm), MH+: 2326.20133 Da, RT: 43.07 min,
Identified with: Mascot (v1.30); IonScore:40, Exp Value:3.9E-003, Ions matched by search engine: 11/216
Fragment match tolerance used for search: 0.05 Da

b and y ion series

#1	a^+	a^{2+}	a^{3+}	b^+	b^{2+}	b^{3+}	Seq.	y^+	y^{2+}	y^{3+}	#2
1	87.05529	44.03128	29.68995	115.05021	58.02874	39.02159	N				19
2	158.09241	79.54984	53.36899	186.08733	93.54730	62.70063	A	2212.15943	1106.58335	738.05799	18
3	271.17648	136.09188	91.06368	299.17140	150.08934	100.39532	L	2141.12231	1071.06479	714.37895	17
4	370.24490	185.62609	124.08649	398.23982	199.62355	133.41812	V	2028.03824	1014.52276	676.68426	16
5	457.27693	229.14210	153.09716	485.27185	243.13956	162.42880	S	1928.96982	964.98855	643.66146	15
6	594.33584	297.67156	198.78347	622.33076	311.66902	208.11510	H	1841.93779	921.47253	614.65078	14
7	707.41991	354.21359	236.47816	735.41483	368.21105	245.80979	L	1704.87888	852.94308	568.96448	13
8	822.44686	411.72707	274.82047	850.44178	425.72453	284.15211	D	1591.79481	796.40104	531.26979	12
9	879.46833	440.23780	293.82763	907.46325	454.23526	303.15927	G	1476.76786	738.88757	492.92747	11
10	980.51601	490.76164	327.51019	1008.51093	504.75910	336.84183	T	1419.74639	710.37683	473.92031	10
11	1081.56369	541.28548	361.19275	1109.55861	555.28294	370.52439	T	1318.69871	659.85299	440.23775	9
12	1178.61646	589.81187	393.54367	1206.61138	603.80933	402.87531	P	1217.65103	609.32915	406.55519	8
13	1277.68488	639.34608	426.56648	1305.67980	653.34354	435.89812	V	1120.59826	560.80277	374.20427	7
14	1709.92067	855.46397	570.64507	1737.91558	869.46143	579.97671	C-TMT6-	1021.52984	511.26856	341.18146	6
15	1838.96327	919.98527	613.65927	1866.95818	933.98273	622.99091	E	589.29405	295.15066	197.10287	5
16	1953.99022	977.49875	652.00159	1981.98513	991.49620	661.33323	D	460.25145	230.62936	154.08867	4
17	2067.07429	1034.04078	689.69628	2095.06920	1048.03824	699.02792	I	345.22450	173.11589	115.74635	3
18	2124.09576	1062.55152	708.70344	2152.09067	1076.54897	718.03507	G	232.14043	116.57385	78.05166	2
19							R	175.11896	88.06312	59.04450	1

annotated MSMS spectrum

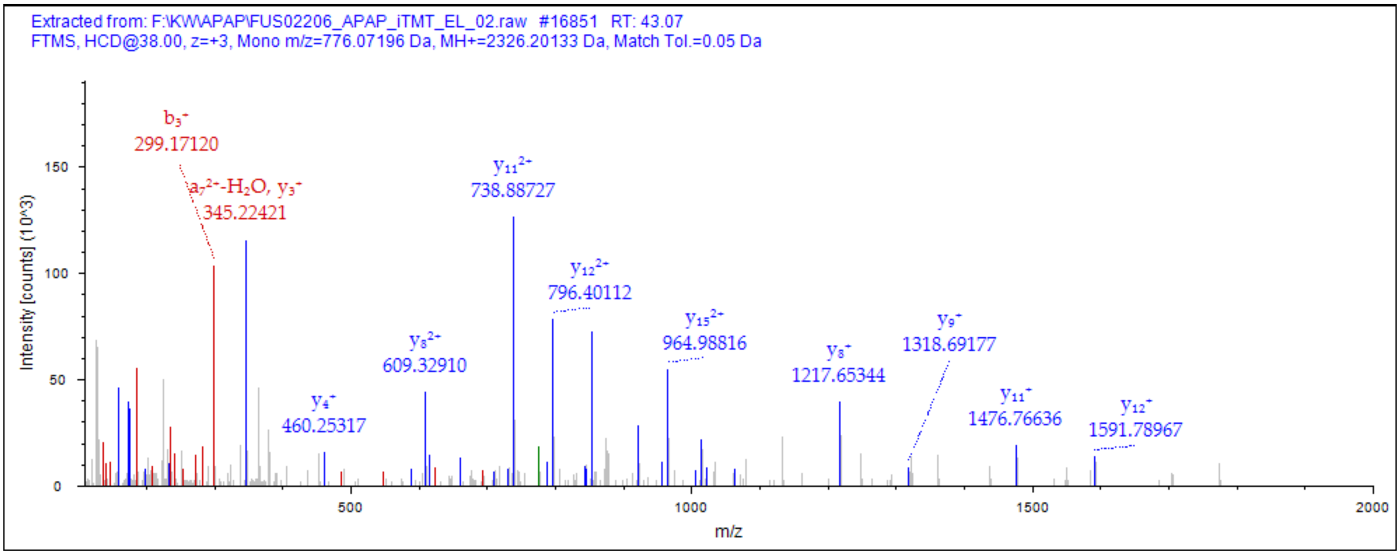

Sequence: VCLLGCGISTGYGAAVNTAK, C2-TMT6-Cys (329.22660 Da), C6-TMT6-Cys (329.22660 Da)
Charge: +4, Monoisotopic m/z: 639.85773 Da (+1.19 mmu/+1.86 ppm), MH+: 2556.40908 Da, RT: 50.84 min,
Identified with: Mascot (v1.30); IonScore:32, Exp Value:7.7E-003, Ions matched by search engine: 8/164
Fragment match tolerance used for search: 0.05 Da

b and y ion series

#1	a^+	a^{2+}	a^{3+}	a^{4+}	b^+	b^{2+}	b^{3+}	b^{4+}	Seq.	y^+	y^{2+}	y^{3+}	y^{4+}	#2
1	72.08078	36.54403	24.69845	18.77565	100.07570	50.54149	34.03008	25.77438	V					20
2	504.31657	252.66192	168.77704	126.83460	532.31148	266.65938	178.10868	133.83333	C-TMT6-	2457.33589	1229.17158	819.78348	615.08943	19
3	617.40064	309.20396	206.47173	155.10562	645.39555	323.20141	215.80337	162.10435	L	2025.10011	1013.05369	675.70489	507.03048	18
4	730.48471	365.74599	244.16642	183.37663	758.47962	379.74345	253.49806	190.37536	L	1912.01604	956.51166	638.01020	478.75947	17
5	787.50618	394.25673	263.17358	197.63200	815.50109	408.25418	272.50521	204.63073	G	1798.93197	899.96962	600.31551	450.48845	16
6	1219.74196	610.37462	407.25217	305.69095	1247.73688	624.37208	416.58381	312.68968	C-TMT6-	1741.91050	871.45889	581.30835	436.23308	15
7	1276.76343	638.88535	426.25933	319.94632	1304.75835	652.88281	435.59097	326.94504	G	1309.67471	655.34099	437.22975	328.17414	14
8	1389.84750	695.42739	463.95402	348.21733	1417.84242	709.42485	473.28566	355.21606	I	1252.65324	626.83026	418.22260	313.91877	13
9	1476.87953	738.94340	492.96470	369.97534	1504.87445	752.94086	502.29633	376.97407	S	1139.56917	570.28822	380.52791	285.64775	12
10	1577.92721	789.46724	526.64726	395.23726	1605.92213	803.46470	535.97889	402.23599	T	1052.53714	526.77221	351.51723	263.88974	11
11	1634.94868	817.97798	545.65441	409.49263	1662.94360	831.97544	554.98605	416.49136	G	951.48946	476.24837	317.83467	238.62782	10
12	1798.01200	899.50964	600.00885	450.25846	1826.00692	913.50710	609.34049	457.25719	Y	894.46799	447.73763	298.82751	224.37246	9
13	1855.03347	928.02037	619.01601	464.51383	1883.02839	942.01783	628.34765	471.51255	G	731.40467	366.20597	244.47307	183.60663	8
14	1926.07059	963.53893	642.69505	482.27311	1954.06551	977.53639	652.02669	489.27183	A	674.38320	337.69524	225.46592	169.35126	7
15	1997.10771	999.05749	666.37409	500.03239	2025.10263	1013.05495	675.70573	507.03111	A	603.34608	302.17668	201.78688	151.59198	6
16	2096.17613	1048.59170	699.39690	524.79949	2124.17105	1062.58916	708.72853	531.79822	V	532.30896	266.65812	178.10784	133.83270	5
17	2210.21906	1105.61317	737.41121	553.31022	2238.21398	1119.61063	746.74284	560.30895	N	433.24054	217.12391	145.08503	109.06559	4
18	2311.26674	1156.13701	771.09377	578.57214	2339.26166	1170.13447	780.42540	585.57087	T	319.19761	160.10244	107.07072	80.55486	3
19	2382.30386	1191.65557	794.77281	596.33142	2410.29878	1205.65303	804.10444	603.33015	A	218.14993	109.57860	73.38816	55.29294	2
20									K	147.11281	74.06004	49.70912	37.53366	1

annotated MSMS spectrum

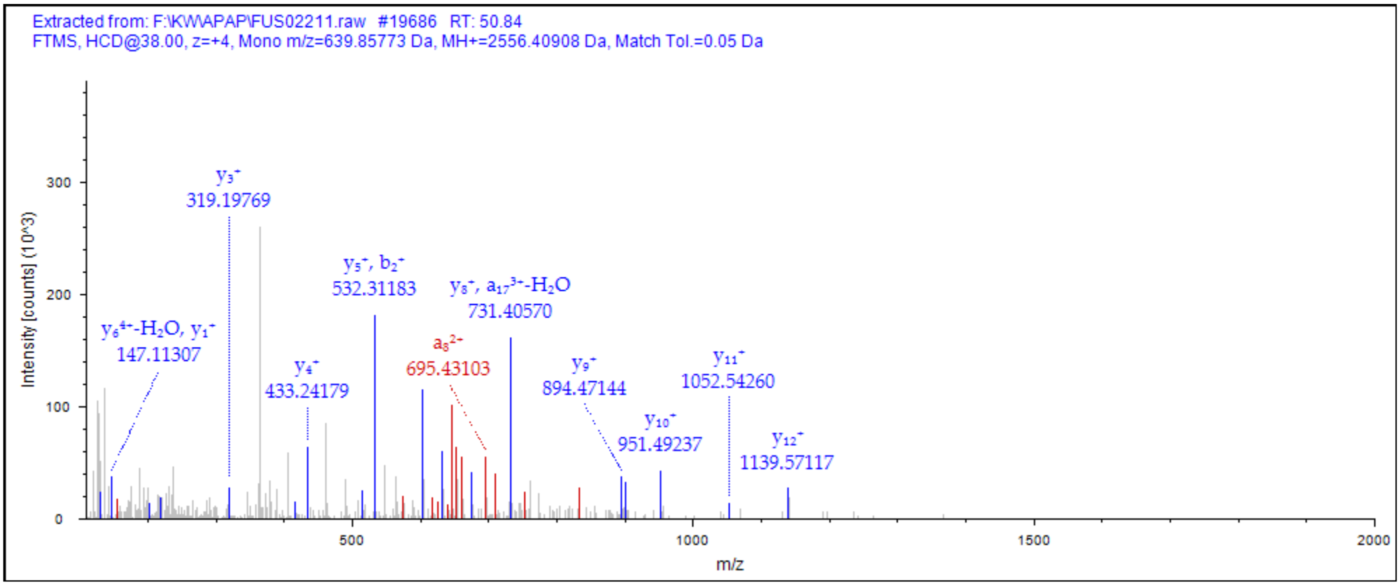

Sequence: SCGSLLPELK, C2-TMT6-Cys (329.22660 Da)
Charge: +2, Monoisotopic m/z: 688.39465 Da (+0.16 mmu/+0.24 ppm), MH+: 1375.78203 Da, RT: 42.63 min,
Identified with: Mascot (v1.30); IonScore:30, Exp Value:2.1E-002, Ions matched by search engine: 6/72
Fragment match tolerance used for search: 0.05 Da

b and y ion series

#1	a^+	a^{2+}	b^+	b^{2+}	Seq.	y^+	y^{2+}	#2
1	60.04439	30.52583	88.03931	44.52329	S			10
2	492.28018	246.64373	520.27509	260.64118	C-TMT6-	1288.74968	644.87848	9
3	549.30165	275.15446	577.29656	289.15192	G	856.51389	428.76058	8
4	636.33368	318.67048	664.32859	332.66793	S	799.49242	400.24985	7
5	749.41775	375.21251	777.41266	389.20997	L	712.46039	356.73383	6
6	862.50182	431.75455	890.49673	445.75200	L	599.37632	300.19180	5
7	959.55459	480.28093	987.54950	494.27839	P	486.29225	243.64976	4
8	1088.59719	544.80223	1116.59210	558.79969	E	389.23948	195.12338	3
9	1201.68126	601.34427	1229.67617	615.34172	L	260.19688	130.60208	2
10					K	147.11281	74.06004	1

annotated MSMS spectrum

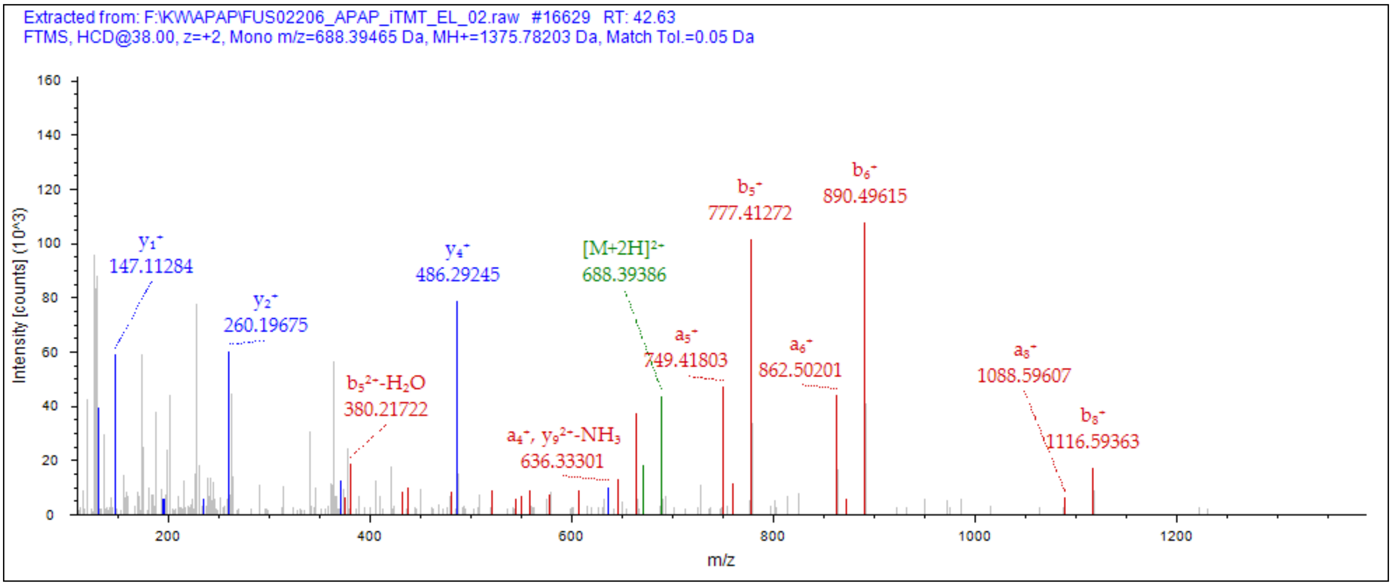

Sequence: LLDLVQQSCNYK, C9-TMT6-Cys (329.22660 Da)
Charge: +3, Monoisotopic m/z: 584.98969 Da (+0.96 mmu/+1.65 ppm), MH+: 1752.95450 Da, RT: 46.06 min,
Identified with: Mascot (v1.30); IonScore:28, Exp Value:3.1E-002, Ions matched by search engine: 6/112
Fragment match tolerance used for search: 0.05 Da

b and y ion series

#1	a⁺	a²⁺	a³⁺	b⁺	b²⁺	b³⁺	Seq.	y⁺	y²⁺	y³⁺	#2
1	86.09643	43.55185	29.37033	114.09135	57.54931	38.70197	L				12
2	199.18050	100.09389	67.06502	227.17542	114.09135	76.39666	L	1639.86755	820.43741	547.29403	11
3	314.20745	157.60736	105.40734	342.20237	171.60482	114.73897	D	1526.78348	763.89538	509.59934	10
4	427.29152	214.14940	143.10203	455.28644	228.14686	152.43366	L	1411.75653	706.38190	471.25703	9
5	526.35994	263.68361	176.12483	554.35486	277.68107	185.45647	V	1298.67246	649.83987	433.56234	8
6	654.41852	327.71290	218.81103	682.41344	341.71036	228.14266	Q	1199.60404	600.30566	400.53953	7
7	782.47710	391.74219	261.49722	810.47202	405.73965	270.82886	Q	1071.54546	536.27637	357.85334	6
8	869.50913	435.25820	290.50790	897.50405	449.25566	299.83953	S	943.48688	472.24708	315.16714	5
9	1301.74492	651.37610	434.58649	1329.73983	665.37355	443.91813	C-TMT6-	856.45485	428.73106	286.15647	4
10	1415.78785	708.39756	472.60080	1443.78276	722.39502	481.93244	N	424.21906	212.61317	142.07787	3
11	1578.85117	789.92922	526.95524	1606.84608	803.92668	536.28688	Y	310.17613	155.59170	104.06356	2
12							K	147.11281	74.06004	49.70912	1

annotated MSMS spectrum

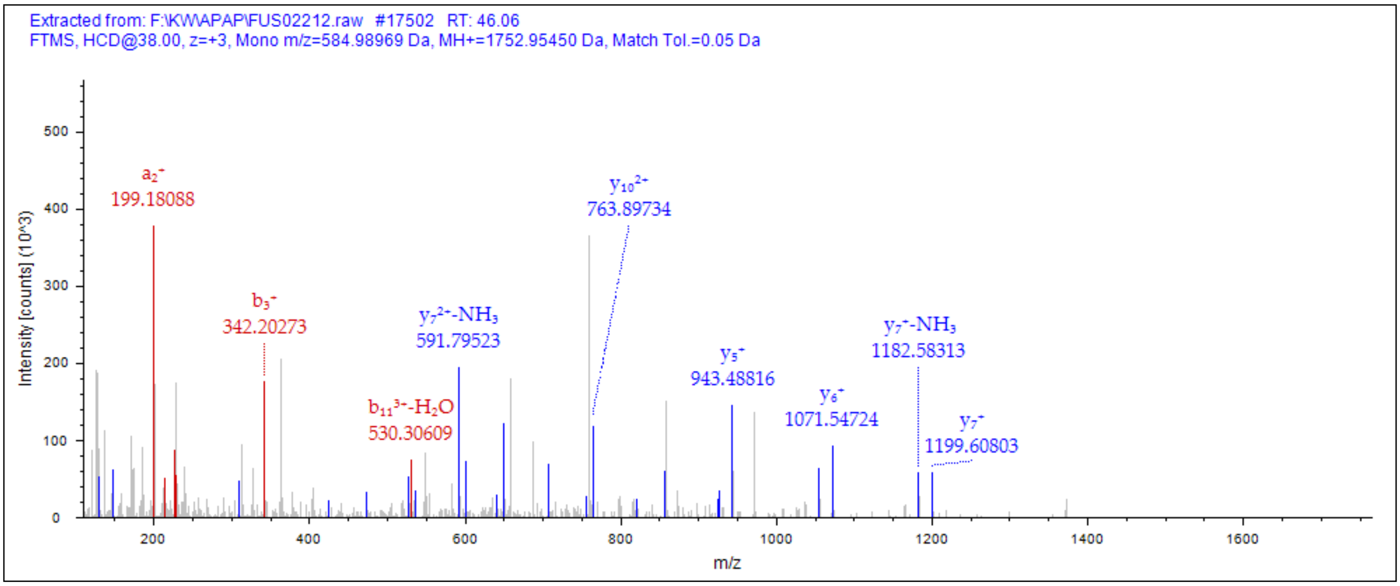

Sequence: CELLYEGPPDDEAAMGIK, C1-TMT6-Cys (329.22660 Da)
Charge: +3, Monoisotopic m/z: 760.70856 Da (+0.69 mmu/+0.91 ppm), MH+: 2280.11112 Da, RT: 59.81 min,
Identified with: Mascot (v1.30); IonScore:43, Exp Value:1.9E-003, Ions matched by search engine: 17/136
Fragment match tolerance used for search: 0.05 Da

b and y ion series

#1	a^+	a^{2+}	a^{3+}	b^+	b^{2+}	b^{3+}	Seq.	y^+	y^{2+}	y^{3+}	#2
1	405.24815	203.12771	135.75423	433.24306	217.12517	145.08587	C-TMT6-				18
2	534.29075	267.64901	178.76843	562.28566	281.64647	188.10007	E	1847.87326	924.44027	616.62927	17
3	647.37482	324.19105	216.46312	675.36973	338.18850	225.79476	L	1718.83066	859.91897	573.61507	16
4	760.45889	380.73308	254.15781	788.45380	394.73054	263.48945	L	1605.74659	803.37693	535.92038	15
5	923.52221	462.26474	308.51225	951.51712	476.26220	317.84389	Y	1492.66252	746.83490	498.22569	14
6	1052.56481	526.78604	351.52645	1080.55972	540.78350	360.85809	E	1329.59920	665.30324	443.87125	13
7	1109.58628	555.29678	370.53361	1137.58119	569.29423	379.86525	G	1200.55660	600.78194	400.85705	12
8	1206.63905	603.82316	402.88453	1234.63396	617.82062	412.21617	P	1143.53513	572.27120	381.84989	11
9	1303.69182	652.34955	435.23546	1331.68673	666.34700	444.56709	P	1046.48236	523.74482	349.49897	10
10	1418.71877	709.86302	473.57777	1446.71368	723.86048	482.90941	D	949.42959	475.21843	317.14805	9
11	1533.74572	767.37650	511.92009	1561.74063	781.37395	521.25173	D	834.40264	417.70496	278.80573	8
12	1662.78832	831.89780	554.93429	1690.78323	845.89525	564.26593	E	719.37569	360.19148	240.46341	7
13	1733.82544	867.41636	578.61333	1761.82035	881.41381	587.94497	A	590.33309	295.67018	197.44921	6
14	1804.86256	902.93492	602.29237	1832.85747	916.93237	611.62401	A	519.29597	260.15162	173.77017	5
15	1935.90306	968.45517	645.97254	1963.89797	982.45262	655.30417	M	448.25885	224.63306	150.09113	4
16	1992.92453	996.96590	664.97969	2020.91944	1010.96336	674.31133	G	317.21835	159.11281	106.41097	3
17	2106.00860	1053.50794	702.67438	2134.00351	1067.50539	712.00602	I	260.19688	130.60208	87.40381	2
18							K	147.11281	74.06004	49.70912	1

annotated MSMS spectrum

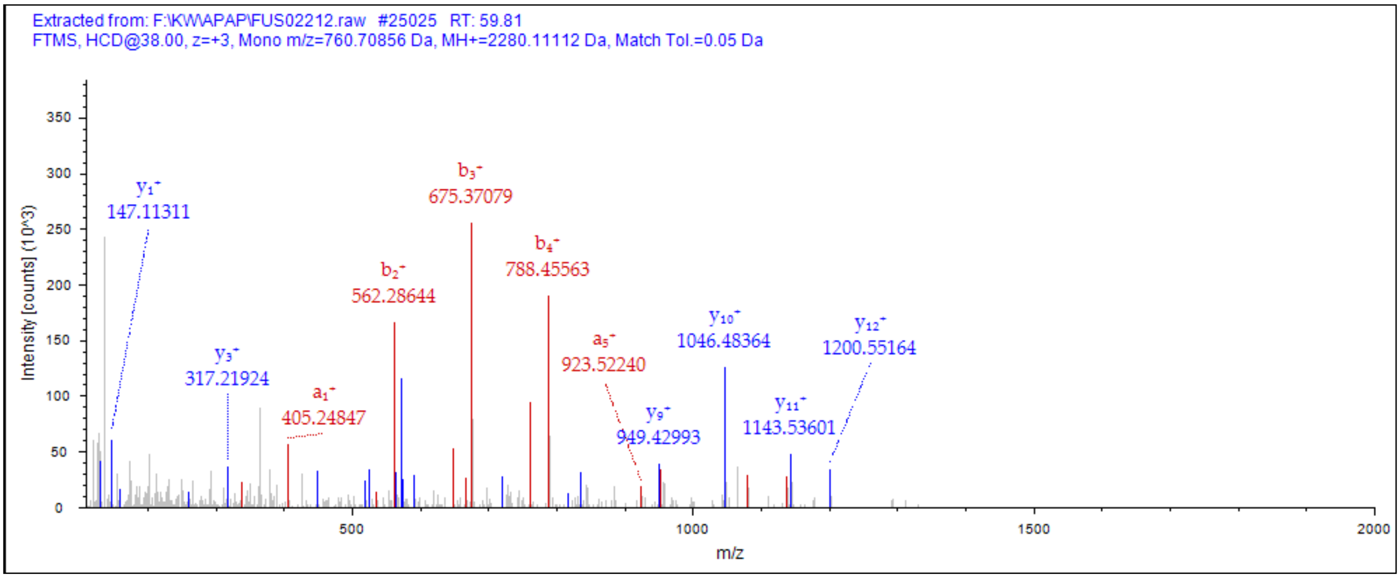

Sequence: FCIWTESAFR, C2-TMT6-Cys (329.22660 Da)
Charge: +3, Monoisotopic m/z: 530.27576 Da (-0.56 mmu/-1.06 ppm), MH+: 1588.81272 Da, RT: 59.94 min,
Identified with: Mascot (v1.30); IonScore:26, Exp Value:8.5E-002, Ions matched by search engine: 6/72
Fragment match tolerance used for search: 0.05 Da

b and y ion series

#1	a⁺	a²⁺	a³⁺	b⁺	b²⁺	b³⁺	Seq.	y⁺	y²⁺	y³⁺	#2
1	120.08078	60.54403	40.69845	148.07570	74.54149	50.03008	F				10
2	552.31657	276.66192	184.77704	580.31148	290.65938	194.10868	C-TMT6-	1441.74599	721.37663	481.25351	9
3	665.40064	333.20396	222.47173	693.39555	347.20141	231.80337	I	1009.51020	505.25874	337.17492	8
4	851.47996	426.24362	284.49817	879.47487	440.24107	293.82981	W	896.42613	448.71670	299.48023	7
5	952.52764	476.76746	318.18073	980.52255	490.76491	327.51237	T	710.34681	355.67704	237.45379	6
6	1081.57024	541.28876	361.19493	1109.56515	555.28621	370.52657	E	609.29913	305.15320	203.77123	5
7	1168.60227	584.80477	390.20561	1196.59718	598.80223	399.53724	S	480.25653	240.63190	160.75703	4
8	1239.63939	620.32333	413.88465	1267.63430	634.32079	423.21628	A	393.22450	197.11589	131.74635	3
9	1386.70781	693.85754	462.90745	1414.70272	707.85500	472.23909	F	322.18738	161.59733	108.06731	2
10							R	175.11896	88.06312	59.04450	1

annotated MSMS spectrum

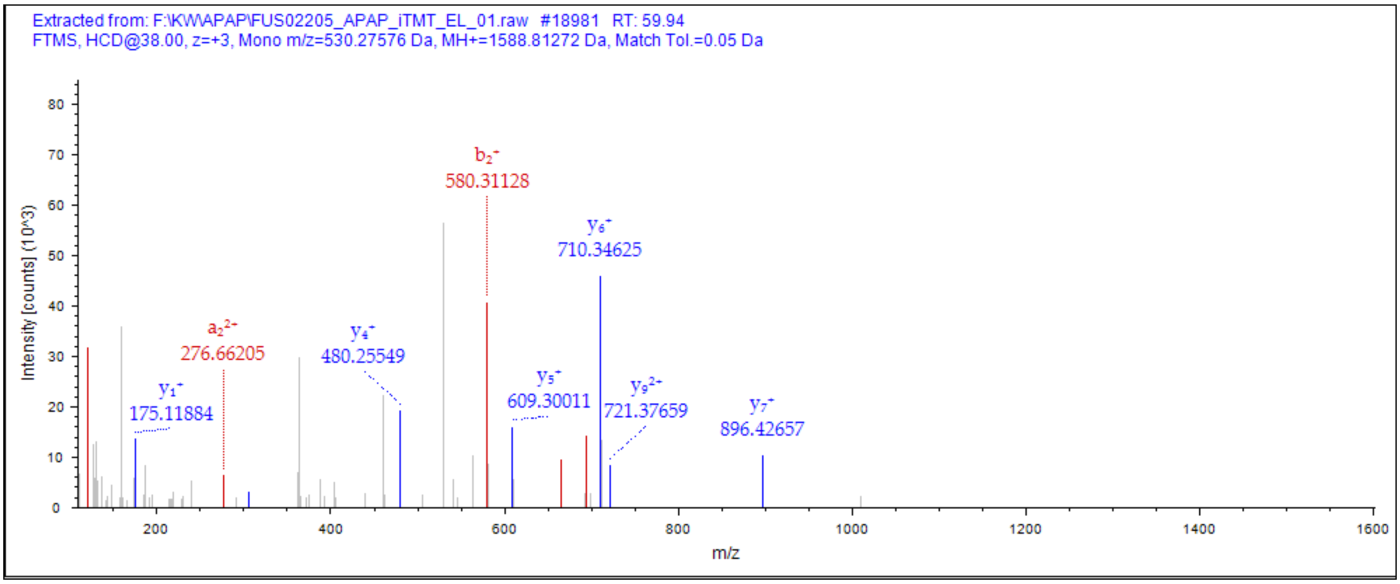

Sequence: YWLEEAECR, C8-TMT6-Cys (329.22660 Da)
Charge: +2, Monoisotopic m/z: 764.37964 Da (+2.81 mmu/+3.68 ppm), MH+: 1527.75200 Da, RT: 43.47 min,
Identified with: Mascot (v1.30); IonScore:22, Exp Value:1.6E-001, Ions matched by search engine: 5/64
Fragment match tolerance used for search: 0.05 Da

b and y ion series

#1	a^+	a^{2+}	b^+	b^{2+}	Seq.	y^+	y^{2+}	#2
1	136.07568	68.54148	164.07060	82.53894	Y			9
2	322.15500	161.58114	350.14992	175.57860	W	1364.68306	682.84517	8
3	435.23907	218.12317	463.23399	232.12063	L	1178.60374	589.80551	7
4	564.28167	282.64447	592.27659	296.64193	E	1065.51967	533.26347	6
5	693.32427	347.16577	721.31919	361.16323	E	936.47707	468.74217	5
6	764.36139	382.68433	792.35631	396.68179	A	807.43447	404.22087	4
7	893.40399	447.20563	921.39891	461.20309	E	736.39735	368.70231	3
8	1325.63978	663.32353	1353.63469	677.32098	C-TMT6-	607.35475	304.18101	2
9					R	175.11896	88.06312	1

annotated MSMS spectrum

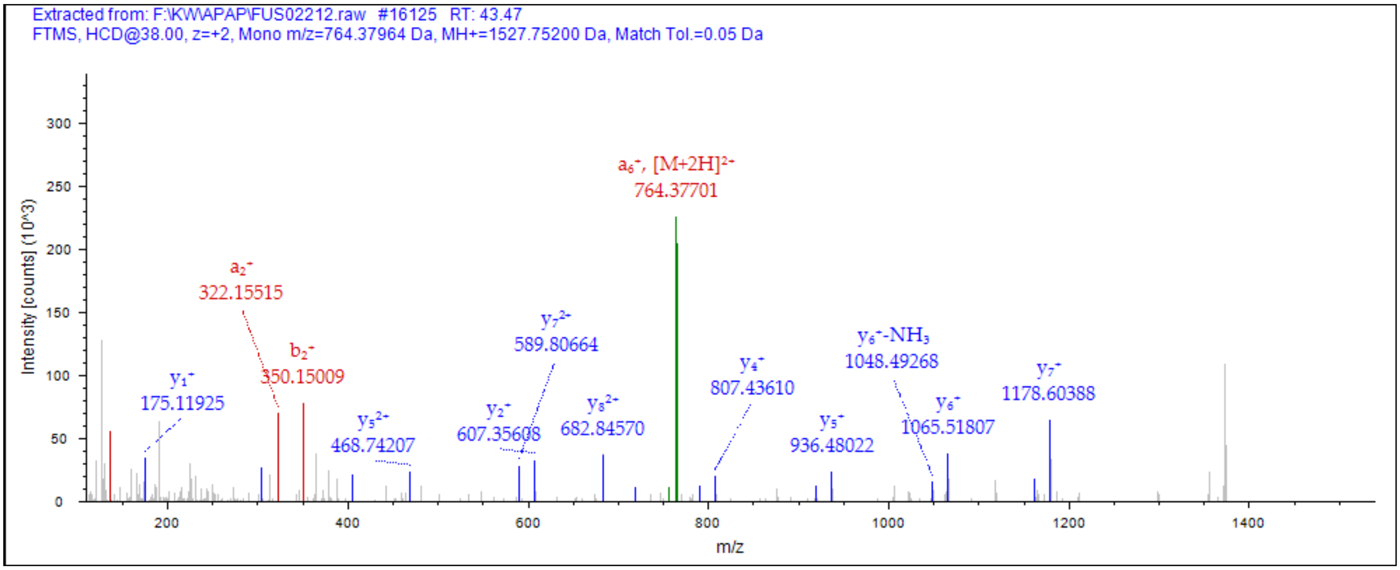

Sequence: NMSVHLSPCFR, M2-Oxidation (15.99492 Da), C9-TMT6-Cys (329.22660 Da)
Charge: +3, Monoisotopic m/z: 545.94806 Da (-0.04 mmu/-0.07 ppm), MH+: 1635.82962 Da, RT: 25.64 min,
Identified with: Mascot (v1.30); IonScore:27, Exp Value:7.8E-002, Ions matched by search engine: 6/120
Fragment match tolerance used for search: 0.05 Da

b and y ion series

#1	a^+	a^{2+}	a^{3+}	b^+	b^{2+}	b^{3+}	Seq.	y^+	y^{2+}	y^{3+}	#2
1	87.05529	44.03128	29.68995	115.05021	58.02874	39.02159	N				11
2	234.09071	117.54899	78.70175	262.08562	131.54645	88.03339	M-Oxidation	1521.78681	761.39704	507.93379	10
3	321.12274	161.06501	107.71243	349.11765	175.06246	117.04407	S	1374.75140	687.87934	458.92198	9
4	420.19116	210.59922	140.73524	448.18607	224.59667	150.06687	V	1287.71937	644.36332	429.91131	8
5	557.25007	279.12867	186.42154	585.24498	293.12613	195.75318	H	1188.65095	594.82911	396.88850	7
6	670.33414	335.67071	224.11623	698.32905	349.66816	233.44787	L	1051.59204	526.29966	351.20220	6
7	757.36617	379.18672	253.12691	785.36108	393.18418	262.45854	S	938.50797	469.75762	313.50751	5
8	854.41894	427.71311	285.47783	882.41385	441.71056	294.80947	P	851.47594	426.24161	284.49683	4
9	1286.65472	643.83100	429.55643	1314.64964	657.82846	438.88806	C-TMT6-	754.42317	377.71522	252.14591	3
10	1433.72314	717.36521	478.57923	1461.71806	731.36267	487.91087	F	322.18738	161.59733	108.06731	2
11							R	175.11896	88.06312	59.04450	1

annotated MSMS spectrum

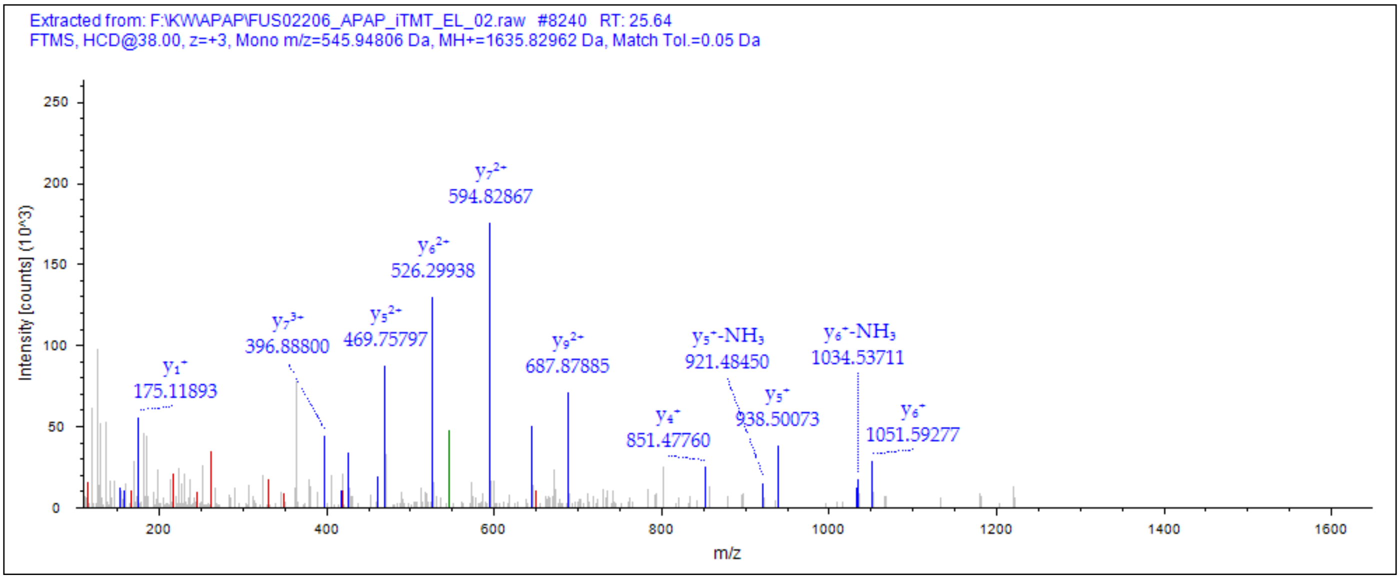

Sequence: LVILANNCPALR, C8-TMT6-Cys (329.22660 Da)
Charge: +3, Monoisotopic m/z: 542.66260 Da (+0.31 mmu/+0.57 ppm), MH+: 1625.97324 Da, RT: 45.81 min,
Identified with: Mascot (v1.30); IonScore:32, Exp Value:3.4E-003, Ions matched by search engine: 7/112
Fragment match tolerance used for search: 0.05 Da

b and y ion series

#1	a^+	a^{2+}	a^{3+}	b^+	b^{2+}	b^{3+}	Seq.	y^+	y^{2+}	y^{3+}	#2
1	86.09643	43.55185	29.37033	114.09135	57.54931	38.70197	L				12
2	185.16485	93.08606	62.39314	213.15977	107.08352	71.72477	V	1512.88825	756.94776	504.96760	11
3	298.24892	149.62810	100.08783	326.24384	163.62556	109.41946	I	1413.81983	707.41355	471.94479	10
4	411.33299	206.17013	137.78252	439.32791	220.16759	147.11415	L	1300.73576	650.87152	434.25010	9
5	482.37011	241.68869	161.46156	510.36503	255.68615	170.79319	A	1187.65169	594.32948	396.55541	8
6	596.41304	298.71016	199.47587	624.40796	312.70762	208.80750	N	1116.61457	558.81092	372.87637	7
7	710.45597	355.73162	237.49018	738.45089	369.72908	246.82181	N	1002.57164	501.78946	334.86206	6
8	1142.69176	571.84952	381.56877	1170.68667	585.84697	390.90041	C-TMT6-	888.52871	444.76799	296.84775	5
9	1239.74453	620.37590	413.91969	1267.73944	634.37336	423.25133	P	456.29292	228.65010	152.76916	4
10	1310.78165	655.89446	437.59873	1338.77656	669.89192	446.93037	A	359.24015	180.12371	120.41823	3
11	1423.86572	712.43650	475.29342	1451.86063	726.43395	484.62506	L	288.20303	144.60515	96.73919	2
12							R	175.11896	88.06312	59.04450	1

annotated MSMS spectrum

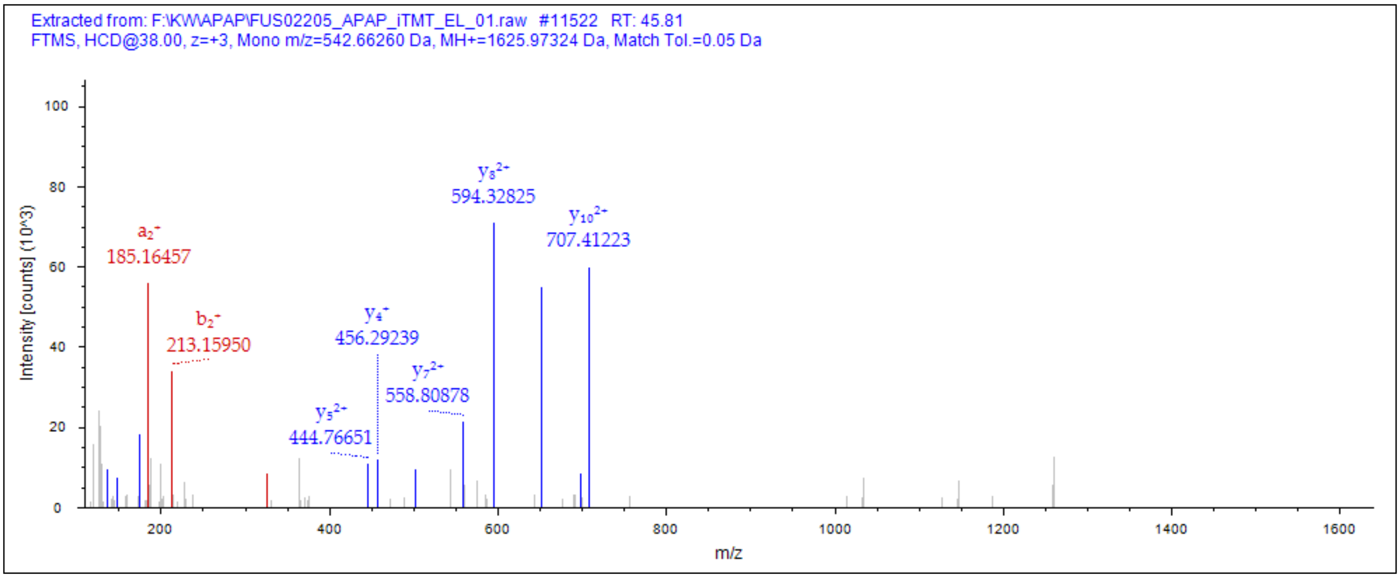

Sequence: ILDDWGETCK, C9-TMT6-Cys (329.22660 Da)
Charge: +2, Monoisotopic m/z: 754.88568 Da (+1.19 mmu/+1.58 ppm), MH+: 1508.76409 Da, RT: 38.86 min,
Identified with: Mascot (v1.30); IonScore:26, Exp Value:9.7E-002, Ions matched by search engine: 7/72
Fragment match tolerance used for search: 0.05 Da

b and y ion series

#1	a⁺	a²⁺	b⁺	b²⁺	Seq.	y⁺	y²⁺	#2
1	86.09643	43.55185	114.09135	57.54931	I			10
2	199.18050	100.09389	227.17542	114.09135	L	1395.67764	698.34246	9
3	314.20745	157.60736	342.20237	171.60482	D	1282.59357	641.80042	8
4	429.23440	215.12084	457.22932	229.11830	D	1167.56662	584.28695	7
5	615.31372	308.16050	643.30864	322.15796	W	1052.53967	526.77347	6
6	672.33519	336.67123	700.33011	350.66869	G	866.46035	433.73381	5
7	801.37779	401.19253	829.37271	415.18999	E	809.43888	405.22308	4
8	902.42547	451.71637	930.42039	465.71383	T	680.39628	340.70178	3
9	1334.66126	667.83427	1362.65617	681.83172	C-TMT6-	579.34860	290.17794	2
10					K	147.11281	74.06004	1

annotated MSMS spectrum

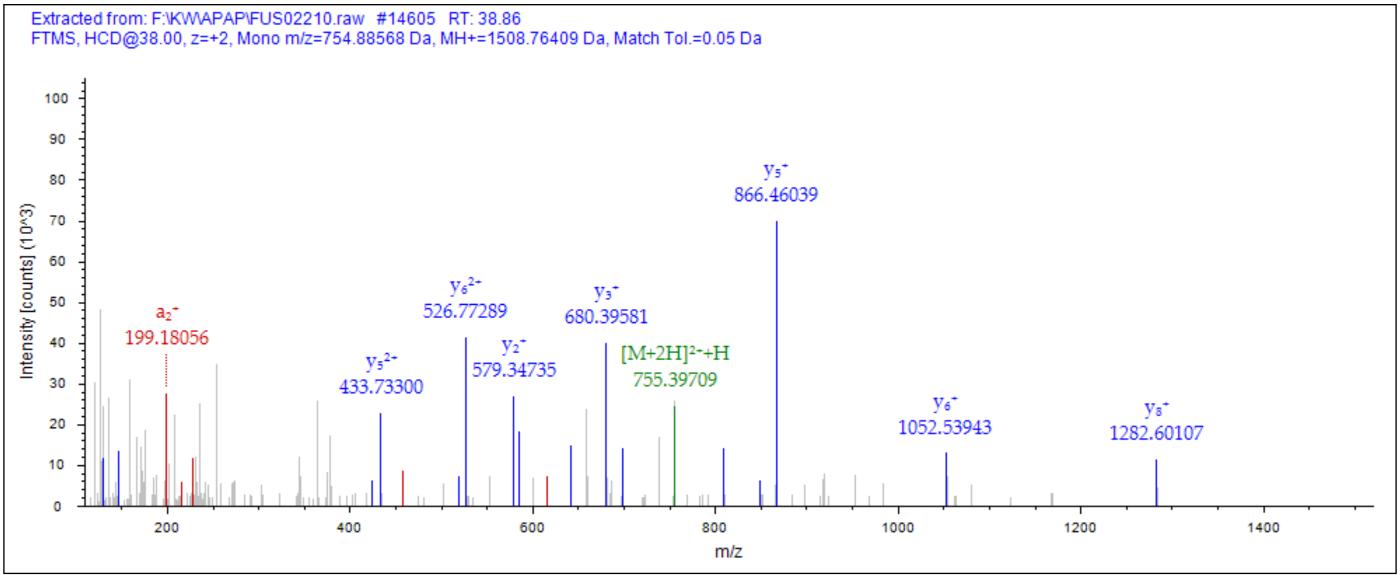

Sequence: DVAWAPSIGLPTSTIASCSQDGR, C18-TMT6-Cys (329.22660 Da)
Charge: +3,   Monoisotopic m/z: 887.78809 Da (-0.26 mmu/-0.3 ppm),   MH+: 2661.34970 Da,   RT: 74.60 min,
Identified with: Mascot (v1.30); IonScore:42, Exp Value:2.2E-003, Ions matched by search engine: 23/188
Fragment match tolerance used for search: 0.05 Da

b and y ion series

#1	a^+	a^{2+}	a^{3+}	b^+	b^{2+}	b^{3+}	Seq.	y^+	y^{2+}	y^{3+}	#2
1	88.03931	44.52329	30.01796	116.03423	58.52075	39.34959	D				23
2	187.10773	94.05750	63.04076	215.10265	108.05496	72.37240	V	2546.32355	1273.66541	849.44603	22
3	258.14485	129.57606	86.71980	286.13977	143.57352	96.05144	A	2447.25513	1224.13120	816.42323	21
4	444.22417	222.61572	148.74624	472.21909	236.61318	158.07788	W	2376.21801	1188.61264	792.74419	20
5	515.26129	258.13428	172.42528	543.25621	272.13174	181.75692	A	2190.13869	1095.57298	730.71775	19
6	612.31406	306.66067	204.77621	640.30898	320.65813	214.10784	P	2119.10157	1060.05442	707.03871	18
7	699.34609	350.17668	233.78688	727.34101	364.17414	243.11852	S	2022.04880	1011.52804	674.68778	17
8	812.43016	406.71872	271.48157	840.42508	420.71618	280.81321	I	1935.01677	968.01202	645.67711	16
9	869.45163	435.22945	290.48873	897.44655	449.22691	299.82037	G	1821.93270	911.46999	607.98242	15
10	982.53570	491.77149	328.18342	1010.53062	505.76895	337.51506	L	1764.91123	882.95925	588.97526	14
11	1079.58847	540.29787	360.53434	1107.58339	554.29533	369.86598	P	1651.82716	826.41722	551.28057	13
12	1180.63615	590.82171	394.21690	1208.63107	604.81917	403.54854	T	1554.77439	777.89083	518.92965	12
13	1267.66818	634.33773	423.22758	1295.66310	648.33519	432.55922	S	1453.72671	727.36699	485.24709	11
14	1368.71586	684.86157	456.91014	1396.71078	698.85903	466.24178	T	1366.69468	683.85098	456.23641	10
15	1481.79993	741.40360	494.60483	1509.79485	755.40106	503.93647	I	1265.64700	633.32714	422.55385	9
16	1552.83705	776.92216	518.28387	1580.83197	790.91962	527.61551	A	1152.56293	576.78510	384.85916	8
17	1639.86908	820.43818	547.29455	1667.86400	834.43564	556.62618	S	1081.52581	541.26654	361.18012	7
18	2072.10487	1036.55607	691.37314	2100.09978	1050.55353	700.70478	C-TMT6-	994.49378	497.75053	332.16944	6
19	2159.13690	1080.07209	720.38382	2187.13181	1094.06954	729.71545	S	562.25799	281.63263	188.09085	5
20	2287.19548	1144.10138	763.07001	2315.19039	1158.09883	772.40165	Q	475.22596	238.11662	159.08017	4
21	2402.22243	1201.61485	801.41233	2430.21734	1215.61231	810.74396	D	347.16738	174.08733	116.39398	3
22	2459.24390	1230.12559	820.41948	2487.23881	1244.12304	829.75112	G	232.14043	116.57385	78.05166	2
23							R	175.11896	88.06312	59.04450	1

annotated MSMS spectrum

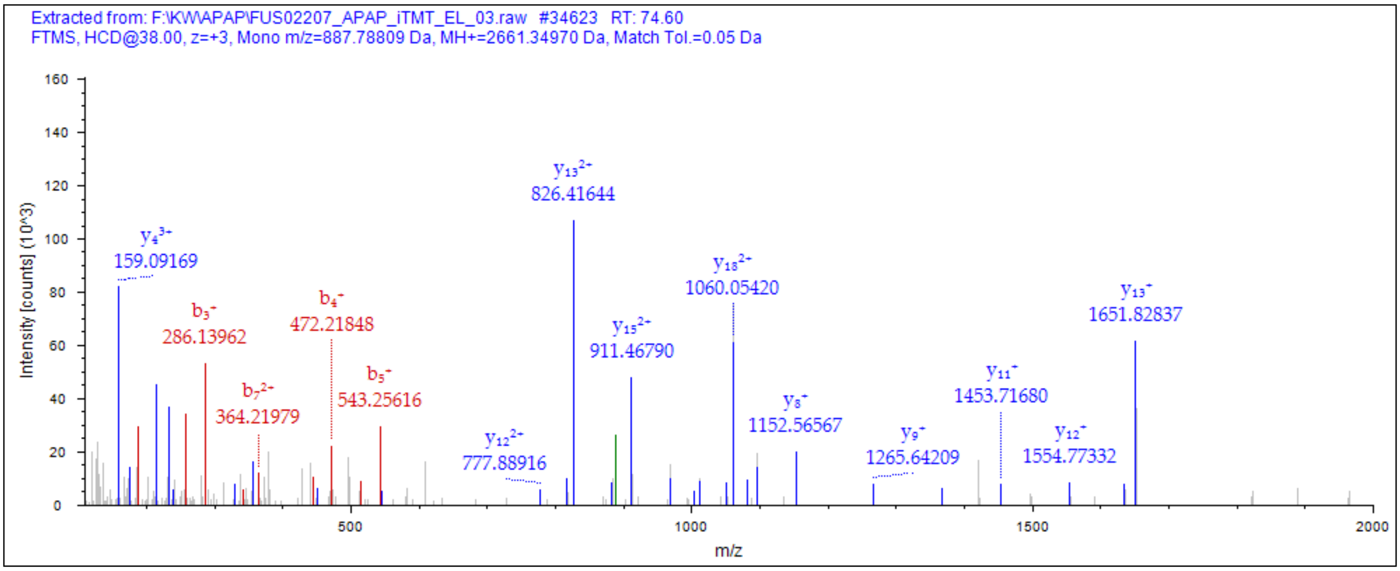

Sequence: YQTVIADICR, C9-TMT6-Cys (329.22660 Da)
Charge: +2, Monoisotopic m/z: 755.91711 Da (+1 mmu/+1.32 ppm), MH+: 1510.82695 Da, RT: 40.73 min,
Identified with: Mascot (v1.30); IonScore:27, Exp Value:4.5E-002, Ions matched by search engine: 5/104
Fragment match tolerance used for search: 0.05 Da

b and y ion series

#1	a^+	a^{2+}	b^+	b^{2+}	Seq.	y^+	y^{2+}	#2
1	136.07568	68.54148	164.07060	82.53894	Y			10
2	264.13426	132.57077	292.12918	146.56823	Q	1347.76164	674.38446	9
3	365.18194	183.09461	393.17686	197.09207	T	1219.70306	610.35517	8
4	464.25036	232.62882	492.24528	246.62628	V	1118.65538	559.83133	7
5	577.33443	289.17085	605.32935	303.16831	I	1019.58696	510.29712	6
6	648.37155	324.68941	676.36647	338.68687	A	906.50289	453.75508	5
7	763.39850	382.20289	791.39342	396.20035	D	835.46577	418.23652	4
8	876.48257	438.74492	904.47749	452.74238	I	720.43882	360.72305	3
9	1308.71836	654.86282	1336.71327	668.86027	C-TMT6-	607.35475	304.18101	2
10					R	175.11896	88.06312	1

annotated MSMS spectrum

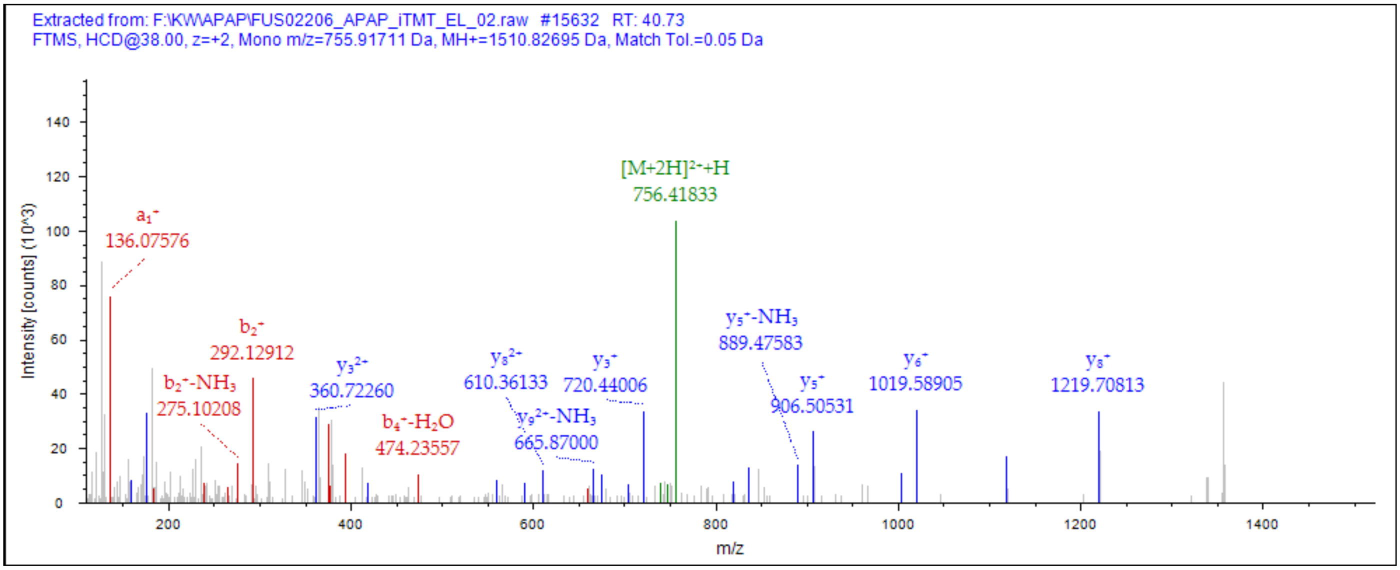

Sequence: NLALCPANHAPLR, C5-TMT6-Cys (329.22660 Da)
Charge: +4, Monoisotopic m/z: 430.49725 Da (-0.36 mmu/-0.84 ppm), MH+: 1718.96718 Da, RT: 26.81 min,
Identified with: Mascot (v1.30); IonScore:22, Exp Value:1.2E-001, Ions matched by search engine: 7/144
Fragment match tolerance used for search: 0.05 Da

b and y ion series

#1	a^+	a^{2+}	a^{3+}	a^{4+}	b^+	b^{2+}	b^{3+}	b^{4+}	Seq.	y^+	y^{2+}	y^{3+}	y^{4+}	#2
1	87.05529	44.03128	29.68995	22.51928	115.05021	58.02874	39.02159	29.51801	N					13
2	200.13936	100.57332	67.38464	50.79030	228.13428	114.57078	76.71628	57.78903	L	1604.92570	802.96649	535.64675	401.98688	12
3	271.17648	136.09188	91.06368	68.54958	299.17140	150.08934	100.39532	75.54831	A	1491.84163	746.42445	497.95206	373.71586	11
4	384.26055	192.63391	128.75837	96.82060	412.25547	206.63137	138.09001	103.81932	L	1420.80451	710.90589	474.27302	355.95658	10
5	816.49634	408.75181	272.83696	204.87954	844.49125	422.74926	282.16860	211.87827	C-TMT6-	1307.72044	654.36386	436.57833	327.68557	9
6	913.54911	457.27819	305.18789	229.14273	941.54402	471.27565	314.51952	236.14146	P	875.48465	438.24596	292.49973	219.62662	8
7	984.58623	492.79675	328.86693	246.90201	1012.58114	506.79421	338.19856	253.90074	A	778.43188	389.71958	260.14881	195.36343	7
8	1098.62916	549.81822	366.88124	275.41275	1126.62407	563.81567	376.21287	282.41148	N	707.39476	354.20102	236.46977	177.60415	6
9	1235.68807	618.34767	412.56754	309.67747	1263.68298	632.34513	421.89918	316.67620	H	593.35183	297.17955	198.45546	149.09342	5
10	1306.72519	653.86623	436.24658	327.43675	1334.72010	667.86369	445.57822	334.43548	A	456.29292	228.65010	152.76916	114.82869	4
11	1403.77796	702.39262	468.59750	351.69995	1431.77287	716.39007	477.92914	358.69868	P	385.25580	193.13154	129.09012	97.06941	3
12	1516.86203	758.93465	506.29219	379.97096	1544.85694	772.93211	515.62383	386.96969	L	288.20303	144.60515	96.73919	72.80622	2
13									R	175.11896	88.06312	59.04450	44.53520	1

annotated MSMS spectrum

Extracted from: F:\KW\APAP\FUS02207_APAP_iTMT_EL_03.raw #8990 RT: 26.81
FTMS, HCD@38.00, z=+4, Mono m/z=430.49725 Da, MH+=1718.96718 Da, Match Tol.=0.05 Da

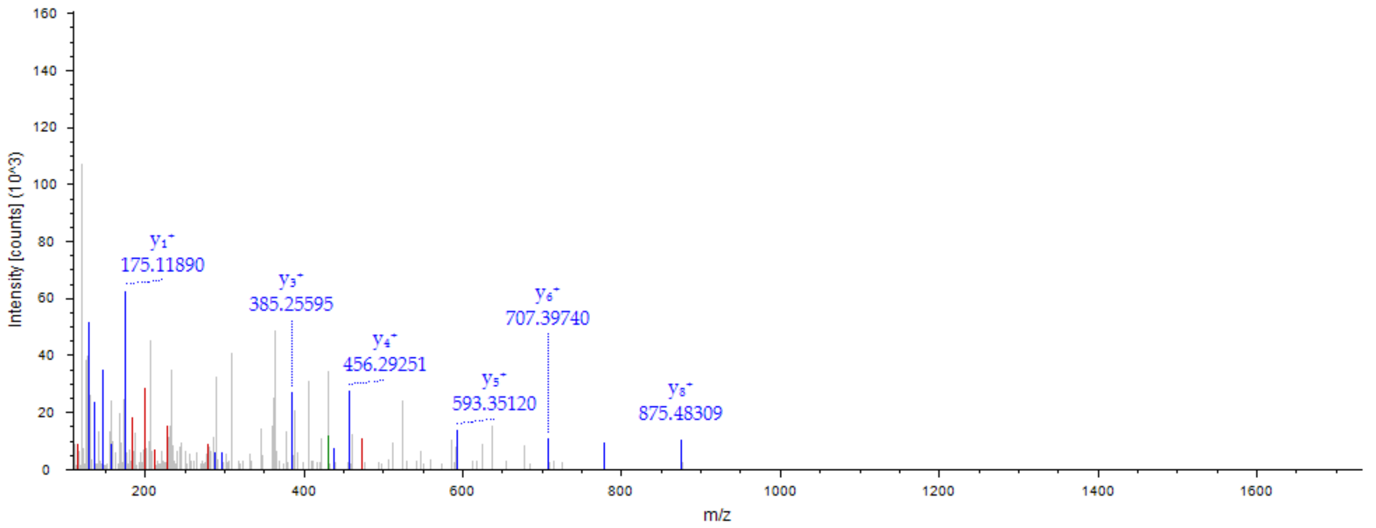

Sequence: GLLDVTCK, C7-TMT6-Cys (329.22660 Da)
Charge: +3, Monoisotopic m/z: 393.23221 Da (+0.27 mmu/+0.69 ppm), MH+: 1177.68207 Da, RT: 38.41 min,
Identified with: Mascot (v1.30); IonScore:30, Exp Value:1.2E-002, Ions matched by search engine: 4/56
Fragment match tolerance used for search: 0.05 Da

b and y ion series

#1	a^+	a^{2+}	a^{3+}	b^+	b^{2+}	b^{3+}	Seq.	y^+	y^{2+}	y^{3+}	#2
1	30.03383	15.52055	10.68280	58.02875	29.51801	20.01443	G				8
2	143.11790	72.06259	48.37749	171.11282	86.06005	57.70912	L	1120.65979	560.83353	374.22478	7
3	256.20197	128.60462	86.07218	284.19689	142.60208	95.40381	L	1007.57572	504.29150	336.53009	6
4	371.22892	186.11810	124.41449	399.22384	200.11556	133.74613	D	894.49165	447.74946	298.83540	5
5	470.29734	235.65231	157.43730	498.29226	249.64977	166.76894	V	779.46470	390.23599	260.49308	4
6	571.34502	286.17615	191.11986	599.33994	300.17361	200.45150	T	680.39628	340.70178	227.47028	3
7	1003.58081	502.29404	335.19845	1031.57572	516.29150	344.53009	C-TMT6-	579.34860	290.17794	193.78772	2
8							K	147.11281	74.06004	49.70912	1

annotated MSMS spectrum

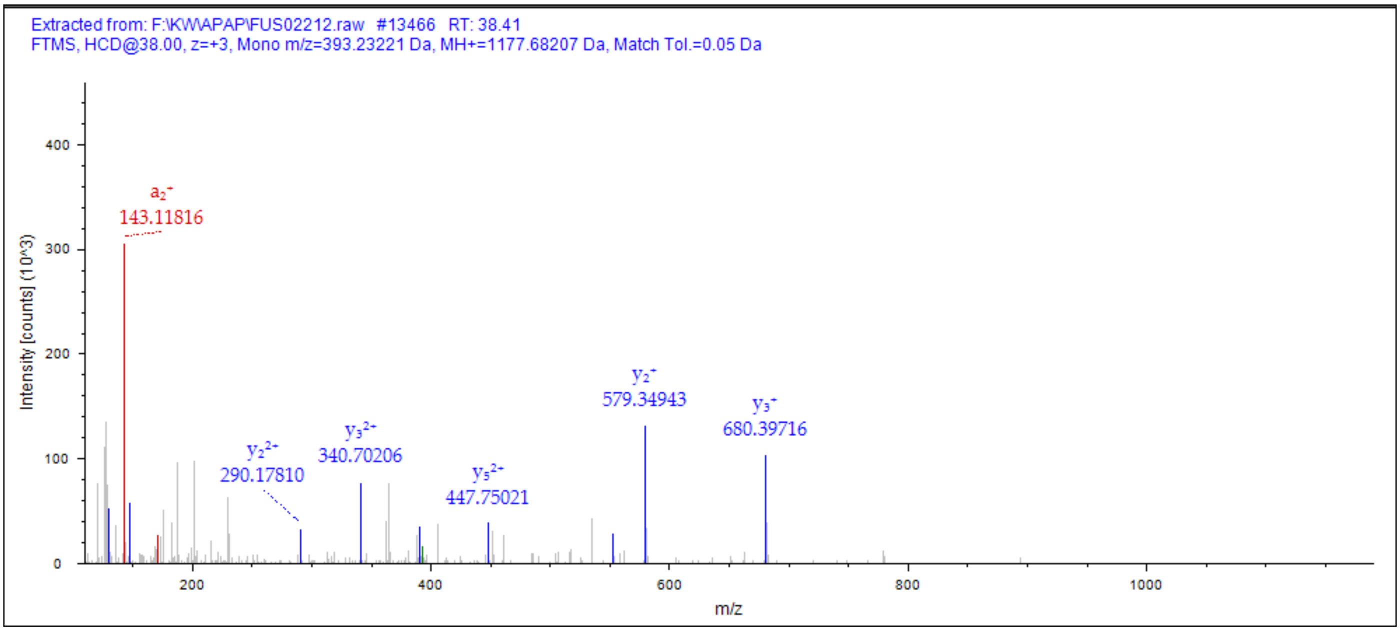

Sequence: FSPNSSNPIIVSCGWDK, C13-TMT6-Cys (329.22660 Da)
Charge: +3, Monoisotopic m/z: 727.37152 Da (-0.28 mmu/-0.38 ppm), MH+: 2180.10001 Da, RT: 54.45 min,
Identified with: Mascot (v1.30); IonScore:40, Exp Value:3.7E-003, Ions matched by search engine: 10/180
Fragment match tolerance used for search: 0.05 Da

b and y ion series

#1	a^+	a^{2+}	a^{3+}	b^+	b^{2+}	b^{3+}	Seq.	y^+	y^{2+}	y^{3+}	#2
1	120.08078	60.54403	40.69845	148.07570	74.54149	50.03008	F				17
2	207.11281	104.06004	69.70912	235.10773	118.05750	79.04076	S	2033.03242	1017.01985	678.34899	16
3	304.16558	152.58643	102.06005	332.16050	166.58389	111.39168	P	1946.00039	973.50383	649.33831	15
4	418.20851	209.60789	140.07436	446.20343	223.60535	149.40599	N	1848.94762	924.97745	616.98739	14
5	505.24054	253.12391	169.08503	533.23546	267.12137	178.41667	S	1734.90469	867.95598	578.97308	13
6	592.27257	296.63992	198.09571	620.26749	310.63738	207.42735	S	1647.87266	824.43997	549.96240	12
7	706.31550	353.66139	236.11002	734.31042	367.65885	245.44166	N	1560.84063	780.92395	520.95173	11
8	803.36827	402.18777	268.46094	831.36319	416.18523	277.79258	P	1446.79770	723.90249	482.93742	10
9	916.45234	458.72981	306.15563	944.44726	472.72727	315.48727	I	1349.74493	675.37610	450.58649	9
10	1029.53641	515.27184	343.85032	1057.53133	529.26930	353.18196	I	1236.66086	618.83407	412.89180	8
11	1128.60483	564.80605	376.87313	1156.59975	578.80351	386.20477	V	1123.57679	562.29203	375.19711	7
12	1215.63686	608.32207	405.88381	1243.63178	622.31953	415.21544	S	1024.50837	512.75782	342.17431	6
13	1647.87265	824.43996	549.96240	1675.86756	838.43742	559.29404	C-TMT6-	937.47634	469.24181	313.16363	5
14	1704.89412	852.95070	568.96956	1732.88903	866.94815	578.30119	G	505.24055	253.12391	169.08503	4
15	1890.97344	945.99036	630.99600	1918.96835	959.98781	640.32763	W	448.21908	224.61318	150.07788	3
16	2006.00039	1003.50383	669.33831	2033.99530	1017.50129	678.66995	D	262.13976	131.57352	88.05144	2
17							K	147.11281	74.06004	49.70912	1

annotated MSMS spectrum

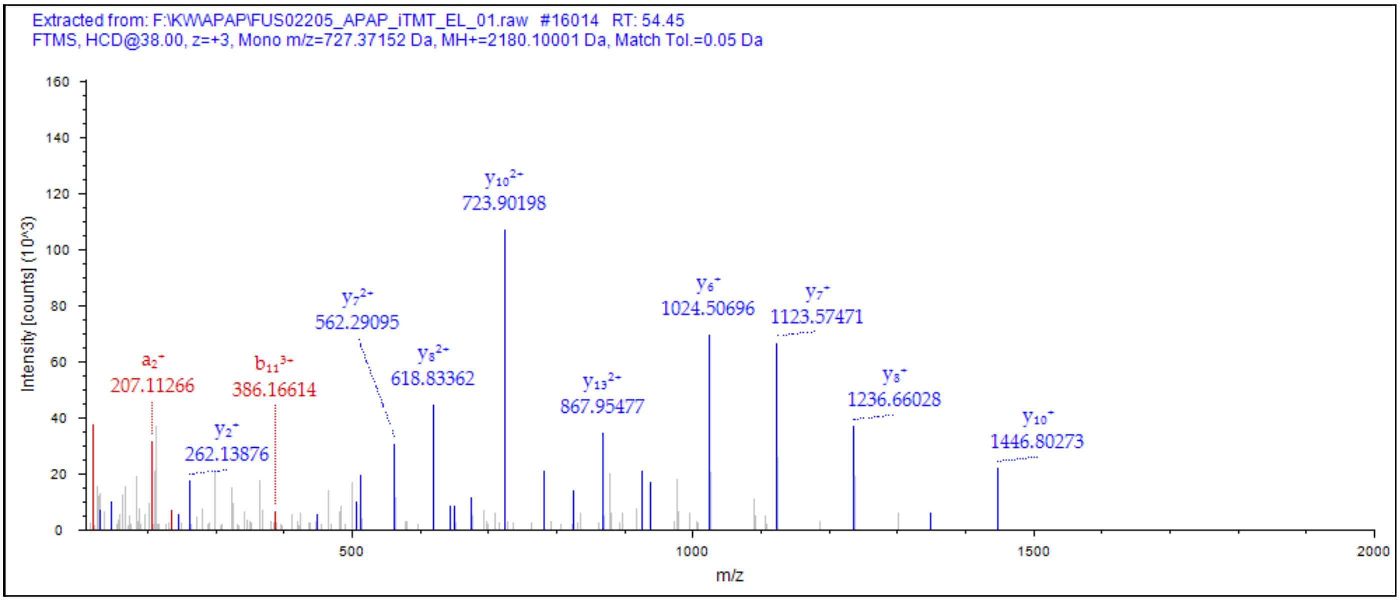

Sequence: TYIQCIAAISR, C5-TMT6-Cys (329.22660 Da)
Charge: +3, Monoisotopic m/z: 523.29950 Da (+0.38 mmu/+0.73 ppm), MH+: 1567.88395 Da, RT: 47.05 min,
Identified with: Mascot (v1.30); IonScore:16, Exp Value:4.3E-001, Ions matched by search engine: 9/108
Fragment match tolerance used for search: 0.05 Da

b and y ion series

#1	a^+	a^{2+}	a^{3+}	b^+	b^{2+}	b^{3+}	Seq.	y^+	y^{2+}	y^{3+}	#2
1	74.06004	37.53366	25.35820	102.05496	51.53112	34.68984	T				11
2	237.12336	119.06532	79.71264	265.11828	133.06278	89.04428	Y	1466.83513	733.92120	489.61656	10
3	350.20743	175.60735	117.40733	378.20235	189.60481	126.73897	I	1303.77181	652.38954	435.26212	9
4	478.26601	239.63664	160.09352	506.26093	253.63410	169.42516	Q	1190.68774	595.84751	397.56743	8
5	910.50180	455.75454	304.17212	938.49671	469.75199	313.50375	C-TMT6-	1062.62916	531.81822	354.88124	7
6	1023.58587	512.29657	341.86681	1051.58078	526.29403	351.19844	I	630.39337	315.70032	210.80264	6
7	1094.62299	547.81513	365.54585	1122.61790	561.81259	374.87748	A	517.30930	259.15829	173.10795	5
8	1165.66011	583.33369	389.22489	1193.65502	597.33115	398.55652	A	446.27218	223.63973	149.42891	4
9	1278.74418	639.87573	426.91958	1306.73909	653.87318	436.25121	I	375.23506	188.12117	125.74987	3
10	1365.77621	683.39174	455.93025	1393.77112	697.38920	465.26189	S	262.15099	131.57913	88.05518	2
11							R	175.11896	88.06312	59.04450	1

annotated MSMS spectrum

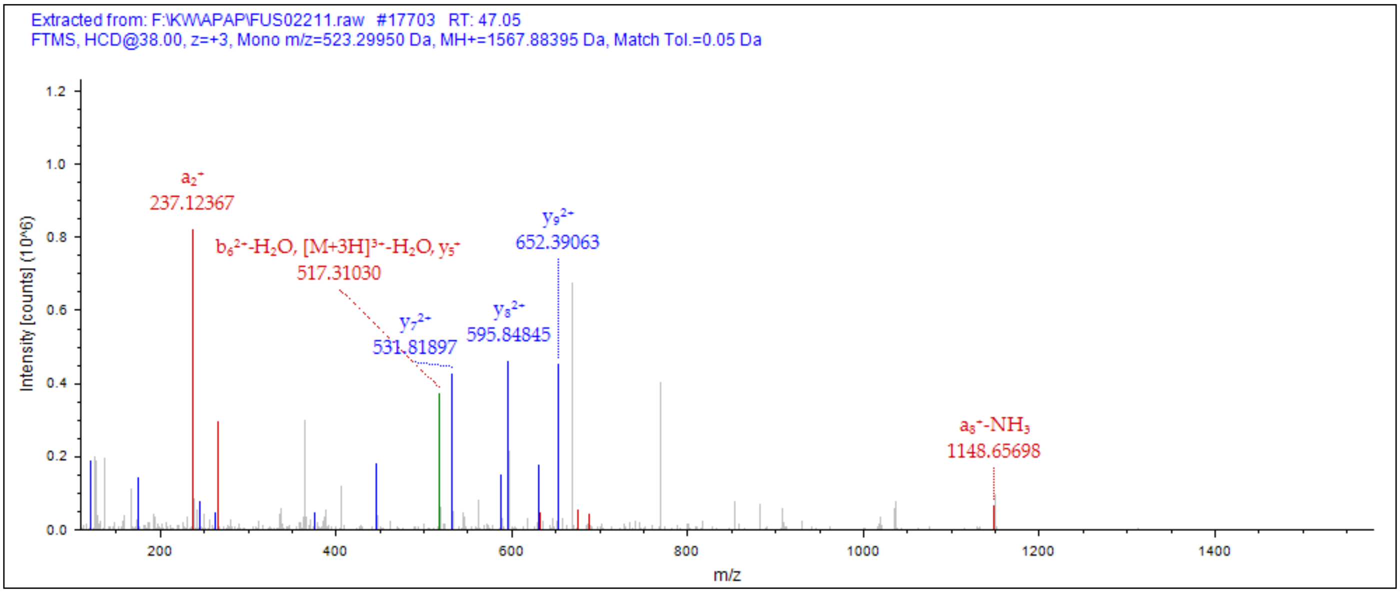

Sequence: TDTVLILCR, C8-TMT6-Cys (329.22660 Da)
Charge: +2, Monoisotopic m/z: 681.90369 Da (+1.21 mmu/+1.77 ppm), MH+: 1362.80010 Da, RT: 44.85 min,
Identified with: Mascot (v1.30); IonScore:26, Exp Value:2.8E-002, Ions matched by search engine: 5/64
Fragment match tolerance used for search: 0.05 Da

b and y ion series

#1	a⁺	a²⁺	b⁺	b²⁺	Seq.	y⁺	y²⁺	#2
1	74.06004	37.53366	102.05496	51.53112	T			9
2	189.08699	95.04713	217.08191	109.04459	D	1261.75001	631.37864	8
3	290.13467	145.57097	318.12959	159.56843	T	1146.72306	573.86517	7
4	389.20309	195.10518	417.19801	209.10264	V	1045.67538	523.34133	6
5	502.28716	251.64722	530.28208	265.64468	L	946.60696	473.80712	5
6	615.37123	308.18925	643.36615	322.18671	I	833.52289	417.26508	4
7	728.45530	364.73129	756.45022	378.72875	L	720.43882	360.72305	3
8	1160.69109	580.84918	1188.68600	594.84664	C-TMT6-	607.35475	304.18101	2
9					R	175.11896	88.06312	1

annotated MSMS spectrum

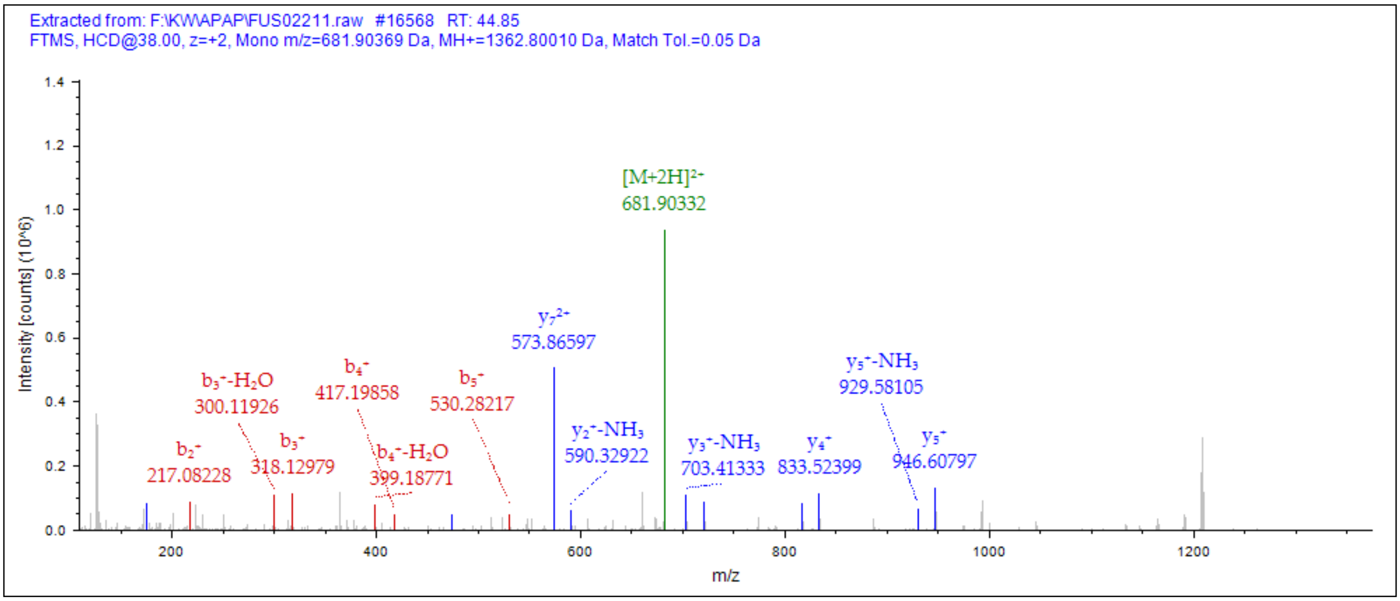

Sequence: VLAALPAAELVQACR, C14-TMT6-Cys (329.22660 Da)
Charge: +3, Monoisotopic m/z: 618.69958 Da (+0.29 mmu/+0.46 ppm), MH+: 1854.08420 Da, RT: 60.87 min,
Identified with: Mascot (v1.30); IonScore:37, Exp Value:1.5E-003, Ions matched by search engine: 17/124
Fragment match tolerance used for search: 0.05 Da

b and y ion series

#1	a⁺	a²⁺	a³⁺	b⁺	b²⁺	b³⁺	Seq.	y⁺	y²⁺	y³⁺	#2
1	72.08078	36.54403	24.69845	100.07570	50.54149	34.03008	V				15
2	185.16485	93.08606	62.39314	213.15977	107.08352	71.72477	L	1755.01493	878.01110	585.67649	14
3	256.20197	128.60462	86.07218	284.19689	142.60208	95.40381	A	1641.93086	821.46907	547.98180	13
4	327.23909	164.12318	109.75122	355.23401	178.12064	119.08285	A	1570.89374	785.95051	524.30276	12
5	440.32316	220.66522	147.44591	468.31808	234.66268	156.77754	L	1499.85662	750.43195	500.62372	11
6	537.37593	269.19160	179.79683	565.37085	283.18906	189.12847	P	1386.77255	693.88991	462.92903	10
7	608.41305	304.71016	203.47587	636.40797	318.70762	212.80751	A	1289.71978	645.36353	430.57811	9
8	679.45017	340.22872	227.15491	707.44509	354.22618	236.48655	A	1218.68266	609.84497	406.89907	8
9	808.49277	404.75002	270.16911	836.48769	418.74748	279.50075	E	1147.64554	574.32641	383.22003	7
10	921.57684	461.29206	307.86380	949.57176	475.28952	317.19544	L	1018.60294	509.80511	340.20583	6
11	1020.64526	510.82627	340.88661	1048.64018	524.82373	350.21824	V	905.51887	453.26307	302.51114	5
12	1148.70384	574.85556	383.57280	1176.69876	588.85302	392.90444	Q	806.45045	403.72886	269.48833	4
13	1219.74096	610.37412	407.25184	1247.73588	624.37158	416.58348	A	678.39187	339.69957	226.80214	3
14	1651.97675	826.49201	551.33043	1679.97166	840.48947	560.66207	C-TMT6-	607.35475	304.18101	203.12310	2
15							R	175.11896	88.06312	59.04450	1

annotated MSMS spectrum

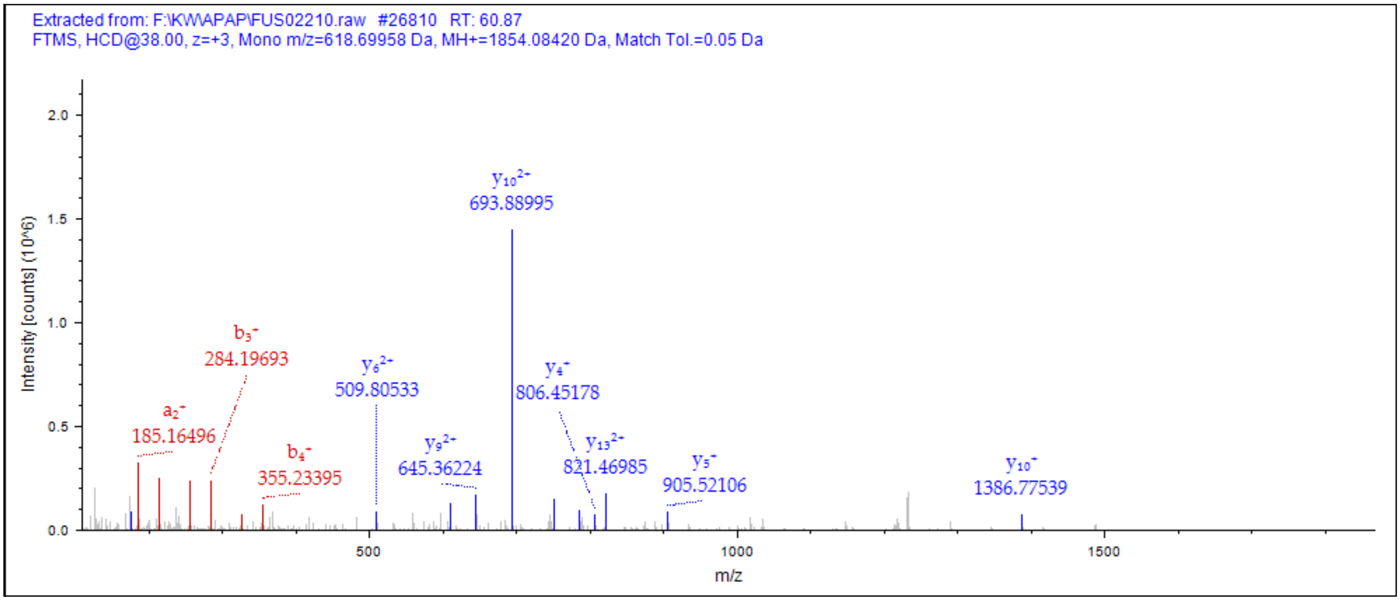

Sequence: ALANSLACQGK, C8-TMT6-Cys (329.22660 Da)
Charge: +2, Monoisotopic m/z: 702.89587 Da (+0.68 mmu/+0.97 ppm), MH+: 1404.78447 Da, RT: 19.75 min,
Identified with: Mascot (v1.30); IonScore:44, Exp Value:9.0E-004, Ions matched by search engine: 6/108
Fragment match tolerance used for search: 0.05 Da

b and y ion series

#1	a^+	a^{2+}	b^+	b^{2+}	Seq.	y^+	y^{2+}	#2
1	44.04948	22.52838	72.04440	36.52584	A			11
2	157.13355	79.07041	185.12847	93.06787	L	1333.74599	667.37663	10
3	228.17067	114.58897	256.16559	128.58643	A	1220.66192	610.83460	9
4	342.21360	171.61044	370.20852	185.60790	N	1149.62480	575.31604	8
5	429.24563	215.12645	457.24055	229.12391	S	1035.58187	518.29457	7
6	542.32970	271.66849	570.32462	285.66595	L	948.54984	474.77856	6
7	613.36682	307.18705	641.36174	321.18451	A	835.46577	418.23652	5
8	1045.60261	523.30494	1073.59752	537.30240	C-TMT6-	764.42865	382.71796	4
9	1173.66119	587.33423	1201.65610	601.33169	Q	332.19286	166.60007	3
10	1230.68266	615.84497	1258.67757	629.84242	G	204.13428	102.57078	2
11					K	147.11281	74.06004	1

annotated MSMS spectrum

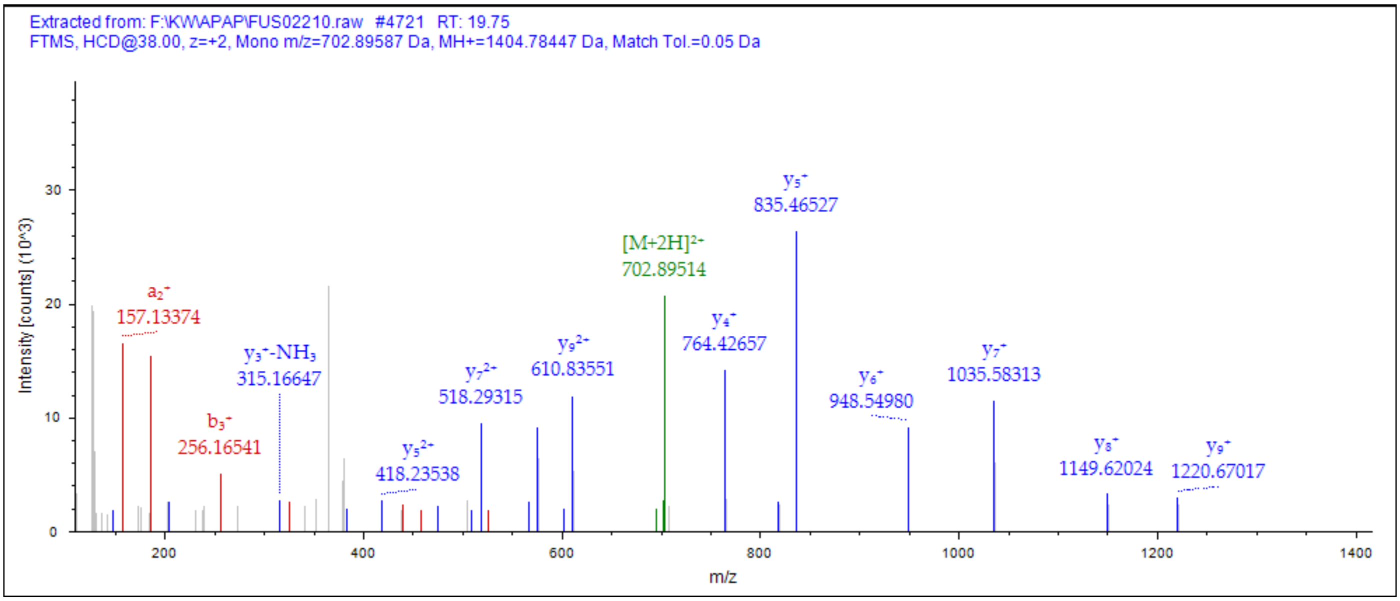

Sequence: GIFPVLCK, C7-TMT6-Cys (329.22660 Da)
Charge: +2, Monoisotopic m/z: 603.36731 Da (-0.24 mmu/-0.39 ppm), MH+: 1205.72734 Da, RT: 50.39 min,
Identified with: Mascot (v1.30); IonScore:35, Exp Value:2.5E-003, Ions matched by search engine: 5/56
Fragment match tolerance used for search: 0.05 Da

b and y ion series

#1	a⁺	a²⁺	b⁺	b²⁺	Seq.	y⁺	y²⁺	#2
1	30.03383	15.52055	58.02875	29.51801	G			8
2	143.11790	72.06259	171.11282	86.06005	I	1148.70635	574.85681	7
3	290.18632	145.59680	318.18124	159.59426	F	1035.62228	518.31478	6
4	387.23909	194.12318	415.23401	208.12064	P	888.55386	444.78057	5
5	486.30751	243.65739	514.30243	257.65485	V	791.50109	396.25418	4
6	599.39158	300.19943	627.38650	314.19689	L	692.43267	346.71997	3
7	1031.62737	516.31732	1059.62228	530.31478	C-TMT6-	579.34860	290.17794	2
8					K	147.11281	74.06004	1

annotated MSMS spectrum

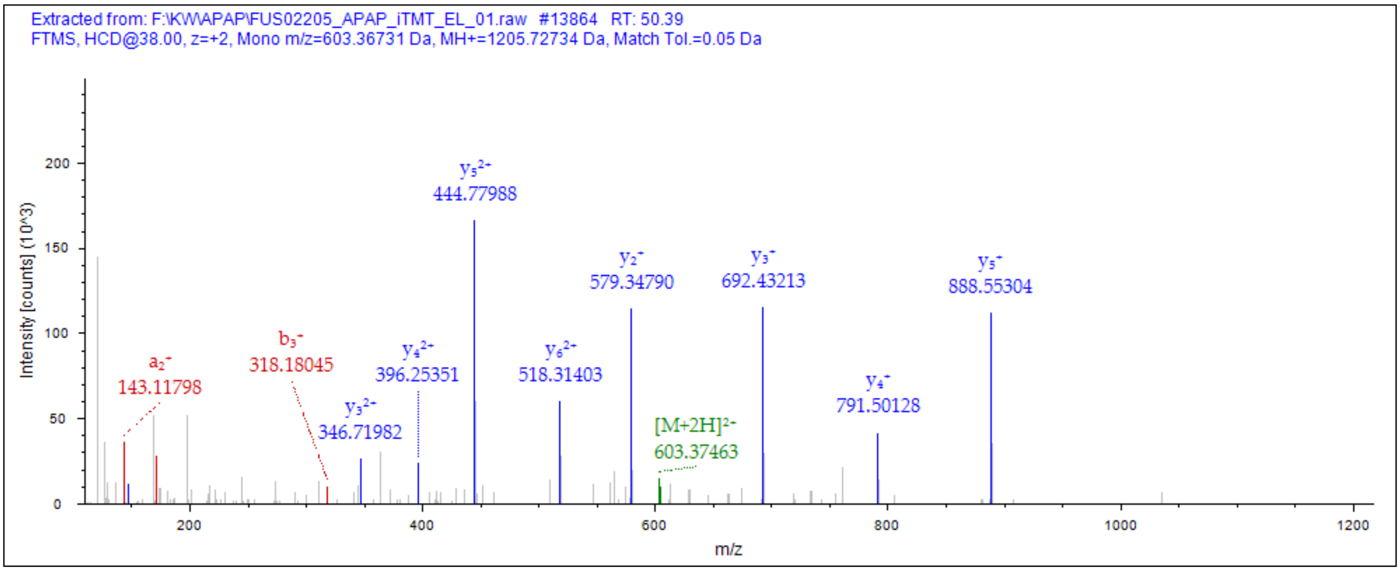

Sequence: IAVAAQNCYK, C8-TMT6-Cys (329.22660 Da)
Charge: +2, Monoisotopic m/z: 705.39386 Da (+1.58 mmu/+2.25 ppm), MH+: 1409.78044 Da, RT: 21.85 min,
Identified with: Mascot (v1.30); IonScore:53, Exp Value:1.0E-004, Ions matched by search engine: 6/88
Fragment match tolerance used for search: 0.05 Da

b and y ion series

#1	a^+	a^{2+}	b^+	b^{2+}	Seq.	y^+	y^{2+}	#2
1	86.09643	43.55185	114.09135	57.54931	I			10
2	157.13355	79.07041	185.12847	93.06787	A	1296.69321	648.85024	9
3	256.20197	128.60462	284.19689	142.60208	V	1225.65609	613.33168	8
4	327.23909	164.12318	355.23401	178.12064	A	1126.58767	563.79747	7
5	398.27621	199.64174	426.27113	213.63920	A	1055.55055	528.27891	6
6	526.33479	263.67103	554.32971	277.66849	Q	984.51343	492.76035	5
7	640.37772	320.69250	668.37264	334.68996	N	856.45485	428.73106	4
8	1072.61351	536.81039	1100.60842	550.80785	C-TMT6-	742.41192	371.70960	3
9	1235.67683	618.34205	1263.67174	632.33951	Y	310.17613	155.59170	2
10					K	147.11281	74.06004	1

annotated MSMS spectrum

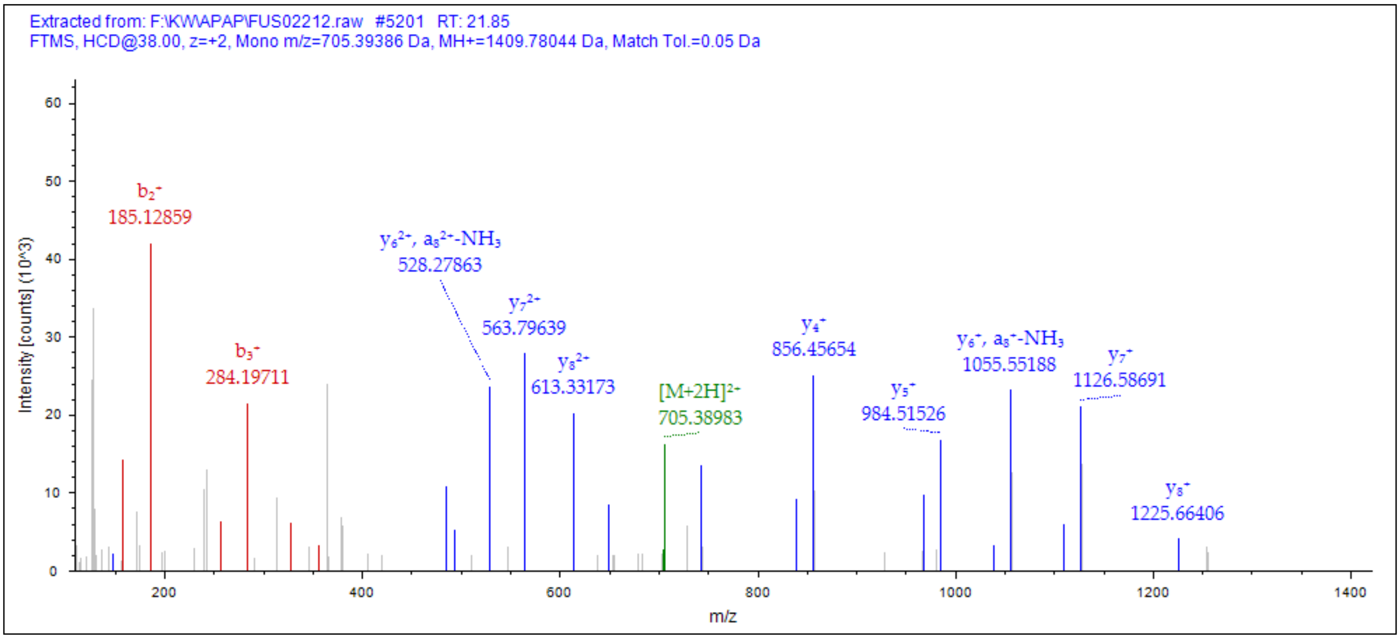

Sequence: DLSHIGDAVVISCAK, C13-TMT6-Cys (329.22660 Da)
Charge: +3, Monoisotopic m/z: 619.67554 Da (+0.61 mmu/+0.99 ppm), MH+: 1857.01206 Da, RT: 50.24 min,
Identified with: Mascot (v1.30); IonScore:45, Exp Value:7.7E-004, Ions matched by search engine: 8/112
Fragment match tolerance used for search: 0.05 Da

b and y ion series

#1	a^+	a^{2+}	a^{3+}	b^+	b^{2+}	b^{3+}	Seq.	y^+	y^{2+}	y^{3+}	#2
1	88.03931	44.52329	30.01796	116.03423	58.52075	39.34959	D				15
2	201.12338	101.06533	67.71265	229.11830	115.06279	77.04428	L	1741.98328	871.49528	581.33261	14
3	288.15541	144.58134	96.72332	316.15033	158.57880	106.05496	S	1628.89921	814.95324	543.63792	13
4	425.21432	213.11080	142.40963	453.20924	227.10826	151.74126	H	1541.86718	771.43723	514.62724	12
5	538.29839	269.65283	180.10432	566.29331	283.65029	189.43595	I	1404.80827	702.90777	468.94094	11
6	595.31986	298.16357	199.11147	623.31478	312.16103	208.44311	G	1291.72420	646.36574	431.24625	10
7	710.34681	355.67704	237.45379	738.34173	369.67450	246.78543	D	1234.70273	617.85500	412.23909	9
8	781.38393	391.19560	261.13283	809.37885	405.19306	270.46447	A	1119.67578	560.34153	373.89678	8
9	880.45235	440.72981	294.15564	908.44727	454.72727	303.48727	V	1048.63866	524.82297	350.21774	7
10	979.52077	490.26402	327.17844	1007.51569	504.26148	336.51008	V	949.57024	475.28876	317.19493	6
11	1092.60484	546.80606	364.87313	1120.59976	560.80352	374.20477	I	850.50182	425.75455	284.17212	5
12	1179.63687	590.32207	393.88381	1207.63179	604.31953	403.21545	S	737.41775	369.21251	246.47743	4
13	1611.87266	806.43997	537.96240	1639.86757	820.43742	547.29404	C-TMT6-	650.38572	325.69650	217.46676	3
14	1682.90978	841.95853	561.64144	1710.90469	855.95598	570.97308	A	218.14993	109.57860	73.38816	2
15							K	147.11281	74.06004	49.70912	1

annotated MSMS spectrum

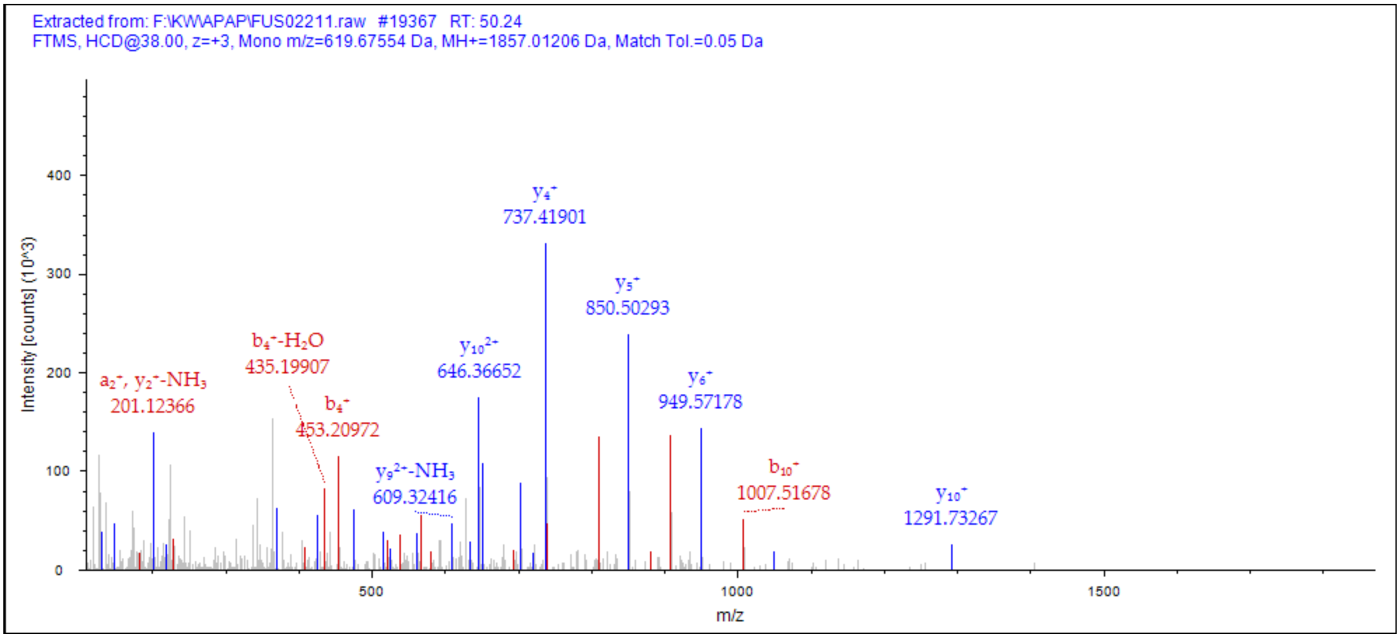

Sequence: SPGLQPVLCLR, C9-TMT6-Cys (329.22660 Da)
Charge: +3, Monoisotopic m/z: 504.63538 Da (-0.47 mmu/-0.94 ppm), MH+: 1511.89157 Da, RT: 48.12 min,
Identified with: Mascot (v1.30); IonScore:14, Exp Value:3.6E-001, Ions matched by search engine: 11/104
Fragment match tolerance used for search: 0.05 Da

b and y ion series

#1	a^+	a^{2+}	a^{3+}	b^+	b^{2+}	b^{3+}	Seq.	y^+	y^{2+}	y^{3+}	#2
1	60.04439	30.52583	20.68632	88.03931	44.52329	30.01795	S				11
2	157.09716	79.05222	53.03724	185.09208	93.04968	62.36888	P	1424.86097	712.93412	475.62517	10
3	214.11863	107.56295	72.04440	242.11355	121.56041	81.37603	G	1327.80820	664.40774	443.27425	9
4	327.20270	164.10499	109.73909	355.19762	178.10245	119.07072	L	1270.78673	635.89700	424.26709	8
5	455.26128	228.13428	152.42528	483.25620	242.13174	161.75692	Q	1157.70266	579.35497	386.57240	7
6	552.31405	276.66066	184.77620	580.30897	290.65812	194.10784	P	1029.64408	515.32568	343.88621	6
7	651.38247	326.19487	217.79901	679.37739	340.19233	227.13065	V	932.59131	466.79929	311.53529	5
8	764.46654	382.73691	255.49370	792.46146	396.73437	264.82534	L	833.52289	417.26508	278.51248	4
9	1196.70233	598.85480	399.57229	1224.69724	612.85226	408.90393	C-TMT6-	720.43882	360.72305	240.81779	3
10	1309.78640	655.39684	437.26698	1337.78131	669.39429	446.59862	L	288.20303	144.60515	96.73919	2
11							R	175.11896	88.06312	59.04450	1

annotated MSMS spectrum

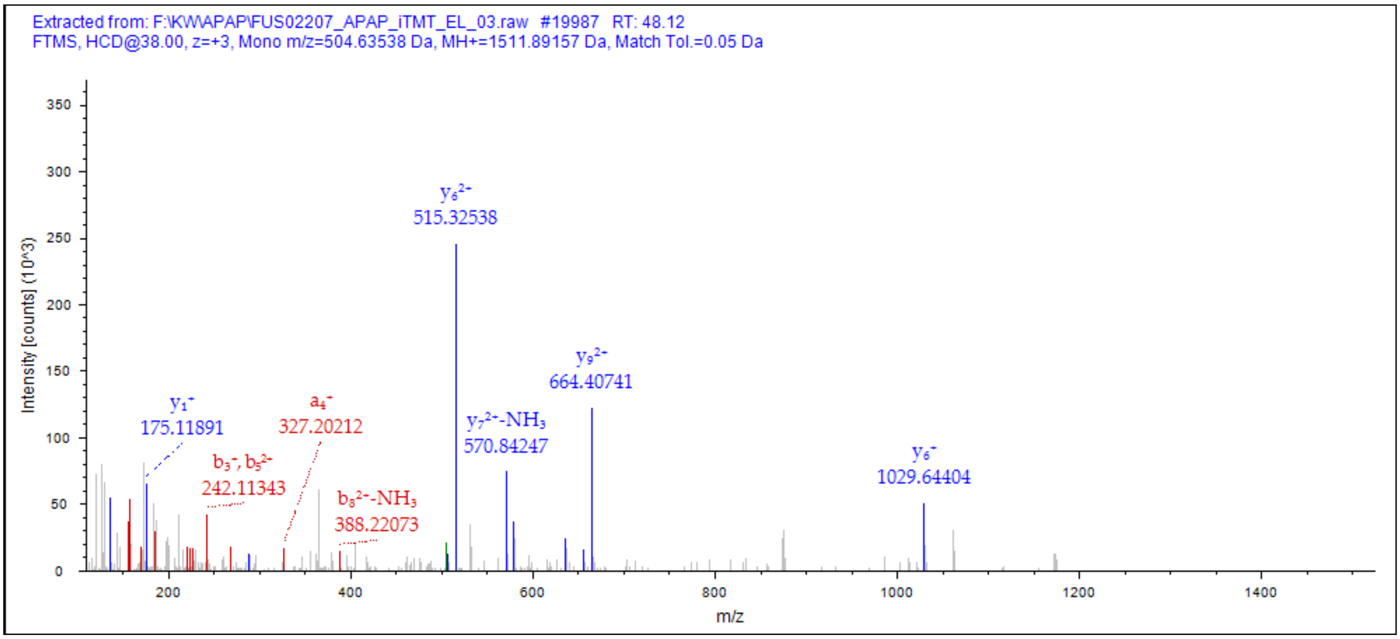

Sequence: TIVYLDGSSQSCR, C12-TMT6-Cys (329.22660 Da)
Charge: +2, Monoisotopic m/z: 879.45587 Da (-0.47 mmu/-0.53 ppm), MH+: 1757.90447 Da, RT: 31.82 min,
Identified with: Mascot (v1.30); IonScore:47, Exp Value:6.8E-004, Ions matched by search engine: 6/108
Fragment match tolerance used for search: 0.05 Da

b and y ion series

#1	a⁺	a²⁺	b⁺	b²⁺	Seq.	y⁺	y²⁺	#2
1	74.06004	37.53366	102.05496	51.53112	T			13
2	187.14411	94.07569	215.13903	108.07315	I	1656.85772	828.93250	12
3	286.21253	143.60990	314.20745	157.60736	V	1543.77365	772.39046	11
4	449.27585	225.14156	477.27077	239.13902	Y	1444.70523	722.85625	10
5	562.35992	281.68360	590.35484	295.68106	L	1281.64191	641.32459	9
6	677.38687	339.19707	705.38179	353.19453	D	1168.55784	584.78256	8
7	734.40834	367.70781	762.40326	381.70527	G	1053.53089	527.26908	7
8	821.44037	411.22382	849.43529	425.22128	S	996.50942	498.75835	6
9	908.47240	454.73984	936.46732	468.73730	S	909.47739	455.24233	5
10	1036.53098	518.76913	1064.52590	532.76659	Q	822.44536	411.72632	4
11	1123.56301	562.28514	1151.55793	576.28260	S	694.38678	347.69703	3
12	1555.79880	778.40304	1583.79371	792.40049	C-TMT6-	607.35475	304.18101	2
13					R	175.11896	88.06312	1

annotated MSMS spectrum

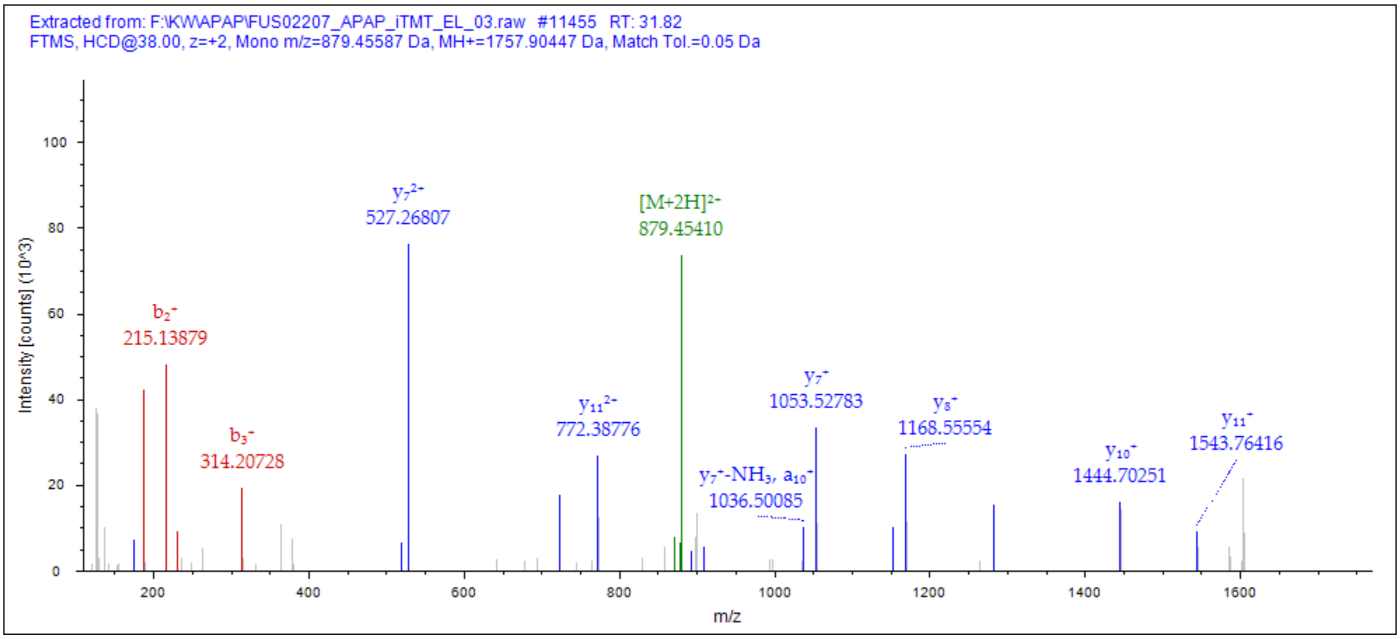

Sequence: LSLLEVGCGTGANFK, C8-TMT6-Cys (329.22660 Da)
Charge: +2, Monoisotopic m/z: 919.50824 Da (+2.39 mmu/+2.6 ppm), MH+: 1838.00920 Da, RT: 58.51 min,
Identified with: Mascot (v1.30); IonScore:40, Exp Value:2.3E-003, Ions matched by search engine: 11/120
Fragment match tolerance used for search: 0.05 Da

b and y ion series

#1	a^+	a^{2+}	b^+	b^{2+}	Seq.	y^+	y^{2+}	#2
1	86.09643	43.55185	114.09135	57.54931	L			15
2	173.12846	87.06787	201.12338	101.06533	S	1724.92035	862.96381	14
3	286.21253	143.60990	314.20745	157.60736	L	1637.88832	819.44780	13
4	399.29660	200.15194	427.29152	214.14940	L	1524.80425	762.90576	12
5	528.33920	264.67324	556.33412	278.67070	E	1411.72018	706.36373	11
6	627.40762	314.20745	655.40254	328.20491	V	1282.67758	641.84243	10
7	684.42909	342.71818	712.42401	356.71564	G	1183.60916	592.30822	9
8	1116.66488	558.83608	1144.65979	572.83353	C-TMT6-	1126.58769	563.79748	8
9	1173.68635	587.34681	1201.68126	601.34427	G	694.35190	347.67959	7
10	1274.73403	637.87065	1302.72894	651.86811	T	637.33043	319.16885	6
11	1331.75550	666.38139	1359.75041	680.37884	G	536.28275	268.64501	5
12	1402.79262	701.89995	1430.78753	715.89740	A	479.26128	240.13428	4
13	1516.83555	758.92141	1544.83046	772.91887	N	408.22416	204.61572	3
14	1663.90397	832.45562	1691.89888	846.45308	F	294.18123	147.59425	2
15					K	147.11281	74.06004	1

annotated MSMS spectrum

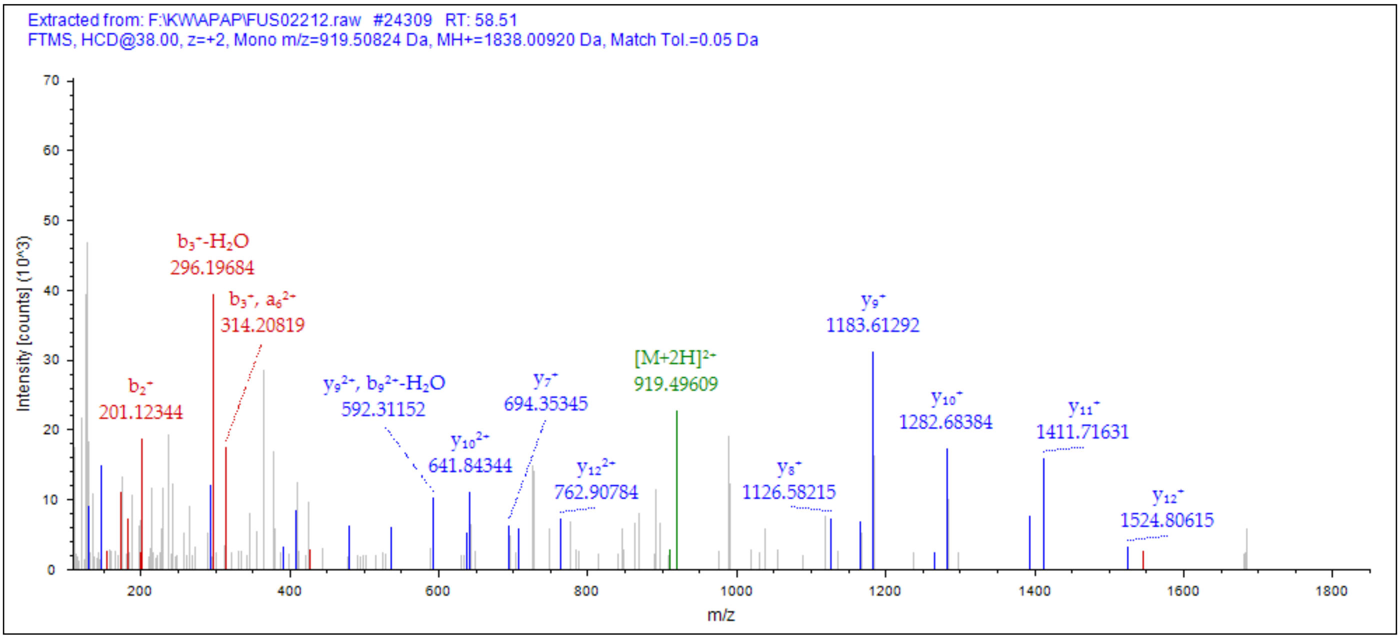

Sequence: SCYWFSR, C2-TMT6-Cys (329.22660 Da)
Charge: +3, Monoisotopic m/z: 426.54782 Da (-0.32 mmu/-0.75 ppm), MH+: 1277.62891 Da, RT: 37.87 min,
Identified with: Mascot (v1.30); IonScore:17, Exp Value:5.4E-001, Ions matched by search engine: 4/48
Fragment match tolerance used for search: 0.05 Da

b and y ion series

#1	a^+	a^{2+}	a^{3+}	b^+	b^{2+}	b^{3+}	Seq.	y^+	y^{2+}	y^{3+}	#2
1	60.04439	30.52583	20.68632	88.03931	44.52329	30.01795	S				7
2	492.28018	246.64373	164.76491	520.27509	260.64118	174.09655	C-TMT6-	1190.59784	595.80256	397.53746	6
3	655.34350	328.17539	219.11935	683.33841	342.17284	228.45099	Y	758.36205	379.68466	253.45887	5
4	841.42282	421.21505	281.14579	869.41773	435.21250	290.47743	W	595.29873	298.15300	199.10443	4
5	988.49124	494.74926	330.16860	1016.48615	508.74671	339.50023	F	409.21941	205.11334	137.07799	3
6	1075.52327	538.26527	359.17927	1103.51818	552.26273	368.51091	S	262.15099	131.57913	88.05518	2
7							R	175.11896	88.06312	59.04450	1

annotated MSMS spectrum

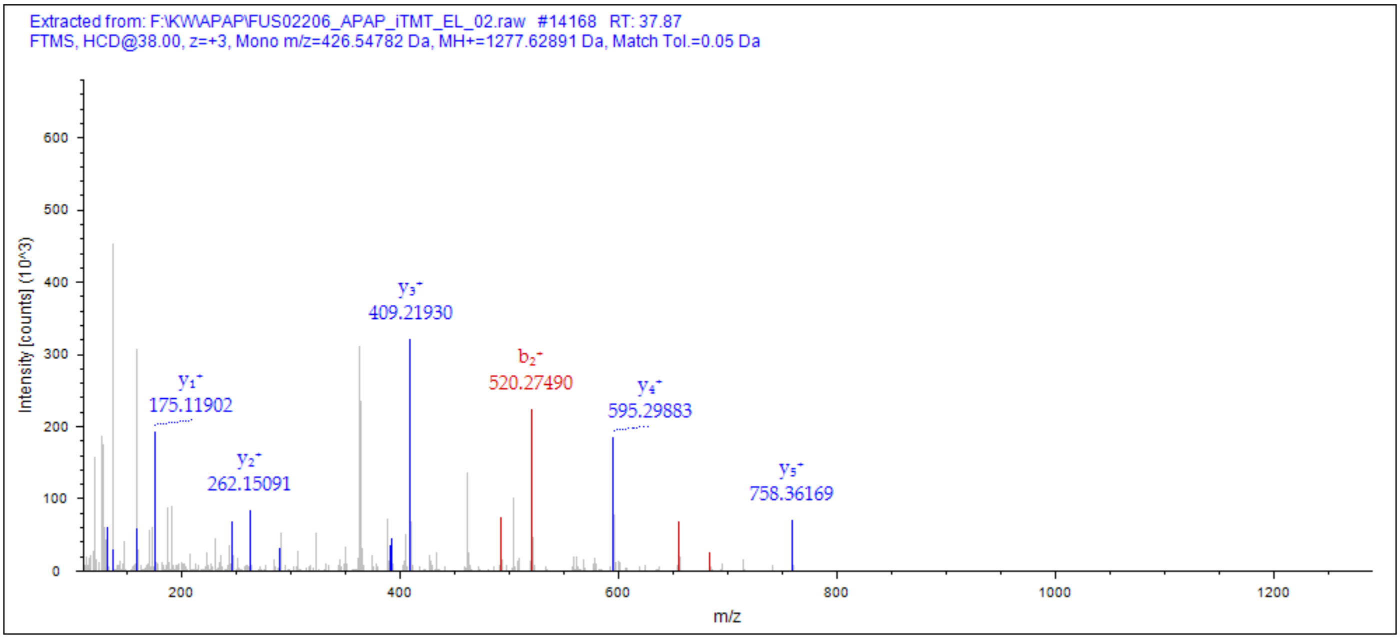

Sequence: TVYFAEEVQCEGNSFHK, C10-TMT6-Cys (329.22660 Da)
Charge: +3, Monoisotopic m/z: 773.04358 Da (+1.36 mmu/+1.76 ppm), MH+: 2317.11618 Da, RT: 43.71 min,
Identified with: Mascot (v1.30); IonScore:23, Exp Value:1.7E-001, Ions matched by search engine: 11/160
Fragment match tolerance used for search: 0.05 Da

b and y ion series

#1	a⁺	a²⁺	a³⁺	b⁺	b²⁺	b³⁺	Seq.	y⁺	y²⁺	y³⁺	#2
1	74.06004	37.53366	25.35820	102.05496	51.53112	34.68984	T				17
2	173.12846	87.06787	58.38101	201.12338	101.06533	67.71264	V	2216.06444	1108.53586	739.35966	16
3	336.19178	168.59953	112.73545	364.18670	182.59699	122.06708	Y	2116.99602	1059.00165	706.33686	15
4	483.26020	242.13374	161.75825	511.25512	256.13120	171.08989	F	1953.93270	977.46999	651.98242	14
5	554.29732	277.65230	185.43729	582.29224	291.64976	194.76893	A	1806.86428	903.93578	602.95961	13
6	683.33992	342.17360	228.45149	711.33484	356.17106	237.78313	E	1735.82716	868.41722	579.28057	12
7	812.38252	406.69490	271.46569	840.37744	420.69236	280.79733	E	1606.78456	803.89592	536.26637	11
8	911.45094	456.22911	304.48850	939.44586	470.22657	313.82014	V	1477.74196	739.37462	493.25217	10
9	1039.50952	520.25840	347.17469	1067.50444	534.25586	356.50633	Q	1378.67354	689.84041	460.22936	9
10	1471.74531	736.37629	491.25329	1499.74022	750.37375	500.58492	C-TMT6-	1250.61496	625.81112	417.54317	8
11	1600.78791	800.89759	534.26749	1628.78282	814.89505	543.59912	E	818.37917	409.69322	273.46457	7
12	1657.80938	829.40833	553.27464	1685.80429	843.40578	562.60628	G	689.33657	345.17192	230.45037	6
13	1771.85231	886.42979	591.28895	1799.84722	900.42725	600.62059	N	632.31510	316.66119	211.44322	5
14	1858.88434	929.94581	620.29963	1886.87925	943.94326	629.63127	S	518.27217	259.63972	173.42891	4
15	2005.95276	1003.48002	669.32244	2033.94767	1017.47747	678.65407	F	431.24014	216.12371	144.41823	3
16	2143.01167	1072.00947	715.00874	2171.00658	1086.00693	724.34038	H	284.17172	142.58950	95.39542	2
17							K	147.11281	74.06004	49.70912	1

annotated MSMS spectrum

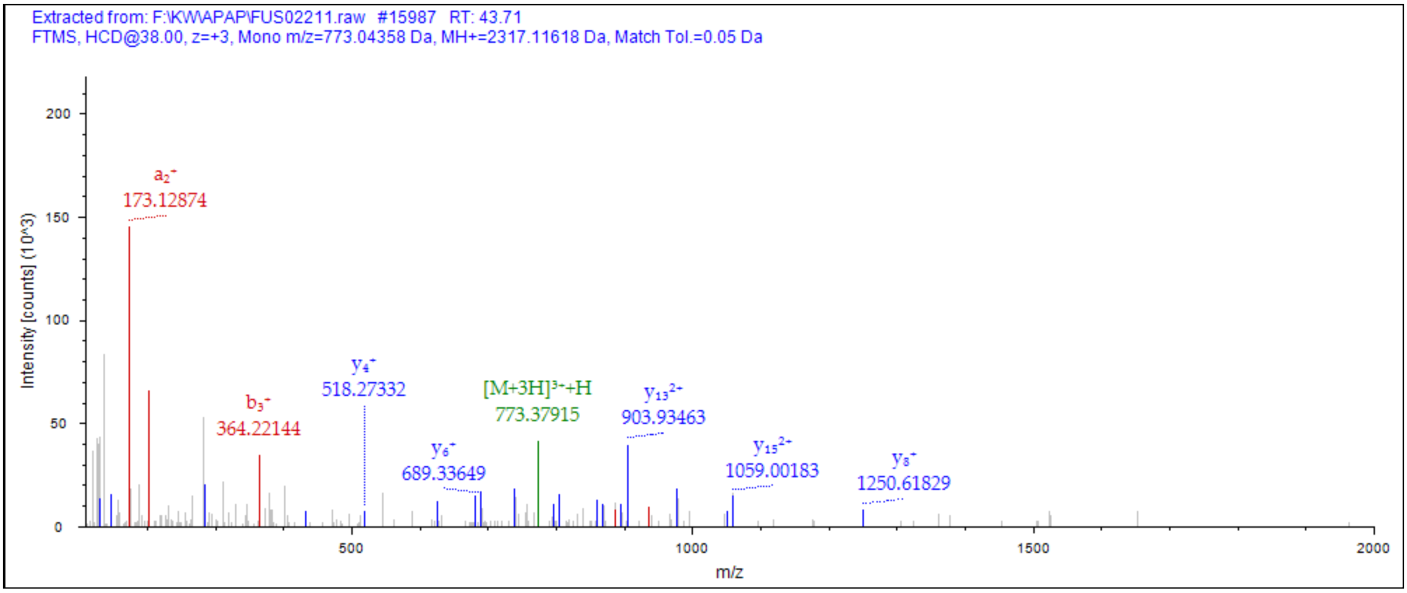

Sequence: ESCLEAYTGIVQGLK, C3-TMT6-Cys (329.22660 Da)
Charge: +2, Monoisotopic m/z: 970.52417 Da (+2.48 mmu/+2.56 ppm), MH+: 1940.04106 Da, RT: 64.87 min,
Identified with: Mascot (v1.30); IonScore:35, Exp Value:8.9E-003, Ions matched by search engine: 7/124
Fragment match tolerance used for search: 0.05 Da

b and y ion series

#1	a⁺	a²⁺	b⁺	b²⁺	Seq.	y⁺	y²⁺	#2
1	102.05496	51.53112	130.04988	65.52858	E			15
2	189.08699	95.04713	217.08191	109.04459	S	1810.99350	906.00039	14
3	621.32278	311.16503	649.31769	325.16248	C-TMT6-	1723.96147	862.48437	13
4	734.40685	367.70706	762.40176	381.70452	L	1291.72568	646.36648	12
5	863.44945	432.22836	891.44436	446.22582	E	1178.64161	589.82444	11
6	934.48657	467.74692	962.48148	481.74438	A	1049.59901	525.30314	10
7	1097.54989	549.27858	1125.54480	563.27604	Y	978.56189	489.78458	9
8	1198.59757	599.80242	1226.59248	613.79988	T	815.49857	408.25292	8
9	1255.61904	628.31316	1283.61395	642.31061	G	714.45089	357.72908	7
10	1368.70311	684.85519	1396.69802	698.85265	I	657.42942	329.21835	6
11	1467.77153	734.38940	1495.76644	748.38686	V	544.34535	272.67631	5
12	1595.83011	798.41869	1623.82502	812.41615	Q	445.27693	223.14210	4
13	1652.85158	826.92943	1680.84649	840.92688	G	317.21835	159.11281	3
14	1765.93565	883.47146	1793.93056	897.46892	L	260.19688	130.60208	2
15					K	147.11281	74.06004	1

annotated MSMS spectrum

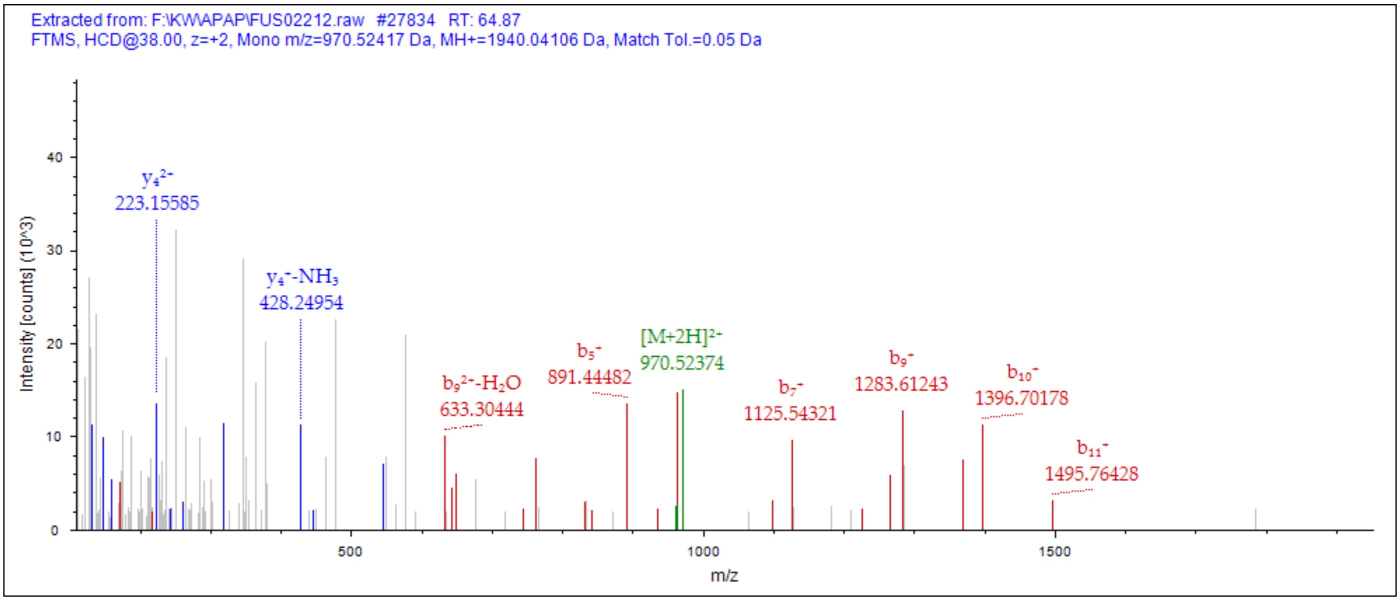

Sequence: TSYGWIEIVGCADR, C11-TMT6-Cys (329.22660 Da)
Charge: +2, Monoisotopic m/z: 949.98529 Da (+0.02 mmu/+0.02 ppm), MH+: 1898.96330 Da, RT: 66.15 min,
Identified with: Mascot (v1.30); IonScore:39, Exp Value:4.7E-003, Ions matched by search engine: 7/104
Fragment match tolerance used for search: 0.05 Da

b and y ion series

#1	a^+	a^{2+}	b^+	b^{2+}	Seq.	y^+	y^{2+}	#2
1	74.06004	37.53366	102.05496	51.53112	T			14
2	161.09207	81.04967	189.08699	95.04713	S	1797.91559	899.46143	13
3	324.15539	162.58133	352.15031	176.57879	Y	1710.88356	855.94542	12
4	381.17686	191.09207	409.17178	205.08953	G	1547.82024	774.41376	11
5	567.25618	284.13173	595.25110	298.12919	W	1490.79877	745.90302	10
6	680.34025	340.67376	708.33517	354.67122	I	1304.71945	652.86336	9
7	809.38285	405.19506	837.37777	419.19252	E	1191.63538	596.32133	8
8	922.46692	461.73710	950.46184	475.73456	I	1062.59278	531.80003	7
9	1021.53534	511.27131	1049.53026	525.26877	V	949.50871	475.25799	6
10	1078.55681	539.78204	1106.55173	553.77950	G	850.44029	425.72378	5
11	1510.79260	755.89994	1538.78751	769.89739	C-TMT6-	793.41882	397.21305	4
12	1581.82972	791.41850	1609.82463	805.41595	A	361.18303	181.09515	3
13	1696.85667	848.93197	1724.85158	862.92943	D	290.14591	145.57659	2
14					R	175.11896	88.06312	1

annotated MSMS spectrum

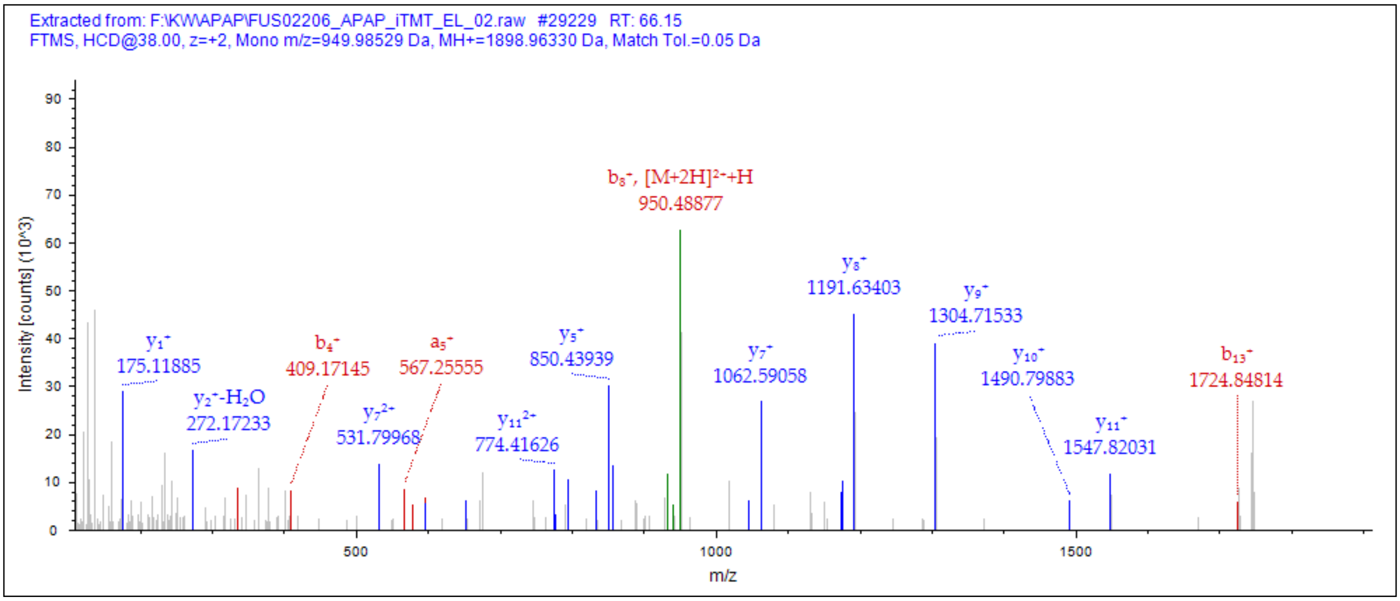

Sequence: GYVSCALGCPYEGK, C5-TMT6-Cys (329.22660 Da), C9-TMT6-Cys (329.22660 Da)
Charge: +3, Monoisotopic m/z: 702.36914 Da (+0.14 mmu/+0.2 ppm), MH+: 2105.09287 Da, RT: 32.04 min,
Identified with: Mascot (v1.30); IonScore:16, Exp Value:1.0E+000, Ions matched by search engine: 9/104
Fragment match tolerance used for search: 0.05 Da

b and y ion series

#1	a^{+}	a^{2+}	a^{3+}	b^{+}	b^{2+}	b^{3+}	Seq.	y^{+}	y^{2+}	y^{3+}	#2
1	30.03383	15.52055	10.68280	58.02875	29.51801	20.01443	G				14
2	193.09715	97.05221	65.03724	221.09207	111.04967	74.36887	Y	2048.07097	1024.53912	683.36184	13
3	292.16557	146.58642	98.06004	320.16049	160.58388	107.39168	V	1885.00765	943.00746	629.00740	12
4	379.19760	190.10244	127.07072	407.19252	204.09990	136.40236	S	1785.93923	893.47325	595.98459	11
5	811.43339	406.22033	271.14931	839.42830	420.21779	280.48095	C-TMT6-	1698.90720	849.95724	566.97392	10
6	882.47051	441.73889	294.82835	910.46542	455.73635	304.15999	A	1266.67142	633.83935	422.89532	9
7	995.55458	498.28093	332.52304	1023.54949	512.27838	341.85468	L	1195.63430	598.32079	399.21628	8
8	1052.57605	526.79166	351.53020	1080.57096	540.78912	360.86184	G	1082.55023	541.77875	361.52159	7
9	1484.81183	742.90955	495.60880	1512.80675	756.90701	504.94043	C-TMT6-	1025.52876	513.26802	342.51444	6
10	1581.86460	791.43594	527.95972	1609.85952	805.43340	537.29136	P	593.29297	297.15012	198.43584	5
11	1744.92792	872.96760	582.31416	1772.92284	886.96506	591.64580	Y	496.24020	248.62374	166.08492	4
12	1873.97052	937.48890	625.32836	1901.96544	951.48636	634.66000	E	333.17688	167.09208	111.73048	3
13	1930.99199	965.99963	644.33552	1958.98691	979.99709	653.66715	G	204.13428	102.57078	68.71628	2
14							K	147.11281	74.06004	49.70912	1

annotated MSMS spectrum

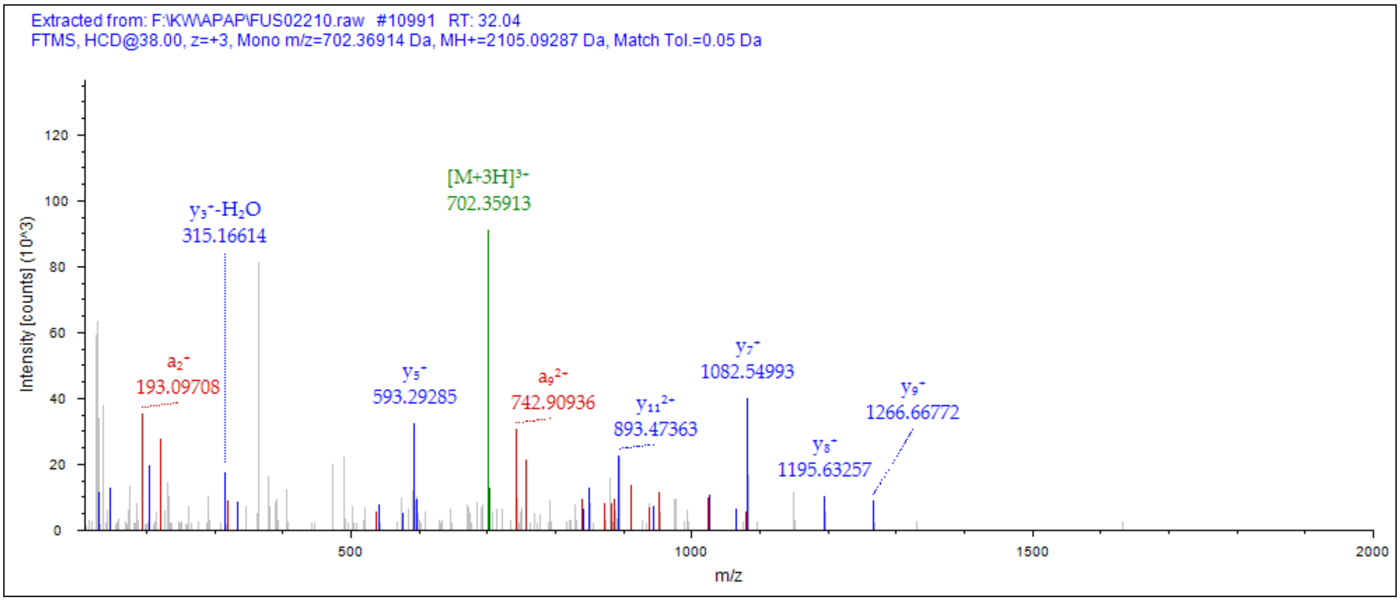

Sequence: GCEVVVSGK, C2-TMT6-Cys (329.22660 Da)
Charge: +2, Monoisotopic m/z: 603.83942 Da (+0.07 mmu/+0.11 ppm), MH+: 1206.67156 Da, RT: 16.20 min,
Identified with: Mascot (v1.30); IonScore:26, Exp Value:6.1E-002, Ions matched by search engine: 5/64
Fragment match tolerance used for search: 0.05 Da

b and y ion series

#1	a⁺	a²⁺	b⁺	b²⁺	Seq.	y⁺	y²⁺	#2
1	30.03383	15.52055	58.02875	29.51801	G			9
2	462.26962	231.63845	490.26453	245.63590	C-TMT6-	1149.64996	575.32862	8
3	591.31222	296.15975	619.30713	310.15720	E	717.41417	359.21072	7
4	690.38064	345.69396	718.37555	359.69141	V	588.37157	294.68942	6
5	789.44906	395.22817	817.44397	409.22562	V	489.30315	245.15521	5
6	888.51748	444.76238	916.51239	458.75983	V	390.23473	195.62100	4
7	975.54951	488.27839	1003.54442	502.27585	S	291.16631	146.08679	3
8	1032.57098	516.78913	1060.56589	530.78658	G	204.13428	102.57078	2
9					K	147.11281	74.06004	1

annotated MSMS spectrum

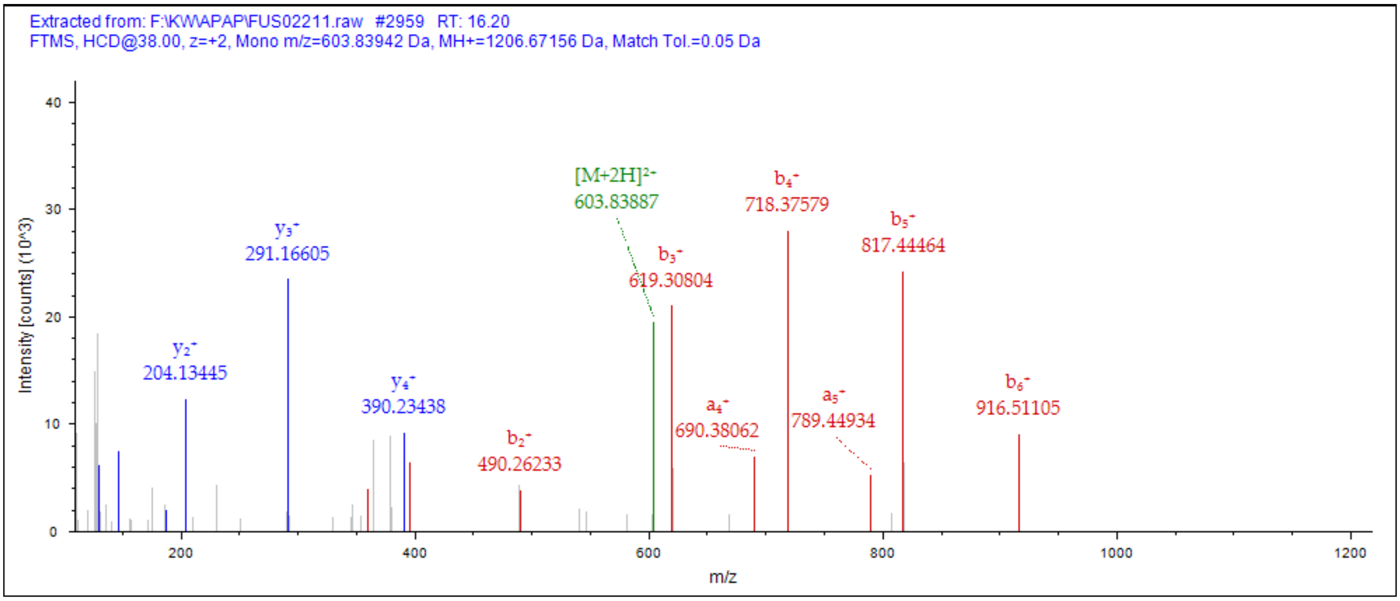

Sequence: HPHDIIDDINSGAVECPAS, C16-TMT6-Cys (329.22660 Da)
Charge: +2, Monoisotopic m/z: 1160.06506 Da (-0.46 mmu/-0.39 ppm), MH+: 2319.12285 Da, RT: 47.78 min,
Identified with: Mascot (v1.30); IonScore:58, Exp Value:5.0E-005, Ions matched by search engine: 9/162
Fragment match tolerance used for search: 0.05 Da

b and y ion series

#1	a^+	a^{2+}	b^+	b^{2+}	Seq.	y^+	y^{2+}	#2
1	110.07127	55.53927	138.06619	69.53673	H			19
2	207.12404	104.06566	235.11896	118.06312	P	2182.06486	1091.53607	18
3	344.18295	172.59511	372.17787	186.59257	H	2085.01209	1043.00968	17
4	459.20990	230.10859	487.20482	244.10605	D	1947.95318	974.48023	16
5	572.29397	286.65062	600.28889	300.64808	I	1832.92623	916.96675	15
6	685.37804	343.19266	713.37296	357.19012	I	1719.84216	860.42472	14
7	800.40499	400.70613	828.39991	414.70359	D	1606.75809	803.88268	13
8	915.43194	458.21961	943.42686	472.21707	D	1491.73114	746.36921	12
9	1028.51601	514.76164	1056.51093	528.75910	I	1376.70419	688.85573	11
10	1142.55894	571.78311	1170.55386	585.78057	N	1263.62012	632.31370	10
11	1229.59097	615.29912	1257.58589	629.29658	S	1149.57719	575.29223	9
12	1286.61244	643.80986	1314.60736	657.80732	G	1062.54516	531.77622	8
13	1357.64956	679.32842	1385.64448	693.32588	A	1005.52369	503.26548	7
14	1456.71798	728.86263	1484.71290	742.86009	V	934.48657	467.74692	6
15	1585.76058	793.38393	1613.75550	807.38139	E	835.41815	418.21271	5
16	2017.99637	1009.50182	2045.99128	1023.49928	C-TMT6-	706.37555	353.69141	4
17	2115.04914	1058.02821	2143.04405	1072.02566	P	274.13976	137.57352	3
18	2186.08626	1093.54677	2214.08117	1107.54422	A	177.08699	89.04713	2
19					S	106.04987	53.52857	1

annotated MSMS spectrum

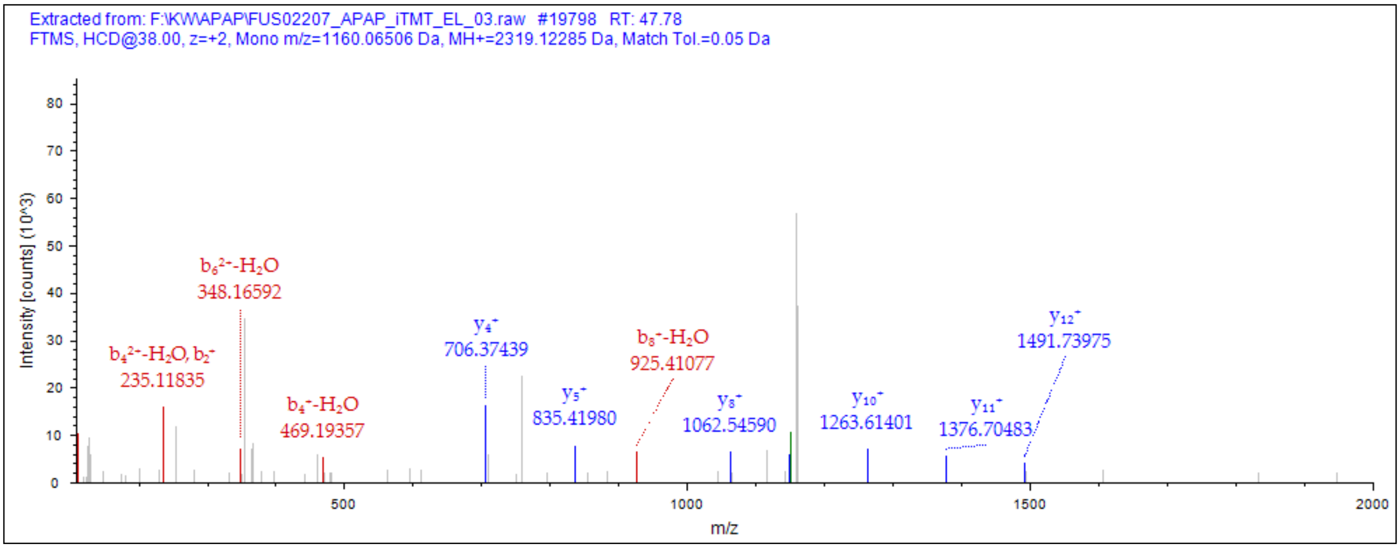

Sequence: CSWLVVQCLQR, C1-TMT6-Cys (329.22660 Da), C8-TMT6-Cys (329.22660 Da)
Charge: +4, Monoisotopic m/z: 499.03543 Da (-1.03 mmu/-2.07 ppm), MH+: 1993.11989 Da, RT: 47.31 min,
Identified with: Mascot (v1.30); IonScore:13, Exp Value:6.3E-001, Ions matched by search engine: 5/96
Fragment match tolerance used for search: 0.05 Da

b and y ion series

#1	a^+	a^{2+}	a^{3+}	a^{4+}	b^+	b^{2+}	b^{3+}	b^{4+}	Seq.	y^+	y^{2+}	y^{3+}	y^{4+}	#2
1	405.24815	203.12771	135.75423	102.06749	433.24306	217.12517	145.08587	109.06622	C-TMT6-					11
2	492.28018	246.64373	164.76491	123.82550	520.27509	260.64118	174.09655	130.82423	S	1560.88824	780.94776	520.96760	390.97752	10
3	678.35950	339.68339	226.79135	170.34533	706.35441	353.68084	236.12299	177.34406	W	1473.85621	737.43174	491.95692	369.21951	9
4	791.44357	396.22542	264.48604	198.61635	819.43848	410.22288	273.81768	205.61508	L	1287.77689	644.39208	429.93048	322.69968	8
5	890.51199	445.75963	297.50885	223.38345	918.50690	459.75709	306.84048	230.38218	V	1174.69282	587.85005	392.23579	294.42866	7
6	989.58041	495.29384	330.53165	248.15056	1017.57532	509.29130	339.86329	255.14929	V	1075.62440	538.31584	359.21298	269.66156	6
7	1117.63899	559.32313	373.21785	280.16520	1145.63390	573.32059	382.54948	287.16393	Q	976.55598	488.78163	326.19018	244.89445	5
8	1549.87477	775.44102	517.29644	388.22415	1577.86969	789.43848	526.62808	395.22288	C-TMT6-	848.49740	424.75234	283.50398	212.87981	4
9	1662.95884	831.98306	554.99113	416.49517	1690.95376	845.98052	564.32277	423.49390	L	416.26161	208.63444	139.42539	104.82086	3
10	1791.01742	896.01235	597.67733	448.50981	1819.01234	910.00981	607.00896	455.50854	Q	303.17754	152.09241	101.73070	76.54984	2
11									R	175.11896	88.06312	59.04450	44.53520	1

annotated MSMS spectrum

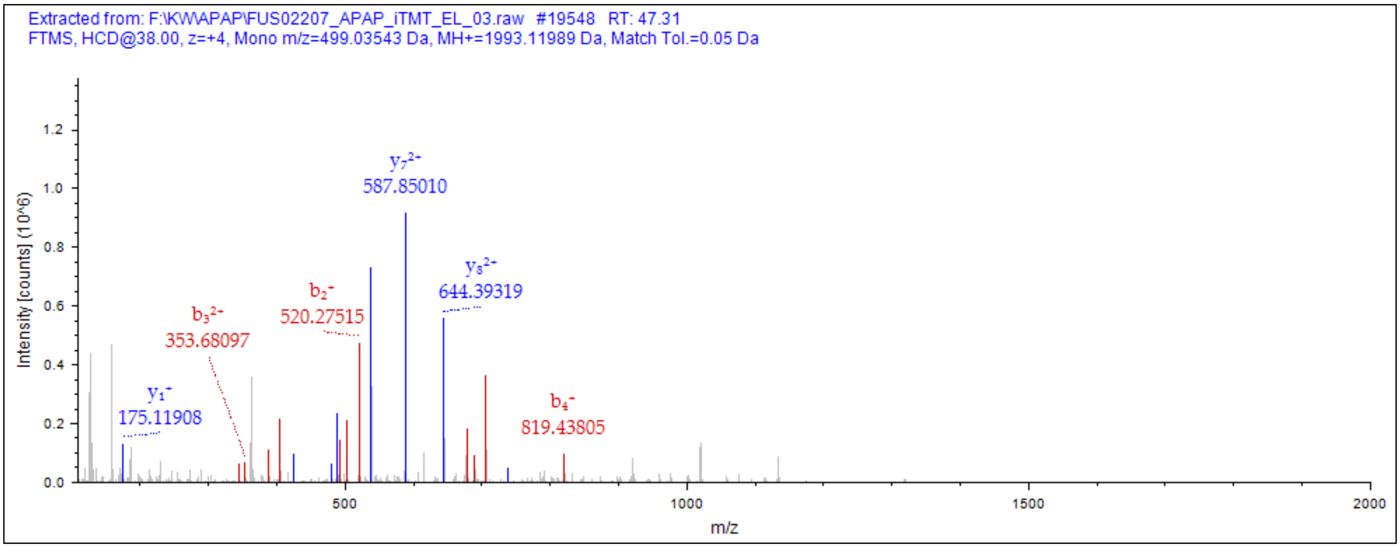

Sequence: GCITIIGGGDTATCCAK, C2-TMT6-Cys (329.22660 Da), C14-TMT6-Cys (329.22660 Da), C15-TMT6-Cys (329.22660 Da)
Charge: +3, Monoisotopic m/z: 857.80432 Da (-1.36 mmu/-1.59 ppm), MH+: 2571.39841 Da, RT: 29.14 min,
Identified with: Mascot (v1.30); IonScore:14, Exp Value:6.8E-001, Ions matched by search engine: 13/128
Fragment match tolerance used for search: 0.05 Da

b and y ion series

#1	a^{+}	a^{2+}	a^{3+}	b^{+}	b^{2+}	b^{3+}	Seq.	y^{+}	y^{2+}	y^{3+}	#2
1	30.03383	15.52055	10.68280	58.02875	29.51801	20.01443	G				17
2	462.26962	231.63845	154.76139	490.26453	245.63590	164.09303	C-TMT6-	2514.38102	1257.69415	838.79852	16
3	575.35369	288.18048	192.45608	603.34860	302.17794	201.78772	I	2082.14523	1041.57625	694.71993	15
4	676.40137	338.70432	226.13864	704.39628	352.70178	235.47028	T	1969.06116	985.03422	657.02524	14
5	789.48544	395.24636	263.83333	817.48035	409.24381	273.16497	I	1868.01348	934.51038	623.34268	13
6	902.56951	451.78839	301.52802	930.56442	465.78585	310.85966	I	1754.92941	877.96834	585.64799	12
7	959.59098	480.29913	320.53518	987.58589	494.29658	329.86681	G	1641.84534	821.42631	547.95330	11
8	1016.61245	508.80986	339.54233	1044.60736	522.80732	348.87397	G	1584.82387	792.91557	528.94614	10
9	1073.63392	537.32060	358.54949	1101.62883	551.31805	367.88113	G	1527.80240	764.40484	509.93898	9
10	1188.66087	594.83407	396.89181	1216.65578	608.83153	406.22344	D	1470.78093	735.89410	490.93183	8
11	1289.70855	645.35791	430.57437	1317.70346	659.35537	439.90600	T	1355.75398	678.38063	452.58951	7
12	1360.74567	680.87647	454.25341	1388.74058	694.87393	463.58504	A	1254.70630	627.85679	418.90695	6
13	1461.79335	731.40031	487.93597	1489.78826	745.39777	497.26760	T	1183.66918	592.33823	395.22791	5
14	1894.02913	947.51820	632.01456	1922.02405	961.51566	641.34620	C-TMT6-	1082.62150	541.81439	361.54535	4
15	2326.26492	1163.63610	776.09316	2354.25983	1177.63355	785.42479	C-TMT6-	650.38572	325.69650	217.46676	3
16	2397.30204	1199.15466	799.77220	2425.29695	1213.15211	809.10383	A	218.14993	109.57860	73.38816	2
17							K	147.11281	74.06004	49.70912	1

annotated MSMS spectrum

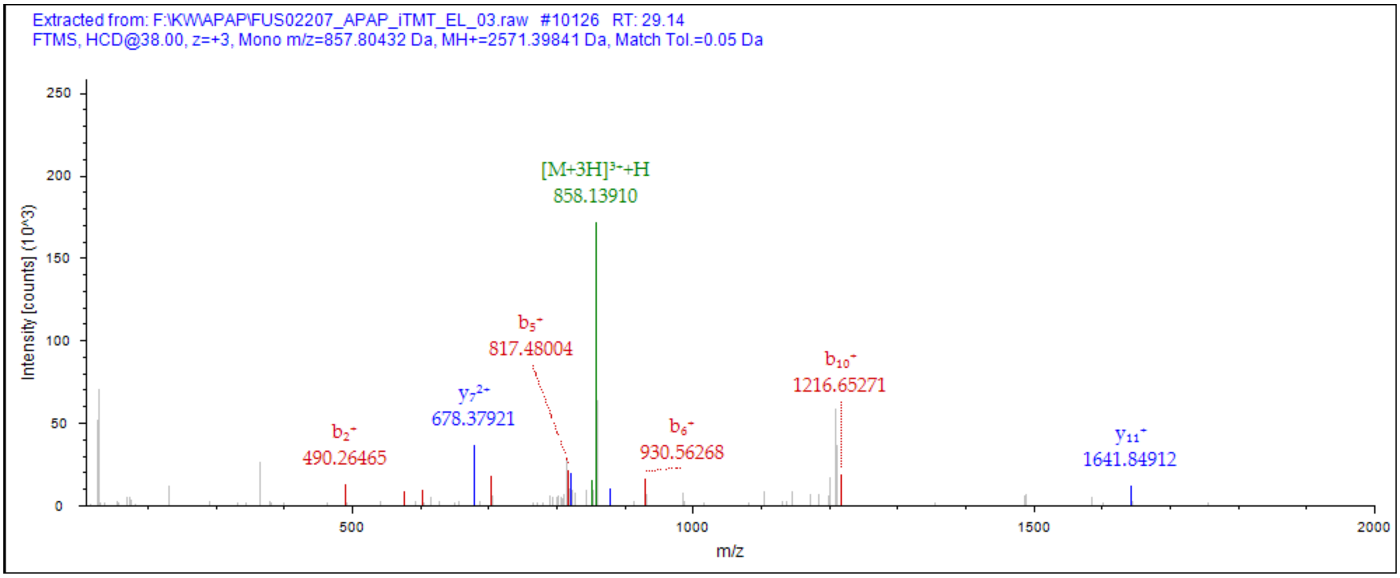

Sequence: TLCGTPTYLAPEVLVSVGTAGYNR, C3-TMT6-Cys (329.22660 Da)
Charge: +3, Monoisotopic m/z: 937.83649 Da (+1.19 mmu/+1.27 ppm), MH+: 2811.49491 Da, RT: 76.50 min,
Identified with: Mascot (v1.30); IonScore:50, Exp Value:3.0E-004, Ions matched by search engine: 8/188
Fragment match tolerance used for search: 0.05 Da

b and y ion series

#1	a⁺	a²⁺	a³⁺	b⁺	b²⁺	b³⁺	Seq.	y⁺	y²⁺	y³⁺	#2
1	74.06004	37.53366	25.35820	102.05496	51.53112	34.68984	T				24
2	187.14411	94.07569	63.05289	215.13903	108.07315	72.38453	L	2710.44365	1355.72546	904.15273	23
3	619.37990	310.19359	207.13148	647.37481	324.19104	216.46312	C-TMT6-	2597.35958	1299.18343	866.45804	22
4	676.40137	338.70432	226.13864	704.39628	352.70178	235.47028	G	2165.12379	1083.06553	722.37945	21
5	777.44905	389.22816	259.82120	805.44396	403.22562	269.15284	T	2108.10232	1054.55480	703.37229	20
6	874.50182	437.75455	292.17212	902.49673	451.75200	301.50376	P	2007.05464	1004.03096	669.68973	19
7	975.54950	488.27839	325.85468	1003.54441	502.27584	335.18632	T	1910.00187	955.50457	637.33881	18
8	1138.61282	569.81005	380.20912	1166.60773	583.80750	389.54076	Y	1808.95419	904.98073	603.65625	17
9	1251.69689	626.35208	417.90381	1279.69180	640.34954	427.23545	L	1645.89087	823.44907	549.30181	16
10	1322.73401	661.87064	441.58285	1350.72892	675.86810	450.91449	A	1532.80680	766.90704	511.60712	15
11	1419.78678	710.39703	473.93378	1447.78169	724.39448	483.26541	P	1461.76968	731.38848	487.92808	14
12	1548.82938	774.91833	516.94798	1576.82429	788.91578	526.27961	E	1364.71691	682.86209	455.57715	13
13	1647.89780	824.45254	549.97078	1675.89271	838.44999	559.30242	V	1235.67431	618.34079	412.56295	12
14	1760.98187	880.99457	587.66547	1788.97678	894.99203	596.99711	L	1136.60589	568.80658	379.54015	11
15	1860.05029	930.52878	620.68828	1888.04520	944.52624	630.01992	V	1023.52182	512.26455	341.84546	10
16	1947.08232	974.04480	649.69896	1975.07723	988.04225	659.03059	S	924.45340	462.73034	308.82265	9
17	2046.15074	1023.57901	682.72176	2074.14565	1037.57646	692.05340	V	837.42137	419.21432	279.81197	8
18	2103.17221	1052.08974	701.72892	2131.16712	1066.08720	711.06056	G	738.35295	369.68011	246.78917	7
19	2204.21989	1102.61358	735.41148	2232.21480	1116.61104	744.74312	T	681.33148	341.16938	227.78201	6
20	2275.25701	1138.13214	759.09052	2303.25192	1152.12960	768.42216	A	580.28380	290.64554	194.09945	5
21	2332.27848	1166.64288	778.09768	2360.27339	1180.64033	787.42931	G	509.24668	255.12698	170.42041	4
22	2495.34180	1248.17454	832.45212	2523.33671	1262.17199	841.78375	Y	452.22521	226.61624	151.41325	3
23	2609.38473	1305.19600	870.46643	2637.37964	1319.19346	879.79806	N	289.16189	145.08458	97.05881	2
24							R	175.11896	88.06312	59.04450	1

annotated MSMS spectrum

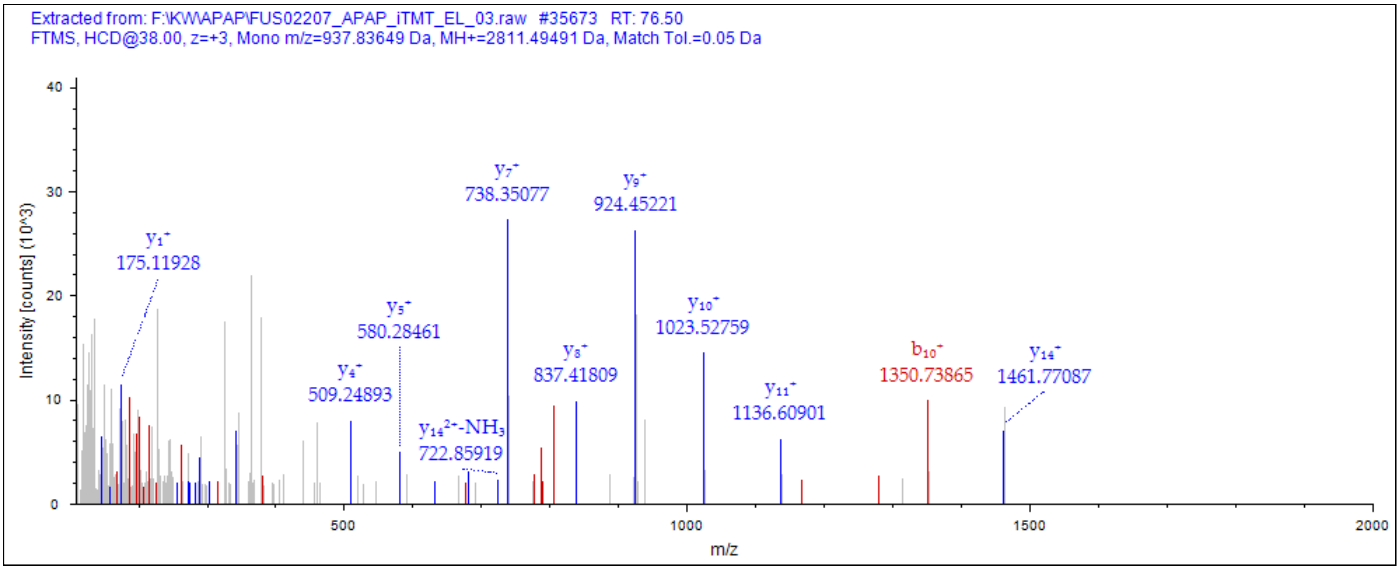

Sequence: CFSVLGFCK, C1-TMT6-Cys (329.22660 Da), C8-TMT6-Cys (329.22660 Da)
Charge: +4, Monoisotopic m/z: 416.23740 Da (+0.14 mmu/+0.33 ppm), MH+: 1661.92776 Da, RT: 49.55 min,
Identified with: Mascot (v1.30); IonScore:12, Exp Value:1.4E+000, Ions matched by search engine: 6/64
Fragment match tolerance used for search: 0.05 Da

b and y ion series

#1	a^+	a^{2+}	a^{3+}	a^{4+}	b^+	b^{2+}	b^{3+}	b^{4+}	Seq.	y^+	y^{2+}	y^{3+}	y^{4+}	#2
1	405.24815	203.12771	135.75423	102.06749	433.24306	217.12517	145.08587	109.06622	C-TMT6-					9
2	552.31657	276.66192	184.77704	138.83460	580.31148	290.65938	194.10868	145.83333	F	1229.69143	615.34935	410.56866	308.17831	8
3	639.34860	320.17794	213.78772	160.59261	667.34351	334.17539	223.11935	167.59134	S	1082.62301	541.81514	361.54585	271.41121	7
4	738.41702	369.71215	246.81052	185.35971	766.41193	383.70960	256.14216	192.35844	V	995.59098	498.29913	332.53518	249.65320	6
5	851.50109	426.25418	284.50521	213.63073	879.49600	440.25164	293.83685	220.62946	L	896.52256	448.76492	299.51237	224.88610	5
6	908.52256	454.76492	303.51237	227.88610	936.51747	468.76237	312.84401	234.88483	G	783.43849	392.22288	261.81768	196.61508	4
7	1055.59098	528.29913	352.53518	264.65320	1083.58589	542.29658	361.86681	271.65193	F	726.41702	363.71215	242.81052	182.35971	3
8	1487.82676	744.41702	496.61377	372.71215	1515.82168	758.41448	505.94541	379.71088	C-TMT6-	579.34860	290.17794	193.78772	145.59261	2
9									K	147.11281	74.06004	49.70912	37.53366	1

annotated MSMS spectrum

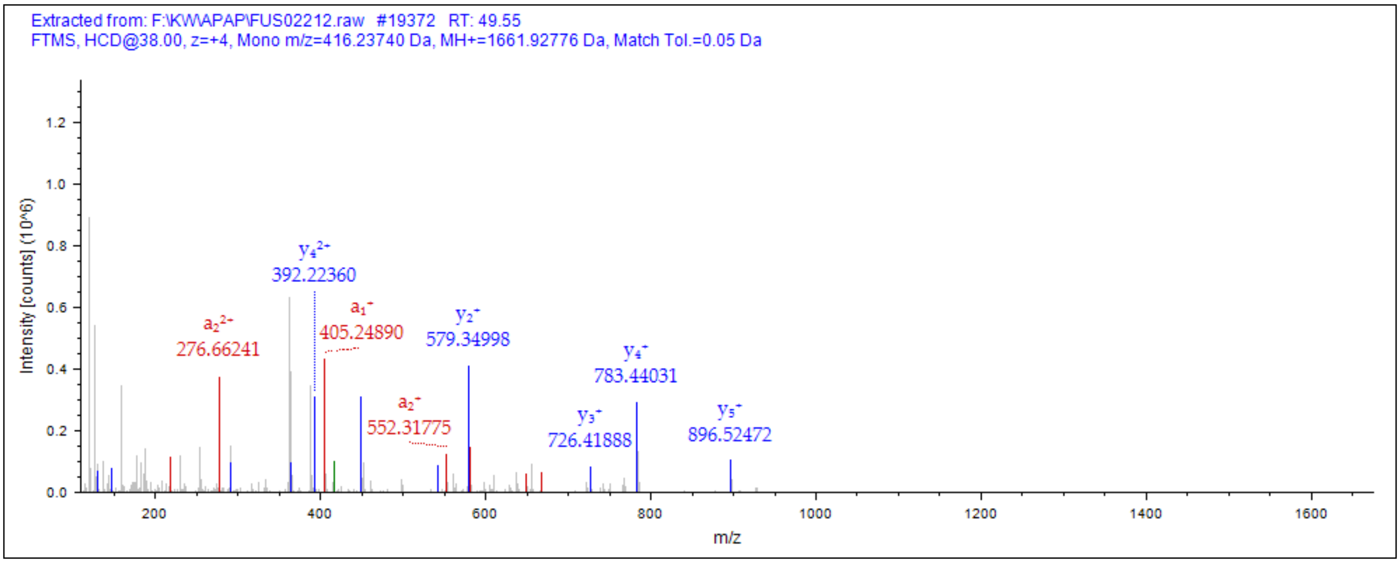

Sequence: HLCQQLQAEQAAAEK, C3-TMT6-Cys (329.22660 Da)
Charge: +3, Monoisotopic m/z: 666.35266 Da (-0.07 mmu/-0.11 ppm), MH+: 1997.04343 Da, RT: 19.89 min,
Identified with: Mascot (v1.30); IonScore:53, Exp Value:1.9E-004, Ions matched by search engine: 9/156
Fragment match tolerance used for search: 0.05 Da

b and y ion series

#1	a⁺	a²⁺	a³⁺	b⁺	b²⁺	b³⁺	Seq.	y⁺	y²⁺	y³⁺	#2
1	110.07127	55.53927	37.36194	138.06619	69.53673	46.69358	H				15
2	223.15534	112.08131	75.05663	251.15026	126.07877	84.38827	L	1859.98474	930.49601	620.66643	14
3	655.39113	328.19920	219.13523	683.38604	342.19666	228.46686	C-TMT6-	1746.90067	873.95397	582.97174	13
4	783.44971	392.22849	261.82142	811.44462	406.22595	271.15306	Q	1314.66488	657.83608	438.89314	12
5	911.50829	456.25778	304.50761	939.50320	470.25524	313.83925	Q	1186.60630	593.80679	396.20695	11
6	1024.59236	512.79982	342.20230	1052.58727	526.79727	351.53394	L	1058.54772	529.77750	353.52076	10
7	1152.65094	576.82911	384.88850	1180.64585	590.82656	394.22013	Q	945.46365	473.23546	315.82607	9
8	1223.68806	612.34767	408.56754	1251.68297	626.34512	417.89917	A	817.40507	409.20617	273.13987	8
9	1352.73066	676.86897	451.58174	1380.72557	690.86642	460.91337	E	746.36795	373.68761	249.46083	7
10	1480.78924	740.89826	494.26793	1508.78415	754.89571	503.59957	Q	617.32535	309.16631	206.44663	6
11	1551.82636	776.41682	517.94697	1579.82127	790.41427	527.27861	A	489.26677	245.13702	163.76044	5
12	1622.86348	811.93538	541.62601	1650.85839	825.93283	550.95765	A	418.22965	209.61846	140.08140	4
13	1693.90060	847.45394	565.30505	1721.89551	861.45139	574.63669	A	347.19253	174.09990	116.40236	3
14	1822.94320	911.97524	608.31925	1850.93811	925.97269	617.65089	E	276.15541	138.58134	92.72332	2
15							K	147.11281	74.06004	49.70912	1

annotated MSMS spectrum

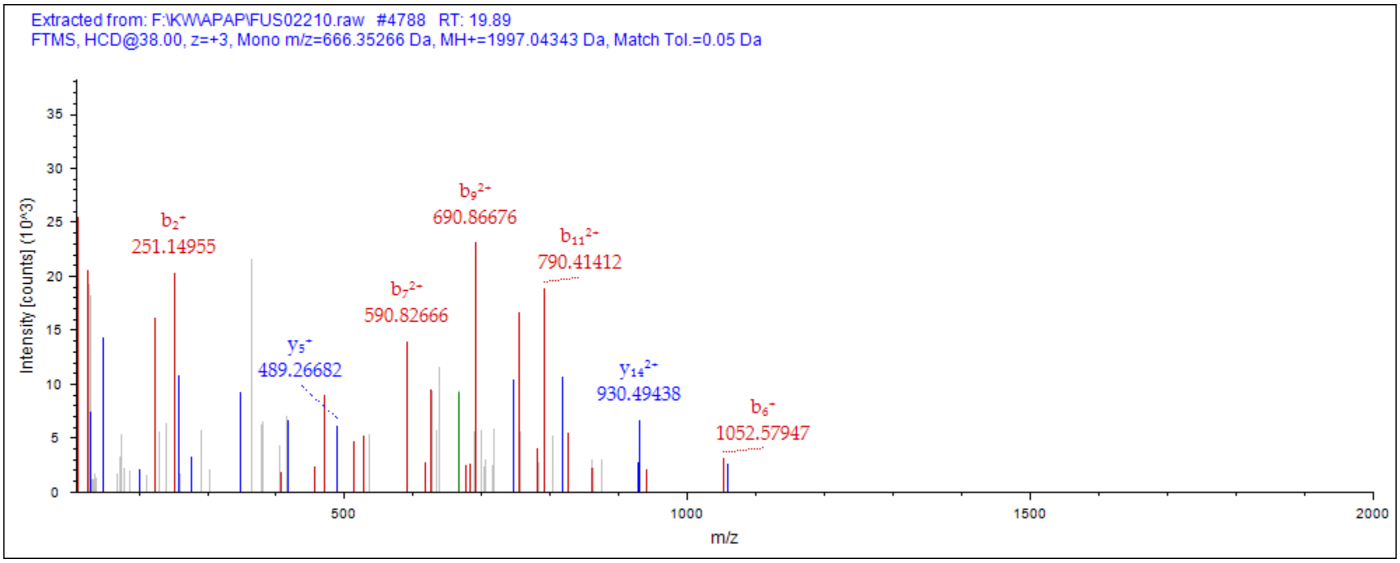

Sequence: NIELICQENEGENDPVLQR, C6-TMT6-Cys (329.22660 Da)
Charge: +3, Monoisotopic m/z: 848.09656 Da (-0.62 mmu/-0.73 ppm), MH+: 2542.27512 Da, RT: 50.92 min,
Identified with: Mascot (v1.30); IonScore:29, Exp Value:4.3E-002, Ions matched by search engine: 12/216
Fragment match tolerance used for search: 0.05 Da

b and y ion series

#1	a^{+}	a^{2+}	a^{3+}	b^{+}	b^{2+}	b^{3+}	Seq.	y^{+}	y^{2+}	y^{3+}	#2
1	87.05529	44.03128	29.68995	115.05021	58.02874	39.02159	N				19
2	200.13936	100.57332	67.38464	228.13428	114.57078	76.71628	I	2428.23406	1214.62067	810.08287	18
3	329.18196	165.09462	110.39884	357.17688	179.09208	119.73048	E	2315.14999	1158.07863	772.38818	17
4	442.26603	221.63665	148.09353	470.26095	235.63411	157.42517	L	2186.10739	1093.55733	729.37398	16
5	555.35010	278.17869	185.78822	583.34502	292.17615	195.11986	I	2073.02332	1037.01530	691.67929	15
6	987.58589	494.29658	329.86681	1015.58080	508.29404	339.19845	C-TMT6-	1959.93925	980.47326	653.98460	14
7	1115.64447	558.32587	372.55301	1143.63938	572.32333	381.88464	Q	1527.70346	764.35537	509.90600	13
8	1244.68707	622.84717	415.56721	1272.68198	636.84463	424.89884	E	1399.64488	700.32608	467.21981	12
9	1358.73000	679.86864	453.58152	1386.72491	693.86609	462.91315	N	1270.60228	635.80478	424.20561	11
10	1487.77260	744.38994	496.59572	1515.76751	758.38739	505.92735	E	1156.55935	578.78331	386.19130	10
11	1544.79407	772.90067	515.60287	1572.78898	786.89813	524.93451	G	1027.51675	514.26201	343.17710	9
12	1673.83667	837.42197	558.61707	1701.83158	851.41943	567.94871	E	970.49528	485.75128	324.16994	8
13	1787.87960	894.44344	596.63138	1815.87451	908.44089	605.96302	N	841.45268	421.22998	281.15574	7
14	1902.90655	951.95691	634.97370	1930.90146	965.95437	644.30534	D	727.40975	364.20851	243.14143	6
15	1999.95932	1000.48330	667.32462	2027.95423	1014.48075	676.65626	P	612.38280	306.69504	204.79912	5
16	2099.02774	1050.01751	700.34743	2127.02265	1064.01496	709.67907	V	515.33003	258.16865	172.44819	4
17	2212.11181	1106.55954	738.04212	2240.10672	1120.55700	747.37376	L	416.26161	208.63444	139.42539	3
18	2340.17039	1170.58883	780.72831	2368.16530	1184.58629	790.05995	Q	303.17754	152.09241	101.73070	2
19							R	175.11896	88.06312	59.04450	1

annotated MSMS spectrum

Extracted from: F:\KW\APAP\FUS02210.raw #21267 RT: 50.92
FTMS, HCD@38.00, z=+3, Mono m/z=848.09656 Da, MH+=2542.27512 Da, Match Tol.=0.05 Da

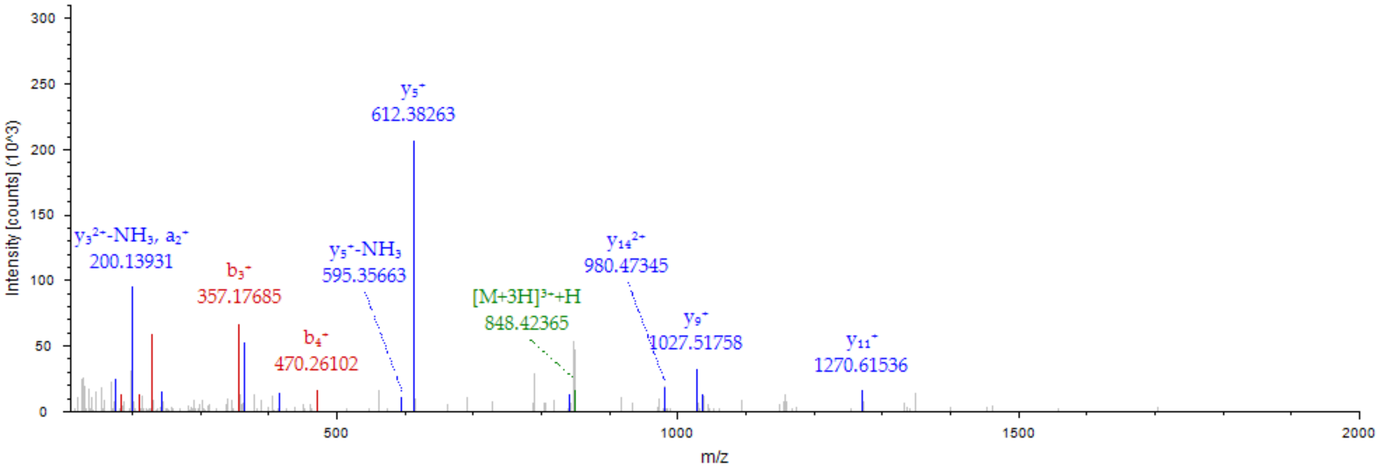

Sequence: QCQCTSVGAQNTVICSK, C2-TMT6-Cys (329.22660 Da), C4-TMT6-Cys (329.22660 Da), C15-TMT6-Cys (329.22660 Da)
Charge: +4, Monoisotopic m/z: 690.12585 Da (+0.96 mmu/+1.39 ppm), MH+: 2757.48159 Da, RT: 24.65 min,
Identified with: Mascot (v1.30); IonScore:19, Exp Value:3.1E-001, Ions matched by search engine: 14/192
Fragment match tolerance used for search: 0.05 Da

b and y ion series

#1	a^{+}	a^{2+}	a^{3+}	a^{4+}	b^{+}	b^{2+}	b^{3+}	b^{4+}	Seq.	y^{+}	y^{2+}	y^{3+}	y^{4+}	#2
1	101.07094	51.03911	34.36183	26.02319	129.06586	65.03657	43.69347	33.02192	Q					17
2	533.30673	267.15700	178.44043	134.08214	561.30164	281.15446	187.77206	141.08087	C-TMT6-	2629.41918	1315.21323	877.14458	658.11025	16
3	661.36531	331.18629	221.12662	166.09678	689.36022	345.18375	230.45826	173.09551	Q	2197.18339	1099.09533	733.06598	550.05131	15
4	1093.60109	547.30418	365.20522	274.15573	1121.59601	561.30164	374.53685	281.15446	C-TMT6-	2069.12481	1035.06604	690.37979	518.03666	14
5	1194.64877	597.82802	398.88778	299.41765	1222.64369	611.82548	408.21941	306.41638	T	1636.88903	818.94815	546.30119	409.97771	13
6	1281.68080	641.34404	427.89845	321.17566	1309.67572	655.34150	437.23009	328.17439	S	1535.84135	768.42431	512.61863	384.71579	12
7	1380.74922	690.87825	460.92126	345.94276	1408.74414	704.87571	470.25290	352.94149	V	1448.80932	724.90830	483.60796	362.95779	11
8	1437.77069	719.38898	479.92842	360.19813	1465.76561	733.38644	489.26005	367.19686	G	1349.74090	675.37409	450.58515	338.19068	10
9	1508.80781	754.90754	503.60746	377.95741	1536.80273	768.90500	512.93909	384.95614	A	1292.71943	646.86335	431.57799	323.93531	9
10	1636.86639	818.93683	546.29365	409.97206	1664.86131	832.93429	555.62529	416.97078	Q	1221.68231	611.34479	407.89895	306.17603	8
11	1750.90932	875.95830	584.30796	438.48279	1778.90424	889.95576	593.63960	445.48152	N	1093.62373	547.31550	365.21276	274.16139	7
12	1851.95700	926.48214	617.99052	463.74471	1879.95192	940.47960	627.32216	470.74344	T	979.58080	490.29404	327.19845	245.65066	6
13	1951.02542	976.01635	651.01333	488.51181	1979.02034	990.01381	660.34496	495.51054	V	878.53312	439.77020	293.51589	220.38874	5
14	2064.10949	1032.55838	688.70802	516.78283	2092.10441	1046.55584	698.03965	523.78156	I	779.46470	390.23599	260.49308	195.62163	4
15	2496.34528	1248.67628	832.78661	624.84178	2524.34019	1262.67373	842.11825	631.84051	C-TMT6-	666.38063	333.69395	222.79839	167.35061	3
16	2583.37731	1292.19229	861.79729	646.59978	2611.37222	1306.18975	871.12892	653.59851	S	234.14484	117.57606	78.71980	59.29167	2
17									K	147.11281	74.06004	49.70912	37.53366	1

annotated MSMS spectrum

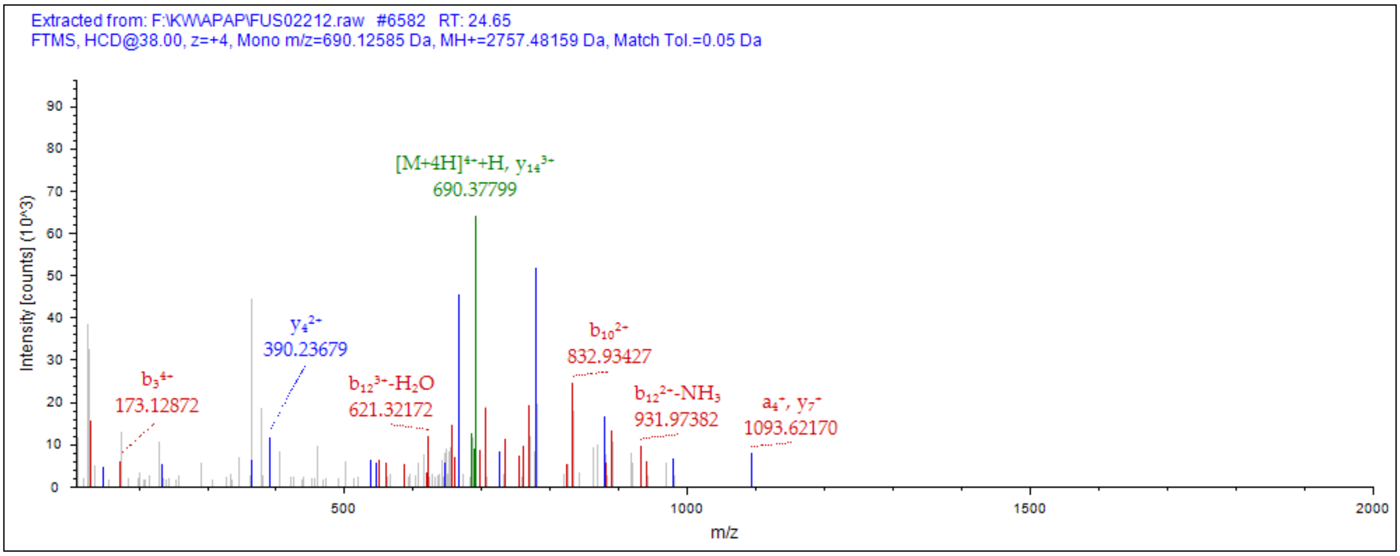

Sequence: GCQDFGWDPCFQPDGYEQTYAEMPK, C2-TMT6-Cys (329.22660 Da), C10-TMT6-Cys (329.22660 Da)
Charge: +4, Monoisotopic m/z: 893.41132 Da (+1.61 mmu/+1.8 ppm), MH+: 3570.62343 Da, RT: 70.05 min,
Identified with: Mascot (v1.30); IonScore:33, Exp Value:9.5E-003, Ions matched by search engine: 18/280
Fragment match tolerance used for search: 0.05 Da

b and y ion series

#1	a^{+}	a^{2+}	a^{3+}	a^{4+}	b^{+}	b^{2+}	b^{3+}	b^{4+}	Seq.	y^{+}	y^{2+}	y^{3+}	y^{4+}	#2
1	30.03383	15.52055	10.68280	8.26392	58.02875	29.51801	20.01443	15.26264	G					25
2	462.26962	231.63845	154.76139	116.32286	490.26453	245.63590	164.09303	123.32159	C-TMT6-	3513.59552	1757.30140	1171.87002	879.15434	24
3	590.32820	295.66774	197.44758	148.33751	618.32311	309.66519	206.77922	155.33624	Q	3081.35974	1541.18351	1027.79143	771.09539	23
4	705.35515	353.18121	235.78990	177.09424	733.35006	367.17867	245.12154	184.09297	D	2953.30116	1477.15422	985.10524	739.08075	22
5	852.42357	426.71542	284.81271	213.86135	880.41848	440.71288	294.14434	220.86008	F	2838.27421	1419.64074	946.76292	710.32401	21
6	909.44504	455.22616	303.81986	228.11672	937.43995	469.22361	313.15150	235.11545	G	2691.20579	1346.10653	897.74011	673.55690	20
7	1095.52436	548.26582	365.84630	274.63655	1123.51927	562.26327	375.17794	281.63528	W	2634.18432	1317.59580	878.73296	659.30154	19
8	1210.55131	605.77929	404.18862	303.39328	1238.54622	619.77675	413.52026	310.39201	D	2448.10500	1224.55614	816.70652	612.78171	18
9	1307.60408	654.30568	436.53954	327.65648	1335.59899	668.30313	445.87118	334.65521	P	2333.07805	1167.04266	778.36420	584.02497	17
10	1739.83986	870.42357	580.61814	435.71542	1767.83478	884.42103	589.94978	442.71415	C-TMT6-	2236.02528	1118.51628	746.01328	559.76178	16
11	1886.90828	943.95778	629.64095	472.48253	1914.90320	957.95524	638.97258	479.48126	F	1803.78949	902.39838	601.93468	451.70283	15
12	2014.96686	1007.98707	672.32714	504.49717	2042.96178	1021.98453	681.65878	511.49590	Q	1656.72107	828.86417	552.91187	414.93573	14
13	2112.01963	1056.51345	704.67806	528.76037	2140.01455	1070.51091	714.00970	535.75909	P	1528.66249	764.83488	510.22568	382.92108	13
14	2227.04658	1114.02693	743.02038	557.51710	2255.04150	1128.02439	752.35202	564.51583	D	1431.60972	716.30850	477.87476	358.65789	12
15	2284.06805	1142.53766	762.02754	571.77247	2312.06297	1156.53512	771.35917	578.77120	G	1316.58277	658.79502	439.53244	329.90115	11
16	2447.13137	1224.06932	816.38198	612.53830	2475.12629	1238.06678	825.71361	619.53703	Y	1259.56130	630.28429	420.52528	315.64578	10
17	2576.17397	1288.59062	859.39618	644.79895	2604.16889	1302.58808	868.72781	651.79768	E	1096.49798	548.75263	366.17084	274.87995	9
18	2704.23255	1352.61991	902.08237	676.81360	2732.22747	1366.61737	911.41401	683.81232	Q	967.45538	484.23133	323.15664	242.61930	8
19	2805.28023	1403.14375	935.76493	702.07552	2833.27515	1417.14121	945.09657	709.07424	T	839.39680	420.20204	280.47045	210.60466	7
20	2968.34355	1484.67541	990.11937	742.84135	2996.33847	1498.67287	999.45101	749.84007	Y	738.34912	369.67820	246.78789	185.34274	6
21	3039.38067	1520.19397	1013.79841	760.60063	3067.37559	1534.19143	1023.13005	767.59935	A	575.28580	288.14654	192.43345	144.57691	5
22	3168.42327	1584.71527	1056.81261	792.86128	3196.41819	1598.71273	1066.14425	799.86000	E	504.24868	252.62798	168.75441	126.81763	4
23	3299.46377	1650.23552	1100.49278	825.62140	3327.45869	1664.23298	1109.82441	832.62013	M	375.20608	188.10668	125.74021	94.55698	3
24	3396.51654	1698.76191	1132.84370	849.88459	3424.51146	1712.75937	1142.17534	856.88332	P	244.16558	122.58643	82.06004	61.79685	2
25									K	147.11281	74.06004	49.70912	37.53366	1

annotated MSMS spectrum

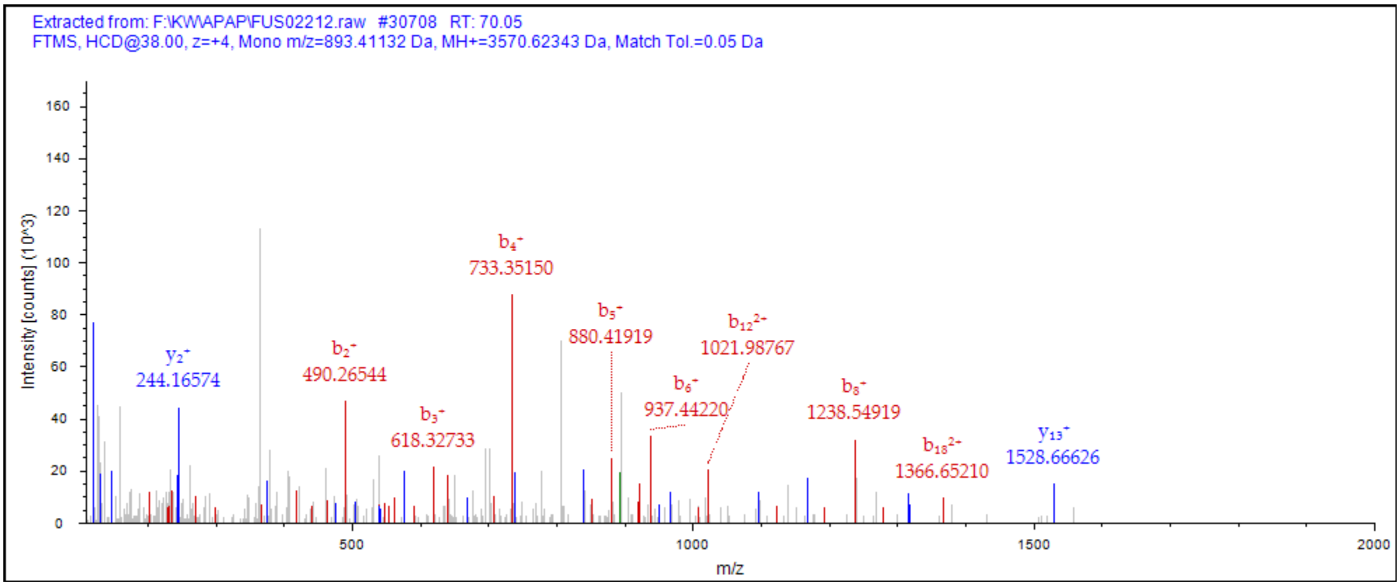

Sequence: LYSNAYLNDLAGCIK, C13-TMT6-Cys (329.22660 Da)
Charge: +3, Monoisotopic m/z: 663.02246 Da (+0.25 mmu/+0.38 ppm), MH+: 1987.05283 Da, RT: 64.32 min,
Identified with: Mascot (v1.30); IonScore:55, Exp Value:1.2E-004, Ions matched by search engine: 7/156
Fragment match tolerance used for search: 0.05 Da

b and y ion series

#1	a^+	a^{2+}	a^{3+}	b^+	b^{2+}	b^{3+}	Seq.	y^+	y^{2+}	y^{3+}	#2
1	86.09643	43.55185	29.37033	114.09135	57.54931	38.70197	L				15
2	249.15975	125.08351	83.72477	277.15467	139.08097	93.05641	Y	1873.96800	937.48764	625.32752	14
3	336.19178	168.59953	112.73545	364.18670	182.59699	122.06708	S	1710.90468	855.95598	570.97308	13
4	450.23471	225.62099	150.74976	478.22963	239.61845	160.08139	N	1623.87265	812.43996	541.96240	12
5	521.27183	261.13955	174.42880	549.26675	275.13701	183.76043	A	1509.82972	755.41850	503.94809	11
6	684.33515	342.67121	228.78324	712.33007	356.66867	238.11487	Y	1438.79260	719.89994	480.26905	10
7	797.41922	399.21325	266.47793	825.41414	413.21071	275.80956	L	1275.72928	638.36828	425.91461	9
8	911.46215	456.23471	304.49224	939.45707	470.23217	313.82387	N	1162.64521	581.82624	388.21992	8
9	1026.48910	513.74819	342.83455	1054.48402	527.74565	352.16619	D	1048.60228	524.80478	350.20561	7
10	1139.57317	570.29022	380.52924	1167.56809	584.28768	389.86088	L	933.57533	467.29130	311.86329	6
11	1210.61029	605.80878	404.20828	1238.60521	619.80624	413.53992	A	820.49126	410.74927	274.16860	5
12	1267.63176	634.31952	423.21544	1295.62668	648.31698	432.54708	G	749.45414	375.23071	250.48956	4
13	1699.86755	850.43741	567.29403	1727.86246	864.43487	576.62567	C-TMT6-	692.43267	346.71997	231.48241	3
14	1812.95162	906.97945	604.98872	1840.94653	920.97690	614.32036	I	260.19688	130.60208	87.40381	2
15							K	147.11281	74.06004	49.70912	1

annotated MSMS spectrum

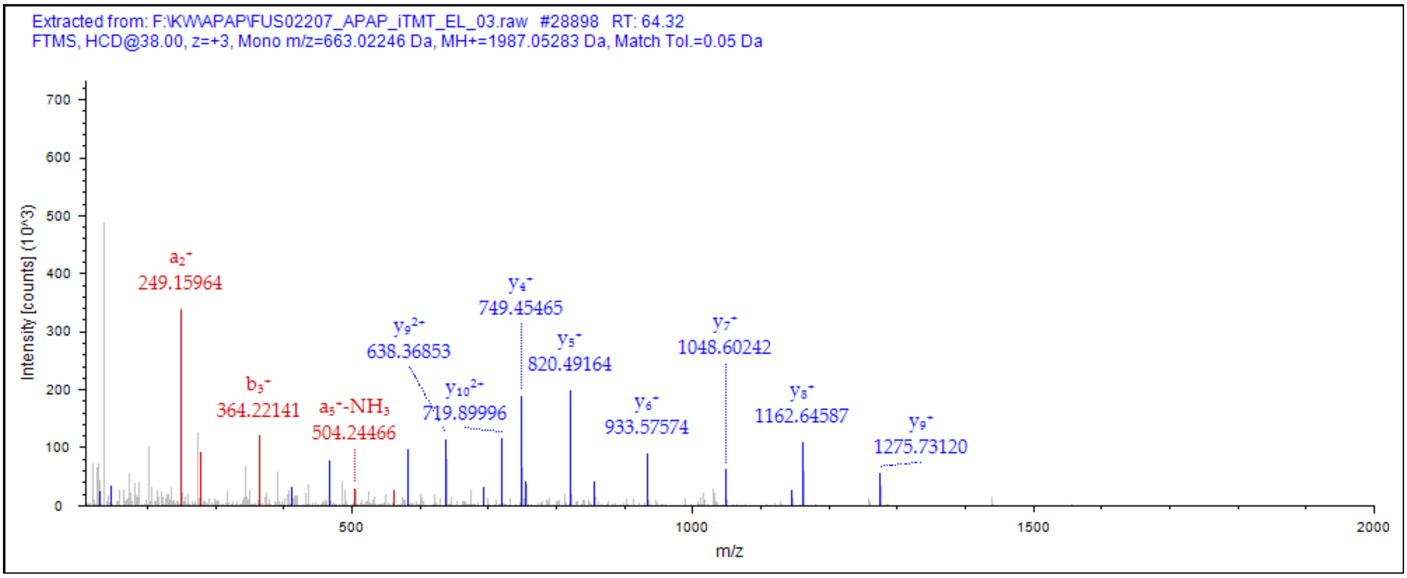

Supplement: Supplementary file 1 [file TX-005-C5TX00469A-s001.pdf]
